# Conditional Degradation of *Plasmodium* Calcineurin Reveals Functions in Parasite Colonization of both Host and Vector

**DOI:** 10.1016/j.chom.2015.05.018

**Published:** 2015-07-08

**Authors:** Nisha Philip, Andrew P. Waters

**Affiliations:** 1 Wellcome Trust Centre for Molecular Parasitology, Institute of Infection, Immunity and Inflammation, College of Medical Veterinary & Life Sciences, University of Glasgow, Glasgow, G12 8TA, UK

## Abstract

Functional analysis of essential genes in the malarial parasite, *Plasmodium*, is hindered by lack of efficient strategies for conditional protein regulation. We report the development of a rapid, specific, and inducible chemical-genetic tool in the rodent malaria parasite, *P. berghei*, in which endogenous proteins engineered to contain the auxin-inducible degron (AID) are selectively degraded upon adding auxin. Application of AID to the calcium-regulated protein phosphatase, calcineurin, revealed functions in host and vector stages of parasite development. Whereas depletion of calcineurin in late-stage schizonts demonstrated its critical role in erythrocyte attachment and invasion in vivo, stage-specific depletion uncovered roles in gamete development, fertilization, and ookinete-to-oocyst and sporozoite-to-liver stage transitions. Furthermore, AID technology facilitated concurrent generation and phenotyping of transgenic lines, allowing multiple lines to be assessed simultaneously with significant reductions in animal use. This study highlights the broad applicability of AID for functional analysis of proteins across the *Plasmodium* life cycle.

## Highlights

• Calcineurin regulates colonization of host cells across the Plasmodium life cycle
• Calcineurin regulates male gametogenesis
• AID technology is broadly applicable to study protein function in Plasmodium
• Multiplexing of AID technology results in substantially reduced animal use

## Graphical Abstract

**Figure:**
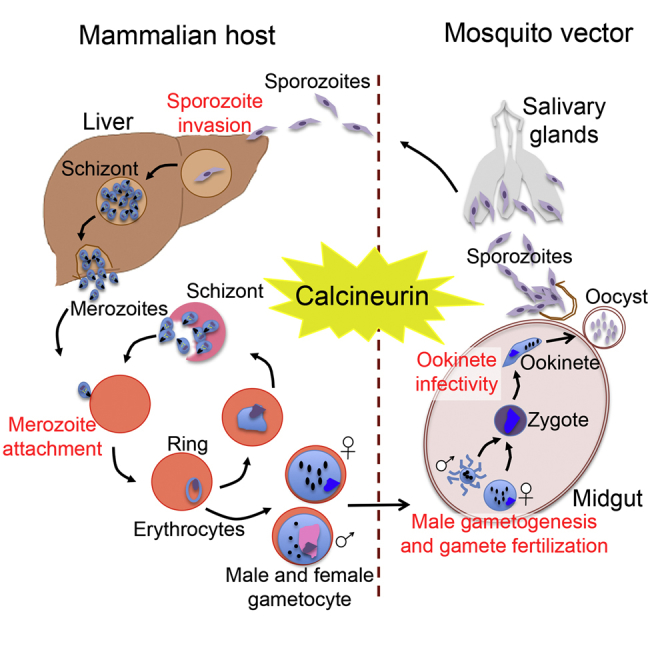

## Teaser

Limited conditional regulation technologies in the malarial parasite, Plasmodium, hinder functional examination of parasite proteins operating at multiple stages. Philip and Waters report the application of a rapid and specific conditional protein degradation tool to study parasite Calcineurin function in both host and vector stages of the parasite life cycle.

## Introduction

While completing its complex life cycle, *Plasmodium*, the causative agent of malaria, experiences diverse host environments and undergoes remarkable variation in shape, size, and motility. Parasite development and response to environmental cues are controlled by signaling cascades, many of which are regulated by calcium (Billker et al., 2009). Although calcium is a ubiquitous intracellular messenger in numerous organisms, the malarial parasite maintains both conserved and evolutionarily unique calcium effectors modulating protein phosphorylation. One class of unique effectors are the calcium-dependent protein kinases (CDPKs), a family of protein kinases characteristic of plants and alveolates (Harmon et al., 2000). CDPKs translate calcium signals into diverse outputs, including translational control, microneme secretion, schizont egress, ookinete motility, and liver stage invasion (Bansal et al., 2013; Dvorin et al., 2010; Ishino et al., 2006; Sebastian et al., 2012; Siden-Kiamos et al., 2006).

In addition to CDPKs, fine-tuning of these calcium-dependent processes is thought to require protein phosphatases. In contrast to six or seven CDPKs (depending on species), only two *Plasmodium* phosphatases, protein phosphatase 7 and calcineurin, have calcium-binding motifs, suggesting they modulate several calcium-dependent biological processes (Wilkes and Doerig, 2008). Calcineurin is a heterodimeric protein comprising of a catalytic (CnA) and calcium-binding regulatory (CnB) subunit (Rusnak and Mertz, 2000). Gene expression of both subunits is observed at specific life cycle points in both host and vector stages of the parasite, strongly suggesting a multistage functional profile for this phosphatase and a role in parasite transitions through the life cycle (Otto et al., 2014).

The difficulty in studying multifunctional proteins and proteins essential for intraerythrocytic development in *Plasmodium* is the lack of robust conditional knockout strategies. Although conditional gene deletion was demonstrated in both *P. falciparum* and *P. berghei*, gene removal is irreversible and examines the function of the corresponding protein only at its first point of action (Collins et al., 2013; Combe et al., 2009). Methodologies that inducibly regulate transcription have also been described, but these suffer from slow onset kinetics and have been tested only for asexual stage development (Meissner et al., 2005; Pino et al., 2012). Recently, an inducible protein-RNA-based interaction system was reported where the authors show a robust 80% reduction in protein expression (Goldfless et al., 2014). Although this system will have broad implications in examining protein function in various subcellular contexts, the technique nevertheless has to contend with stability of the protein, which was synthesized prior to imposing regulation. By contrast, direct manipulation of protein levels by chemical-genetic methods can offer significant advantages of inducibility, speed, and specificity. Two such regulatory systems have been developed in *P. falciparum* to regulate protein stability either using FK506-binding protein or a dihydrofolate reductase destabilization domain (Armstrong and Goldberg, 2007; Muralidharan et al., 2011). Unfortunately, these drug-on methods for inducible stabilization require constant application of the small molecule when generating and maintaining transgenic lines and therefore are frequently unsuitable for use in in vivo settings. Moreover, drug-on methods are difficult to maintain in the non-erythrocytic life stages.

Here we report the development and application of a rapid and specific protein degradation tool to examine protein function in *P. berghei.* We adapted an inducible protein depletion method that relies on the proteasome-mediated auxin response pathway in plants (Nishimura et al., 2009). By successful application of this chemical-genetic method, we dissect and reveal the functions of the essential gene, calcineurin, at key transition points of the *Plasmodium* life cycle. We show that calcineurin regulates parasite colonization of diverse host cell types, including erythrocytes, mosquito midgut cells, and hepatocytes, demonstrating the versatility of this technology. We further engineered the degradation system to promote multiplex transgenic parasite generation combined with phenotype analysis. Hence, this inducible, specific, and rapid protein degradation technology significantly enhances the *Plasmodium* research tool kit to study multifunctional or essential genes and provides the community with a resource to facilitate targeted genetic screens.

## Results

### Auxin-Inducible Degron System Enables Conditional Expression of Calcineurin A in *P. berghei*

**Figure 1:**
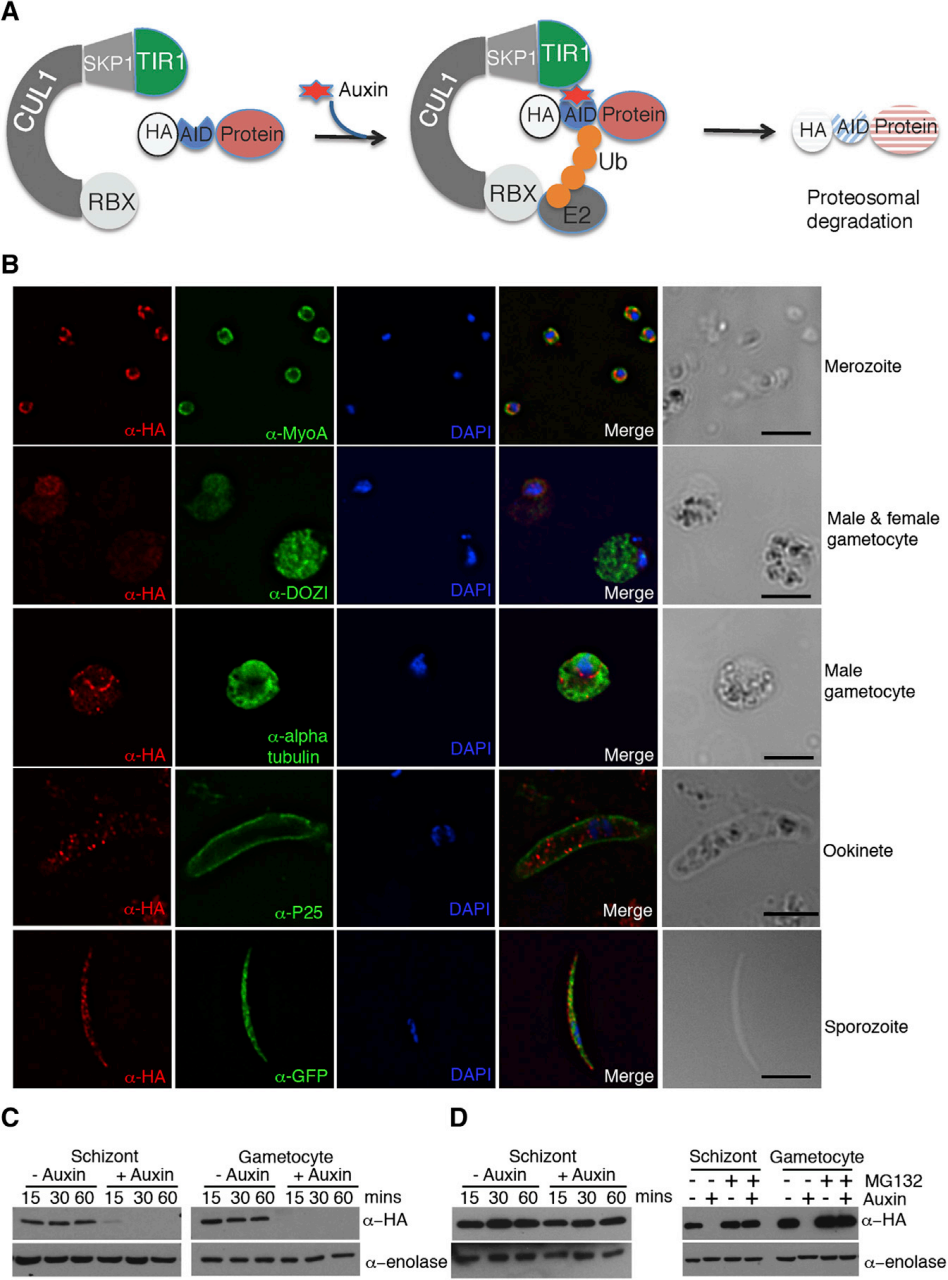
Generation of a Functional AID System in *Plasmodium berghei* to Examine Calcineurin Function (A) Auxin promotes interaction of TIR1 (an F box protein, in green) with the AID degron tagged protein (blue). The AID-tagged protein (red) is recruited to the Skp, Cullin, F-box-containing complex (SCF), a multi-protein E3-ligase complex, resulting in ubiquitination and degradation of the target protein. Schematic adapted from Nishimura et al. (2009). (B) Expression and localization of PbCnA-AID-HA at the indicated stages of *Plasmodium* life cycle. Fixed and permeablised parasites were probed with indicated primary antibodies. Scale bar, 5 μm. (C) Robust and efficient depletion of PbCnA-AID-HA, upon addition of auxin in both schizonts and gametocytes, as measured by western blotting. Enolase serves as a loading control. (D) Conditional depletion of PbCnA-AID-HA is reliant on auxin, TIR1, and the proteasome. PbCnA-AID protein levels in a non-TIR1 background is resistant to auxin-mediated depletion (left panel). Pre-incubation with proteasome inhibitor 1 μM MG132 for 1 hr blocks PbCnA-AID depletion by auxin (right panel), as shown by western blotting. Enolase serves as a loading control. See also Figure S1.

Inability to delete CnA (our efforts and Guttery et al., 2014) and the expression profile of the phosphatase at key transition stages of the parasite life cycle (Lindner et al., 2013; Otto et al., 2014; Patra et al., 2008) prompted us to develop a rapid and specific protein regulation tool. The system relies on the highly conserved Skp1, Cullin1, F box protein ubiquitin ligase (SCF) complex where the F box protein recruits specific substrates for degradation. Auxin functions as a molecular glue that promotes and stabilizes physical interaction between the auxin receptor, TIR1 (an F box protein), and proteins containing an auxin-inducible degron (AID) motif (Nishimura et al., 2009) (Figure 1A). Although the TIR1 F box protein is specific to plants, the high degree of conservation of eukaryotic Skp1 proteins is predicted to allow association with ectopically expressed TIR1 to form a functional SCF^TIR^ complex in *Plasmodium* (Figures 1A, S1A, and S1B). We generated two marker-free parent lines expressing *ostir1* controlled by the strong ubiquitously expressing *hsp70* promoter (Figures S1C–S1E) and *pb48/45* or *pb28* 3′ UTRs. The *pb48/45* 3′ UTR controlled line was well suited for phenotyping of blood stages, but it produced lower ookinete numbers. This was resolved by utilizing the *pb28* 3′ UTR controlled TIR1 for post-gamete fertilization assays, including motility, microneme secretion, and infectivity of ookinetes and liver stage development of sporozoites. In the OsTIR1-expressing parent lines, *pbcna* (PBANKA_122740) was tagged at the C terminus by *aid-2xha* using single cross-over recombination, which was confirmed by PCR and western blotting (Figures S1F and S1G). Immunofluorescence and western blotting indicated that CnA was expressed at the schizont/merozoite, gametocyte, and sporozoite stages of the parasite life cycle (Figures 1B and 1C). CnA protein was localized to the cytoplasm in all stages. However, in gametocytes it was detected only in males where, in addition to diffused cytoplasmic expression, the protein appeared to form a ring around the nucleus, suggesting CnA has different and/or additional functions in male gametocytes.

Next we tested if CnA fused to the AID-2xHA degron (CnA-AID) could be depleted at both asexual and sexual life cycle stages in an auxin-dependent manner. Schizonts, gametocytes, or sporozoites were incubated with 500 μM auxin for the indicated times. Auxin stimulated efficient degradation of CnA-AID fusions at each of these stages within only 45 min (Figures 1C and S3C) and was dependent on both TIR1 and the proteasome (Figure 1D).

### Calcineurin Regulates Merozoite Invasion of Erythrocytes In Vivo

**Figure 2:**
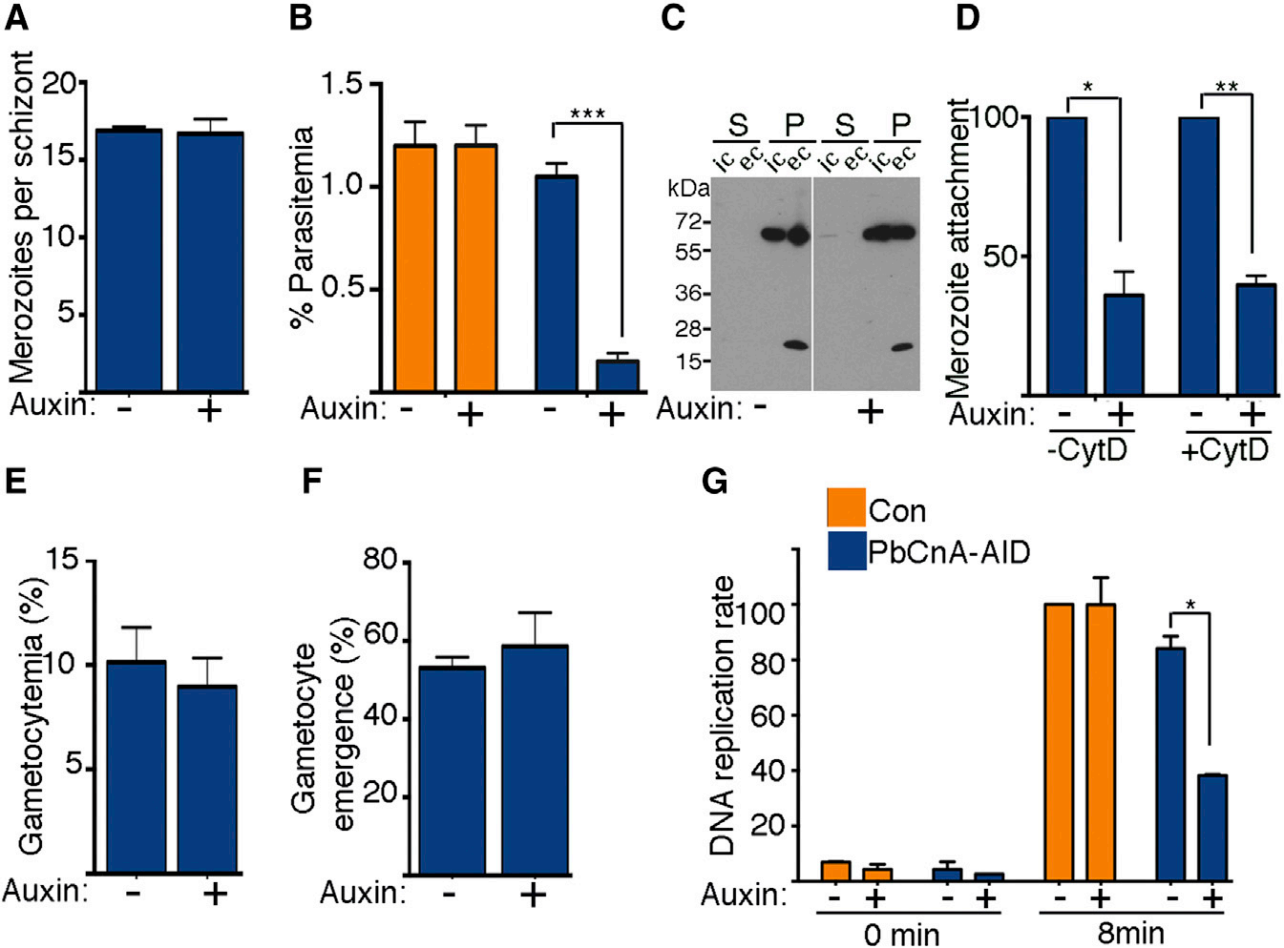
Calcineurin Regulates Erythrocyte Invasion by the Merozoite and Male Gametogenesis (A) Sustained (24.5 hr) auxin treatment from the ring stage has no effect on the number of merozoites produced in PbCnA-AID parasites (25 segmented schizonts counted per condition, n = 4 experiments). (B) Pre-incubation of mature PbCnA-AID schizonts with auxin for 30 min results in 86% reduction of in vivo erythrocytic invasion by merozoites as measured by flow cytometry (n = 3 experiments). (C) AMA1 processing in free merozoites (± auxin) determined by exposing merozoites to extracellular conditions ([ec]; high Na^+^ and low K^+^) versus intracellular conditions ([ic]; low Na^+^ and high K^+^) and measured by western blotting. Both supernatant (S) and pellet (P) fractions were probed with mAb28G2, which recognizes the C terminus of AMA-1. (D) Merozoite attachment to erythrocytes is regulated by PbCnA-AID. Isolated merozoites (± auxin; ± CytD) were incubated with erythrocytes under shaking conditions. After 10 min, cells were fixed and merozoite attachment/invasion was assessed by microscopy (n = 3 experiments with at least ten fields each containing ∼350 erythrocytes per experimental condition). See also Figures S2C–S2E. (E) Gametocyte production is unaffected by PbCnA-AID depletion. Synchronized ring-stage parasites were treated with auxin (15 min post-invasion) and grown ex-vivo, and gametocytemia was determined 32 hr later (n = 4 experiments). (F) Gametocyte emergence from erythrocytes is unaffected by PbCnA-AID depletion. Gametocytes were pre-treated with auxin for 45 min and subsequently activated for gametogenesis by addition of RPMI + 100 μM xanthurenic acid and a drop in temperature from 37°C to 21°C (n = 2 experiments with 100 gametocytes counted per experimental condition). (G) Reduction in the number of male gametocytes replicating their DNA with PbCnA-AID depletion. Proportion of male gametocytes undergoing DNA replication was determined at 0 and 8 min post-activation and is expressed as a percentage of the non-auxin-treated control line at 8 min (n = 3 experiments). Control lines (Con) are OsTIR1 expressing with an unmodified *pbcna* locus (pG230). For all panels, auxin = 500 μM IAA, mean ± SEM; two-tailed t test for paired observations: [^∗^], p < 0.05; [^∗∗^], p < 0.01; and [^∗∗∗^],p < 0.001. See also Figure S2.

Previous work using indirect Calcineurin inhibitors such as cyclosporin A and FK506 have suggested that Calcineurin is essential during blood stage development. (Dobson et al., 1999; Singh et al., 2014). However, cyclosporin A and FK506 target Calcineurin by associating with either cyclophilins or FK506-binding protein, both of which exhibit prolyl isomerase activity. Therefore, the phenotype observed upon addition of these inhibitors could in part be a pleiotropic effect of prolyl isomerase inhibition. Thus, by specifically depleting CnA protein levels with the AID system, we expected to gain a clearer understanding of its protein function. Indeed, addition of auxin to synchronized ring stage parasites completely depleted CnA levels in schizonts but did not influence intraerythrocytic development from rings into schizonts (Figures S2A and S2B). Moreover, schizonts generated from auxin-treated rings had a normal complement of merozoites, further indicating that CnA is not required for erythrocytic schizogony (Figure 2A). However, when CnA-AID was depleted in mature schizonts and isolated merozoites were intravenously administered to naive mice, we observed a nearly 90% reduction in erythrocyte invasion (Figures 2B and S2A). Hence, AID-mediated depletion of CnA demonstrated the importance of this phosphatase for invasion of erythrocytes by the parasite under flow conditions in vivo.

### Calcineurin Specifically Controls Merozoite Attachment to the Erythrocyte

Merozoite invasion of the erythrocyte is a complex multistep process requiring several merozoite-erythrocyte attachment phases and release of secretory organelles (reviewed in Cowman et al., 2012). To dissect the point of Calcineurin action during merozoite invasion, we developed an in vitro assay to examine processing of the micronemal protein, PbAMA1 (based on Singh et al., 2014), and also to inspect merozoite attachment to the erythrocyte (based on the accompanying manuscript, Paul et al., 2015). Plasmodium AMA1, a transmembrane protein (83 kDa in Pf and 66 kDa in Pb), undergoes multiple proteolytic processing events and is exported to the merozoite surface upon egress from the host cell (Howell et al., 2001; Kocken et al., 1998; Peterson et al., 1989). During erythrocyte invasion the cleaved fragments are shed from the parasite surface while the C-terminal domain is retained in the parasite membrane (Howell et al., 2001). Due to lack of antibodies recognizing the secreted PbAMA-1 fragments, we utilized the C-terminal reactive mAB3G2 to investigate proteolytic processing of PbAMA1. Merozoites were isolated and incubated in either intracellular ([IC]: low Na^+^ and high K^+^) or extracellular (EC) buffer (high Na^+^ and low K^+^). Only under EC conditions is the ∼20 kDa band corresponding to the C-terminal fragment of PbAMA1 observed (Figure 2C). While the assay could not examine secreted PbAMA1 fragments, it demonstrated that depletion of PbCnA-AID had no detectable effect on levels of the cleaved and membrane-bound PbAMA1 fragment (Figure 2C).

To determine whether calcineurin influenced merozoite attachment or subsequent entry into the host cell, we utilized an actin inhibitor, Cytochalisin D (CytD), which permits merozoite attachment to the erythrocyte but prevents entry (Miller et al., 1979). We incubated purified merozoites with erythrocytes under shaking conditions for 10 min and immediately examined merozoite attachment/invasion of the erythrocyte by microscopy (Figure S2C). Upon PbCnA depletion, both in the presence and absence of CytD, merozoite attachment/invasion to erythrocytes was significantly and equally reduced (Figures 2D and S2D). Furthermore, when ring stage parasitemia resulting from successfully invaded merozoites was assessed 4 hr post-invasion, the reduction in parasitemia was similar to the attachment defect (Figures 2D and S2E).

Taken together, these data reveal that while PbAMA1 processing is independent of calcineurin, the defect in erythrocyte invasion is largely contributed by the influence of calcineurin activity on merozoite attachment to the erythrocyte.

### Calcineurin Regulates Male Gametogenesis and Gamete Fertilization

Approximately 5%–20% of blood stage parasites form differentiation-arrested male and female gametocytes, which circulate with asexual stage parasites in the infected host. In vitro conditions that mimic the mosquito vector environment, including a drop in temperature and addition of xanthurenic acid, induce calcium signals in male and female gametocytes to stimulate gametogenesis (Billker et al., 2004; Billker et al., 1998). Upon activation, both male and female gametocytes emerge from erythrocytes, followed by male gametocytes undergoing three rounds of genomic DNA replication, forming eight nuclei that are each packaged into an axoneme-containing microgamete (Janse et al., 1986). Auxin-induced depletion of CnA-AID in ex vivo ring-stage parasites did not influence gametocyte production and maturation (Figure 2E). Similarly, CnA-AID depletion in mature gametocytes prior to activation did not affect gametocyte emergence as assayed by rupture of infected erythrocytes (Figures 2F and S2H). Additionally, in the absence of CnA-AID, typical expression of the translationally repressed ookinete surface protein, P28, and Rab11a GTPase, both of which are markers of activated female gametes, indicated normal female gametogenesis (Figures S2F and S2G). However, CnA-AID depletion in mature gametocytes prior to activation acutely affected male gametogenesis. We observed a ∼50% reduction in male gametocytes undergoing DNA replication (Figures 2G and S2I), while the remaining male gametocytes exhibited normal DNA replication and further develop into microgametes (Figure S2J).

**Figure 3:**
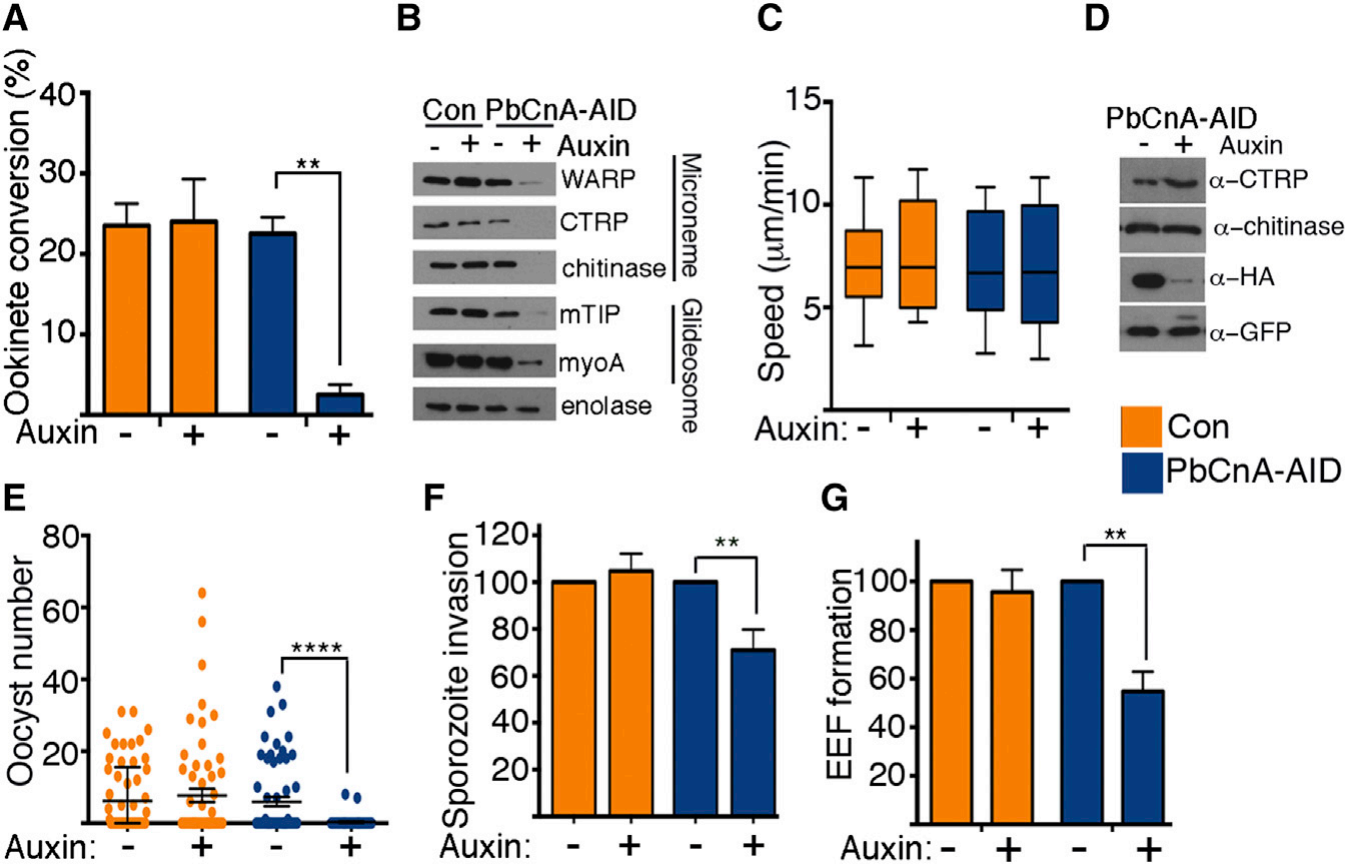
Calcineurin Regulates Gamete Fertilization and Host Cell Colonization of both Ookinetes and Sporozoites (A) PbCnA-AID depletion in gametocytes resulted in 90% reduction in ookinetes formed. Conversion rate is reported as the percentage of female gametes forming ookinetes (n = 4 experiments). (B) Following PbCnA-AID depletion prior to gametocyte activation, ookinete micronemal and motor proteins, known to be expressed post-fertilization, become nearly undetectable by western blot. Enolase serves as the loading control. (C) Ookinete motility (measured by distance covered over time) is unaffected by PbCnA-AID depletion (n = 21 instances per condition; bottom and top of box denote first and third quartiles, respectively; whiskers denote minimum and maximum; p = 0.9 for PbCnA-AID line ± auxin, 0.6 for Con line ± auxin). (D) Secretion of the micronemal proteins CTRP and chitinase into the supernatant is unaffected by PbCnA-AID depletion in mature ookinetes. (E) Oocyst numbers in mosquito midgut upon PbCnA-AID depletion in mature ookinetes. Auxin was added to parasite cultures 6 hr post-induction of gametogenesis. Mature ookinetes were fed to mosquitoes, and 7 days later, midguts were dissected and GFP-positive oocysts were counted (n = minimum of 50 mosquitoes for each condition; mean ± SD). Also see independent repeat in Figure S3B. (F) Sporozoite invasion was examined by incubating mosquito salivary gland sporozoites with auxin (90 min), followed by addition to HepG2 hepatocytes. Sporozoite invasion was assessed 2 hr later and calculated as the proportion of intracellular sporozoites to total sporozoites (intracellular + extracellular). For both control and experimental line, 100% is the proportion of intracellular sporozoites without auxin treatment (n = 2 experiments). (G) Incubation with auxin (90 min) of mosquito salivary gland sporozoites from a GFP-expressing version of PbCnA-AID parasites reduces EEFs in HepG2 hepatocytes by 46%. For both control and experimental line, 100% is the number of GFP-positive EEFs without auxin treatment (n = 3 experiments). For all panels, auxin denotes 500 μM IAA; control line (Con) is the TIR1-expressing parent (pG230 for [A] and [B); pG402 for [C]–[G]); mean ± SEM; two-tailed t test for paired observations: [^∗∗^], p < 0.01; [^∗∗∗∗^], p < 0.0001. See also Figure S3.

In fertilization, a wild-type male microgamete fuses with a female macrogamete to form a zygote. In a population of gametocytes, auxin-induced depletion of CnA-AID reduced the ookinete forms developed from a fertilized zygote by 90% (Figure 3A). Consequently, the levels of both micronemal and glideosome proteins expressed post-fertilization were significantly reduced or abolished (Figure 3B). When compared to the 50% defect in male gametocyte genome replication (Figure 2G), the additional defect in fertility observed with CnA-AID depletion argues that CnA also directly regulates the function or fertility of the remaining apparently mature male gametes (Figure S2J) Taken together, the above data and male gametocyte restricted protein expression (Figure 1B) demonstrate a specific role for CnA in regulating male gametogenesis and subsequent fertilization.

### Calcineurin Regulates Life Cycle Transition in Ookinetes and Sporozoites

The *Plasmodium* life cycle has two motile stages: an ookinete stage that is required for switching from the mammalian host to the vector and a sporozoite stage for transitioning from the mosquito vector to the host. Calcium regulates invasion and motility of both these infective stages (Coppi et al., 2007; Siden-Kiamos et al., 2006). Since CnA is expressed in both mature ookinetes and sporozoites, we investigated if it influenced specific characteristics of these motile and invasive forms. When auxin was added 4–6 hr post-fertilization, normal ookinete development was observed, and CnA-AID depletion did not affect ookinete motility or secretion of micronemal proteins such as CTRP or chitinase (Figures 3C, 3D, and S3A) (Philip et al., 2012). However, when CnA-AID-depleted ookinetes were fed to mosquitoes, we observed a significant decrease in oocyst formation in the mosquito midgut, implying a role for CnA in ookinete-to-oocyst transition (Figures 3E and S3B). In order to examine CnA function in the sporozoite, 22 day sporozoites isolated from salivary glands were incubated with auxin for 90 min and deposited on HepG2 liver cells. Utilizing a differential staining method (Sinnis et al., 2013) to distinguish intracellular and extracellular sporozoites, upon CnA-AID depletion, we observed a 29% reduction in sporozoite invasion of HepG2 hepatocytes (Figure 3F). However, CnA-AID depletion reduced the number of exo-erythrocytic forms (EEFs) by 46%, indicating calcineurin requirement during liver stage development (Figures 3G and S3C) and further implying that not all sporozoites that successfully invade hepatocytes develop to recognizable EEFs. Any observed EEFs exhibited normal development (Figures 3G and S3D). The decrease in oocyst and EEF formation due to reduced colonization capacity of ookinetes and sporozoites, respectively, demonstrates the requirement for CnA when the parasite undergoes key life cycle transitions between the host and vector.

### Depletion of CDPK1 and PPKL Using AID Replicates Gene Deletion Studies

To demonstrate broader applicability of the AID protein depletion system, we applied the technology to two protein phosphorylation-modulating enzymes with published gene manipulation studies. The two targets were CDPK1, which is the focus of significant drug development efforts, and the protein phosphatase with kelch-like (PPKL) domains.

**Figure 4:**
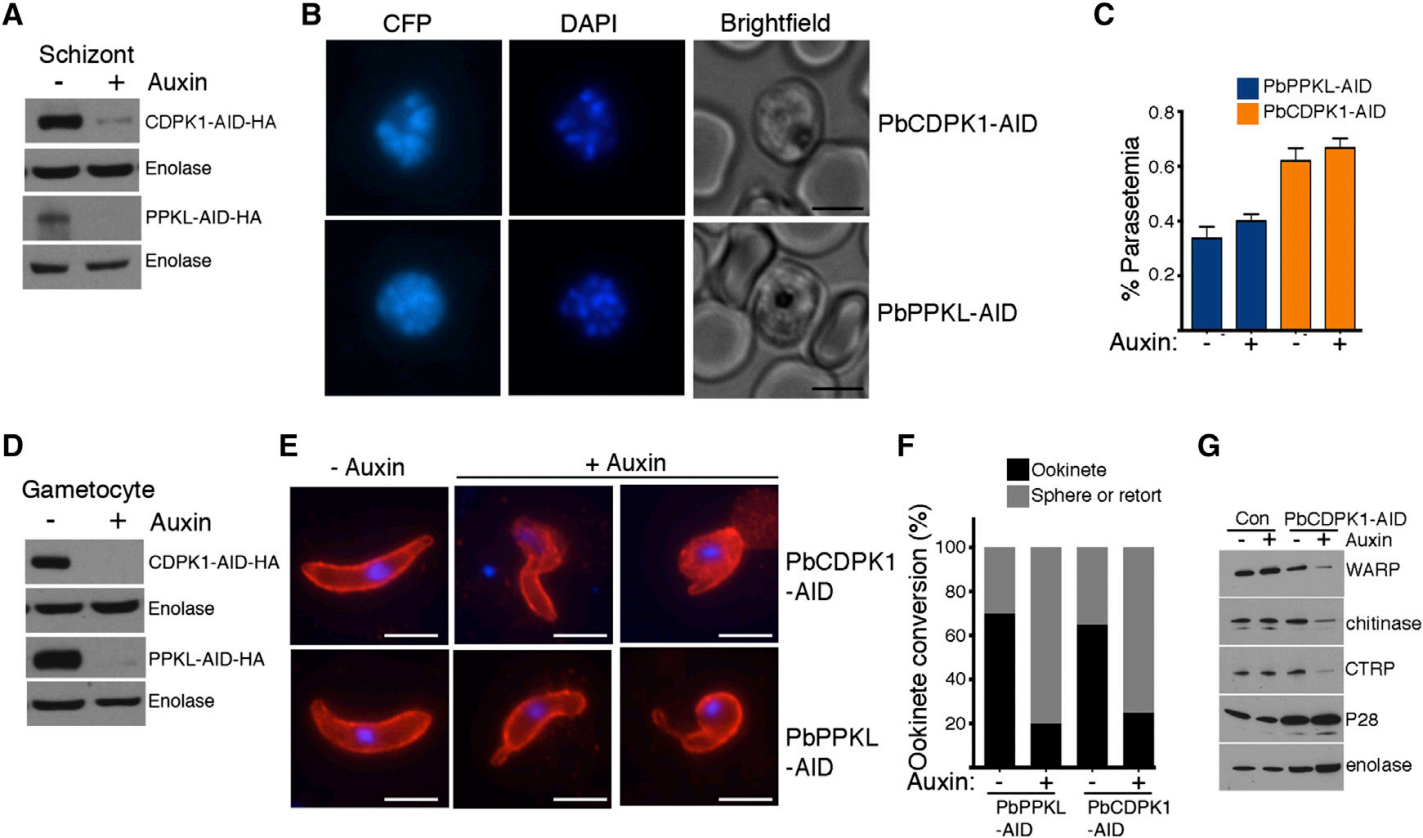
Conditional Protein Depletion Demonstrates CDPK1 and PPKL Are Dispensable for Asexual Growth but Crucial for Ookinete Development (A) Robust depletion of both CDPK1 and PPKL protein with C-terminal AID-HA epitope tags, upon addition of auxin for 45 min in schizonts, as measured by western blotting. Enolase serves as the loading control. (B) Continuous treatment with auxin (24.5 hr) from ring stage has no effect on schizogony in both PbCDPK1-AID and PbPPKL-AID parasite lines. Scale bar, 5 μm. (C) Pre-incubation of mature PbCDPK1-AID or PbPPKL-AID schizonts with auxin for 45 min had no effect on erythrocytic invasion by merozoites as measured by flow cytometry (n = 3 experiments) (D) Robust depletion of both CDPK1 and PPKL protein with C-terminal AID-HA epitope tags, upon addition of auxin for 45 min in gametocytes, as measured by western blotting. Enolase serves as the loading control. (E) PbCDPK1-AID or PbPPKL-AID depletion in gametocytes resulted in abnormal ookinete formation. Zygote-to-ookinete development was visualized by probing against the surface protein marker, P25 (in red, DAPI: blue). (F) Proportion of spherical/retort and normal ookinete forms observed upon PbPPKL or PbCDPK1 depletion. Gametocytes were pre-incubated with auxin for 45 min followed by induction of gametogenesis. Zygote-to-ookinete conversion was determined by examining and counting cells stained with α-P25 antibody. Scale bar, 5 μm. (G) CDPK1 regulates protein levels of translationally controlled genes during zygote-to-ookinete development. Gametocytes were incubated with auxin for 45 min prior to induction of gametogenesis. 24 hr later, parasite pellets were examined by western blotting. While expression of P-28 was unaffected in PbCDPK1-AID-depleted parasites, WARP, CTRP, and Chitinase levels were significantly reduced. Enolase serves as loading control. See also Figure S4.

Employing a similar strategy used to generate PbCnA-AID tagged parasites, we C-terminally tagged endogenous *pbcdpk1* and *pbppkl* with the *aid* degron (Figures S4A–S4C). Treatment with auxin for 45 min led to strong depletion of CDPK1-AID and PPKL-AID proteins in both schizonts and gametocytes stages, thereby confirming the application potential of this technology to both proteins and stages (Figures 4A and 4D). Previous gene deletion studies indicated *P. berghei* CDPK1 and PPKL are not essential for the parasite’s asexual blood-stage development (Guttery et al., 2012; Jebiwott et al., 2013; Philip et al., 2012). Similarly, in our experiments sustained application of auxin starting at ring stage parasites of both lines had no effect on schizogony (Figure 4B). Moreover, depletion of either CDPK1 or PPKL in mature schizonts, followed by intravenous administration to mice, did not influence erythrocyte invasion (Figure 4C). Gene deletion (*ppkl*) and conditional expression (*cdpk1*) in *P. berghei* established that PPKL and CDPK1 regulate ookinete morphology (Guttery et al., 2012; Philip et al., 2012; Sebastian et al., 2012). Depletion of PPKL-AID or CDPK1-AID in mature gametocytes followed by induction of gametogenesis also resulted in abnormal ookinete forms comprising primarily of spheres or retorts (Figures 4E and 4F). During zygote-to-ookinete development, CDPK1 influences translationally controlled expression of ookinete proteins. Similarly, CDPK1-AID depletion displayed significantly reduced levels of several ookinete micronemal proteins, including WARP, chitinase, and CTRP (Figure 4G). Collectively, the phenotypic analysis of asexual and sexual stage development by the AID technology largely mirrors the gene deletion and manipulation reports for both PbCDPK1 and PbPPKL, indicating its broad applicability to studies of gene function in *P. berghei*.

### A Multiplexed AID System for Medium-Throughput Phenotyping

The traditional approach to generate a cloned transgenic parasite line requires individually injecting single IRBCs into ten naive mice. Recently, a flow-cytometry-assisted method was used to isolate isogenic parasite lines resulting in a reduction of animal use by 80% (Kenthirapalan et al., 2012). The AID-tagging plasmid expressing a fluorescent marker can be exploited similarly to isolate isogenic lines by FACs instead of the traditional cloning method (Figure S4A). The bidirectional eef1α promoter was used to drive both drug selection and fluorescent markers, thereby avoiding the use of two separate promoters. Two significant features of the AID-tagging plasmid, which include the fluorescence marker and generation of a regulatable target protein, lend this technology to medium-throughput generation and phenotyping of transgenic parasite lines and a further reduction in animal use.

**Figure 5:**
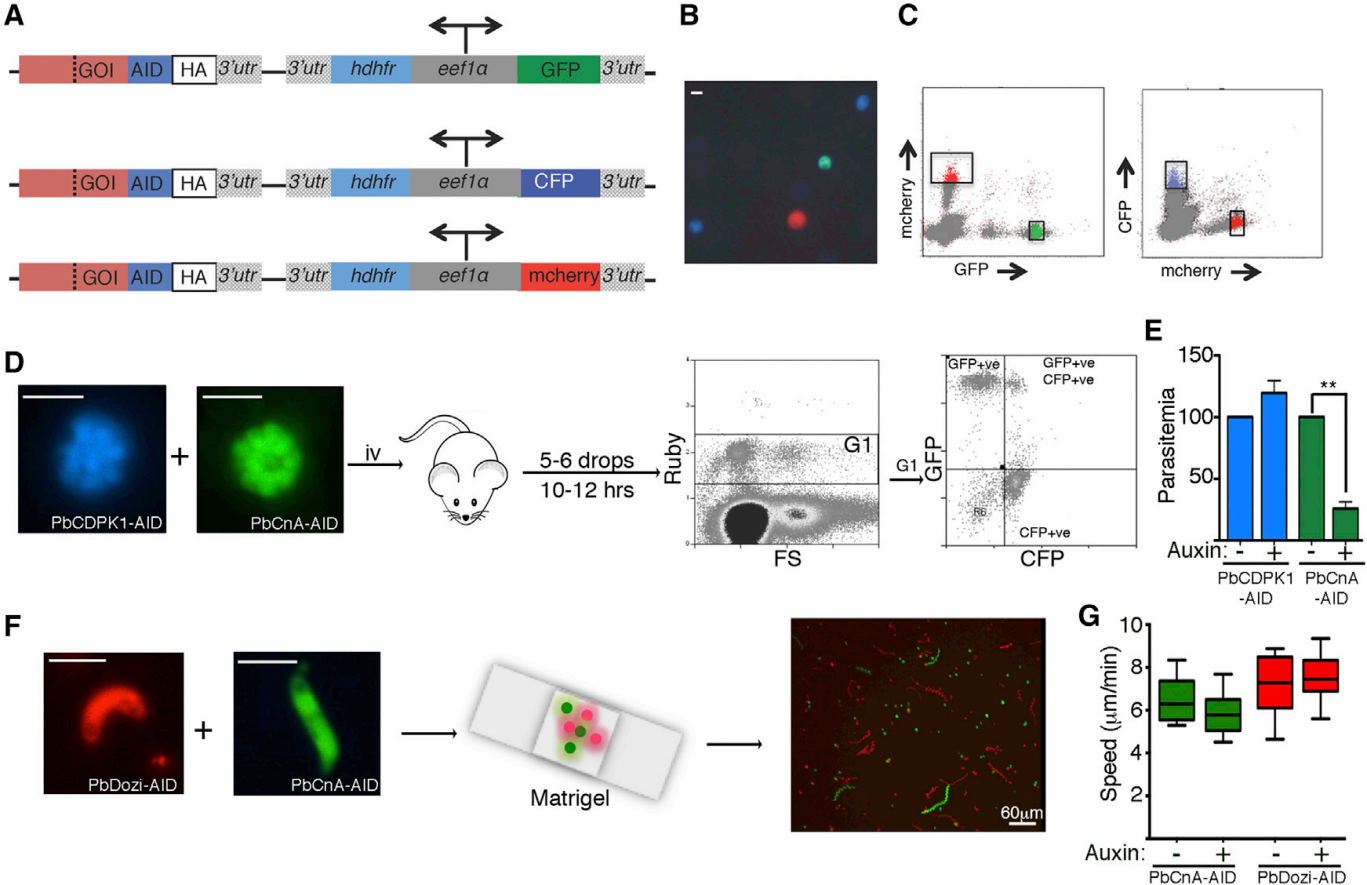
Multiplex Generation and Phenotyping of Transgenic Lines (A) Schematic of vectors generated for simultaneous transfection and generation of multiple transgenic lines. In addition to C-terminally tagging the gene of interest with AID-2xHA, the plasmid expresses the drug selectable marker (*hdhfr*) and a fluorescence marker (*gfp*, *cfp*, or *mcherry*) driven by the bidirectional *pbeef1*α promoter. (B) Fluorescent parasites indicate successful generation of three transgenic lines 8 days post-transfection. (C) Graph of FACS where gates indicate the three types of collected fluorescence-positive cells. Fifty IRBCs of each fluorescence type were intravenously administered to three naive mice to generate GFP-, CFP-, and mCherry-expressing isogenic parasite lines, respectively. (D) Schematic of duplexed in vivo invasion assay where CFP-expressing PbCDPK1-AID schizonts are mixed with GFP-expressing PbCnA-AID line prior to intravenous injection. 15 min later, four to five blood drops are collected by tail prick and cultured for 12 hr followed by flow cytometry analysis. The gated (G) DiCycle ruby positive (infected IRBC) can be further distinguished into either singly or dually fluorescent populations to determine parasitemia. (E) Duplexed in vivo invasion assay where CFP-expressing PbCDPK1-AID and GFP-expressing PbCnA-AID (± auxin) are mixed and intravenously injected (mean ± SEM; two tailed t test for paired observations: p < 0.05; [^∗∗^]). (F) Schematic of duplexed ookinete motility assay where mCherry-expressing PbDozi-AID ookinetes are mixed with GFP expressing PbCnA-AID line in Matrigel. Reconstructed tracks of motile ookinetes with frames collected every 10 s for 15 min (right panel). (G) Ookinete motility (measured by distance covered over time) was measured for mixed red PbDozi-AID and green PbCnA-AID ookinetes (± auxin) (n = 15 instances per condition; bottom and top of box denote first and third quartiles, respectively; whiskers denote minimum and maximum; p = 0.14 for PbCnA-AID line ± auxin and 0.35 for PbDozi-AID line ± auxin). Scale bar denotes 5 μm unless otherwise indicated. See also Figure S5.

In a demonstration of the approach, three AID-tagging plasmids were generated expressing GFP, CFP, or mCHERRY fluorescence (Figure 5A). About 1 kb of C terminus of *pbcna*, *pbcdpk1*, and *pbdozi* were cloned into the plasmids to generate AID-tagged fusions that co-expressed with GFP, CFP, and mCHERRY, respectively (Figure S5A). After linearization all three plasmids were mixed, electroporated, and administered into a single mouse. By 8 days post-transfection, parasites expressing exclusively either GFP, CFP, or mCHERRY were observed (Figure 5B). Parasites were collected, and correct plasmid integration was confirmed by PCR (data not shown). Flow cytometry performed on blood from a mixed infection illustrated the three fluorescence markers were easily distinguishable (Figure 5C). Post-sorting, 50 parasites expressing each fluorescent protein from the indicated gates in Figure 5C were intravenously administered to naive mice. Genetic analysis of the resulting parasites indicated that no wild-type or cross-contamination had occurred in the isolated lines, demonstrating the efficiency and robustness of the isolation procedure (Figures S5B and S5C). Not only do the different fluorescence markers enable generation and isolation of the transgenic lines, they facilitate phenotyping (Figures 5D–5G). We performed a duplexed erythrocyte invasion assay where PbCnA-AID (GFP-expressing) and PbCDPK1-AID (CFP-expressing) schizonts were combined and intravenously injected into mice. While PbCnA-AID depletion resulted in significant reduction in erythrocyte invasion, PbCDPK1-AID demonstrated no observable defects (Figure 5E). Additionally a duplexed ookinete motility assay of PbCnA-AID (GFP expressing) and PbDozi-AID (mCHERRY expressing) indicated neither protein performed essential functions needed for ookinete motility (Figure 5G). Thus, this multiplexed AID system is a significant resource toward scaling up analysis of the parasite phenome, reducing both time for analysis and animal use.

## Discussion

Advances in the mechanistic understanding of biological processes are largely powered by innovations in tools to manipulate the biological system of study. Gene function can be assigned to a biological process by regulating gene expression. However, genetic manipulation in *Plasmodium* is performed in the haploid asexual blood stage, and disruption of genes essential for asexual blood stage development cannot be recovered for functional analysis. Therefore, deducing functions of essential and multifunctional genes at different stages of parasite development requires robust conditional expression technologies to propagate the parasite and scrutinize gene action. The genetically tractable rodent malaria parasite, *P. berghei*, allows both in vitro and in vivo experimental access to its complete life cycle, providing a powerful platform upon which a versatile protein regulation tool can be imposed. Here we developed a chemical-genetic tool that combines targeted gene manipulation techniques and the rapid speed of small molecule action. Advantages of AID technology include the low cost of the auxin ligand in comparison to Shield (for ddFKBP) and overcoming the genetic requirement of human DHFR (for TMP), which is already the most widely used positive selection marker for *P. berghei* genetic modification.

To test if the AID system is indeed a powerful resource for studying multistage-specific and essential gene functions, we analyzed the role of calcineurin. While calcineurin plays pivotal roles in calcium-dependent signal transduction pathways in a variety of eukaryotes, its stage-specific roles in *P. berghei* have remained elusive. Because the AID system was active at both 37°C and 21°C, we were able to examine calcineurin function in the host and mosquito stages of the parasite, including the merozoite, gamete, ookinete, and sporozoite (Figures 1–3). Calcineurin is required for DNA replication in male gametes and is a component of the essential calcium-responsive signaling network unique to *Plasmodium* male gametogenesis (Billker et al., 2004). Furthermore, calcineurin also functions downstream in gamete fertility (Figures 2F and 2G). Gamete formation and zygote-to-ookinete development were previously reported to require calcium-coordinated signaling (Sebastian et al., 2012). The important role of calcineurin in gamete fertility uncovered here further underscores the central nature of calcium involvement in *Plasmodium* biology.

Although targeting different host cell types, the merozoite, ookinete, and sporozoite parasite forms invade and develop by employing conserved molecular mechanisms, the majority of which are coordinated by calcium (Billker et al., 2009). All three invasive forms contain an actin/myosin-driven motor complex, the glideosome, and specialized microneme organelles, which secrete adhesins required for engagement with host cell receptors during parasite invasion and motility (Baum et al., 2008). Our data demonstrating that erythrocyte invasion by the merozoite, mosquito midgut invasion by the ookinete, and hepatocyte invasion by the sporozoite are all regulated in a calcineurin-dependent manner establishes calcineurin as an additional unifying factor for different host cell transitions or colonisations. Moreover, our observations in the ookinete and merozoite as well as studies in the *P. falciparum* merozoite and a related apicomplexan parasite, *Toxoplasma gondi* tachyzoite (Paul et al., 2015, accompanying paper), further delineate the requirement of calcineurin for invasion being independent of microneme exocytosis and parasite motility. Further analysis revealed that calcineurin plays a specific and conserved role in the attachment phase of the merozoite to the target erythrocyte. Therefore, uncoupling calcineurin function from organelle secretion and motility reveals additional complexity in calcium-controlled signaling during host cell invasion.

A significant and validating feature of the AID system when applied to signaling proteins PPKL and CDPK1 was its capacity to replicate previously reported phenotypes produced by genetic manipulation efforts in *P*. *berghei*. Accordingly, our data showed both proteins were dispensable for intraerythrocytic development, but performed critical functions in ookinete development, thereby affecting parasite transmission. Moreover, the AID system could efficiently deplete proteins localized to different cellular compartments. Upon auxin application, cytosolic proteins were robustly degraded as exemplified by depletion of calcinuerin in all examined parasite stages and PPKL in schizonts and gametocytes (Figures 1C, 4A, and 4D). Additionally, the plasma membrane localized CDPK1 in mature schizonts and gametocytes (Green et al., 2008; Sebastian et al., 2012) was susceptible to AID-regulated degradation (Figures 4A and 4D). In mammalian cells, the AID system could efficiently remove nuclear proteins including chromatin-bound ones (Holland et al., 2012). We believe the presence of the proteasome in the parasite nucleus (Oehring et al., 2012) would also allow for AID-mediated depletion of nuclear proteins. Furthermore, detection of a functional 20S proteasome in mature erythrocytes (Neelam et al., 2011) raises the enticing prospect of adapting this technology for parasite proteins exported to the erythrocyte. Recent advancements in gene editing using the CRISPR-Cas9 system and a report implying activity of the AID system in *P. falciparum* (Ghorbal et al., 2014; Kreidenweiss et al., 2013; Wagner et al., 2014) should permit application of the degradation technology to effectively examine endogenous protein function in the human malaria parasite.

We also engineered the degron-tagging plasmid for simultaneous generation of multiple transgenic lines. By using distinct fluorescence markers, we were able to target three different genes in a single transfection experiment and rapidly generate isogenic lines by flow cytometry (Figure 5). The distinct fluorescence markers also improved phenotyping because multiple transgenic or control lines can be used for examining processes that are inherently prone to experimental variation. Improved experimental design by employing multiple fluorescent lines ensures reproducibility that could result in significant reduction in sample size while providing robust statistics. Overall, a remarkable reduction in animal use can be achieved including 85% cut for parasite line generation and at least a further 50% for phenotypic analysis.

By applying and optimizing the AID technology to *P. berghei*, we now have a powerful and specific tool to examine endogenous protein function during multiple stages of parasite development. We showed that calcineurin controls critical parasite developmental switches in both the host and vector, implicating this phosphatase as a promising candidate for therapeutic interventions targeting both parasite development and transmission.

## Experimental Procedures

The Supplemental Experimental Procedures provides a list of all reagents, generation of transgenic lines, and detailed description of all techniques used in this study. Two-tailed t test for paired observations was used for all statistical analyses.

### Ethics Statement

All animal procedures were carried out according to UK Home Office regulations and protocols approved by the University of Glasgow Ethics Committee.

### Invasion Assays

For erythrocyte invasion, purified schizonts were incubated ± 500 μM indole 3-acetic acid (IAA) for 30 min. Merozoites were isolated by filtration and intravenously administered to mice. 15 min post-injection, blood was collected from mice, confirmed ring-stage parasites by microscopy, cultured infected erythrocytes ex vivo for 10 to 15 hr, and measured parasitemia by flow-cytometry. For in vitro attachment/invasion assays, merozoites were incubated with erythrocytes under shaking conditions for 10 min, fixed in 4% PFA, and examined by microscopy. For PbAMA-1 processing assays, purified merozoites were either incubated in buffers mimicking extracellular (high Na^+^, low K^+^) or intracellular (low Na^+^, high K^+^) conditions. Pellet lysates were examined by western blotting to detect the processed ∼20 kDa AMA-1 protein.

For mosquito midgut infection, ookinetes grown (±IAA) were membrane fed to mosquitoes, and midguts were dissected 7 days later for oocyst counts. For HepG2 infection assays 22 day post-transmission, sporozoites were isolated, pre-treated (±IAA), and incubated with HepG2 cells. Intracellular sporozoites and subsequent EEFs were examined 2 and 48 hr post-invasion, respectively.

### Phenotypic Analysis of Sexual-Stage Development

Gametocytes were pre-incubated in schizont media (±IAA) for 30 min at 37°C prior to activation. Cultures were spun down and incubated with ookinete media (±IAA) for further post-activation analysis. Exflagellation assays and ookinete conversion rates were assessed as described (Philip et al., 2012). DNA replication assay and analysis by FACs was performed as described (Laurentino et al., 2011). We assayed gametocyte emergence by measuring rupture of erythrocytes pre-labeled with a fluorescent α-mTER119 antibody by fluorescence microscopy.

### Ookinete Microneme Secretion and Motility Assays

For the microneme secretion assay, fertilized zygotes 4 hr post-gametocyte activation were incubated ± IAA. Ookinetes were purified on a LD50 magnetic column 15 hr later and incubated in PBS ± IAA for an additional 4 hr. The supernatants were examined as previously reported (Philip et al., 2012). Ookinetes for motility assays were handled similarly to the microneme secretion assay. The purified ookinetes (±IAA from 4 to 6 hr post-activation) were embedded in Matrigel. Samples were incubated for 1 hr at 21°C before imaging. Time-lapse movies were acquired every 10 s for 15 min on a Leica M205 FA fluorescence stereomicroscope.

### Flow Cytometric Isolation of Transgenic Parasites

Parasites individually expressing the three fluorescence markers (GFP, CFP, and mCHERRY) were propagated in mice until the parasitemia reached 0.2%–1.0%. IRBCs were suspended in schizont media, and sorting was performed on a BDFACSAria III cell sorter. 1,000 IRBCs for each fluorescence were collected, and 50 cells were intravenously administered into mice. Successful isolation and purification of parasites were confirmed by microscopy and integration PCRs.

For additional details for all assays, see Supplemental Experimental Procedures.

## Author Contributions

N.P. designed and performed experiments, analyzed the data, and wrote the manuscript A.P.W. supervised the study and wrote the manuscript.

## Supplemental Information

Document S1. Figures S1–S5, Table S1, and Supplemental Experimental Procedures

Document S2. Article plus Supplemental Information
