# Supplementary material for: Conditional Degradation of Plasmodium Calcineurin Reveals Functions in Parasite Colonization of both Host and Vector
Source: Cell Host Microbe. 2015 Jul 8;18(1):122–31. doi: 10.1016/j.chom.2015.05.018 (PMC4509507; doi:10.1016/j.chom.2015.05.018)
Supplement: Document S1. Figures S1–S5, Table S1, and Supplemental Experimental Procedures [file mmc1.pdf]

Cell Host & Microbe, Volume *18*

Supplemental Information

Conditional Degradation of *Plasmodium* Calcineurin Reveals Functions in Parasite Colonization of both Host and Vector

Nisha Philip and Andrew P. Waters

Figure S1

A

Gene	P.berghei	P.falciparum
Skp1	PBANKA_114290	3D7_1367000
Cul1	PBANKA_142650	3D7_0811000
Rbx1	PBANKA_080620	3D7_0319100

B

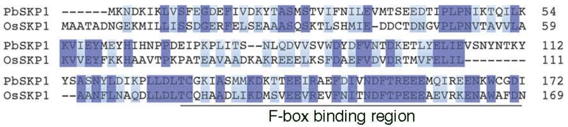

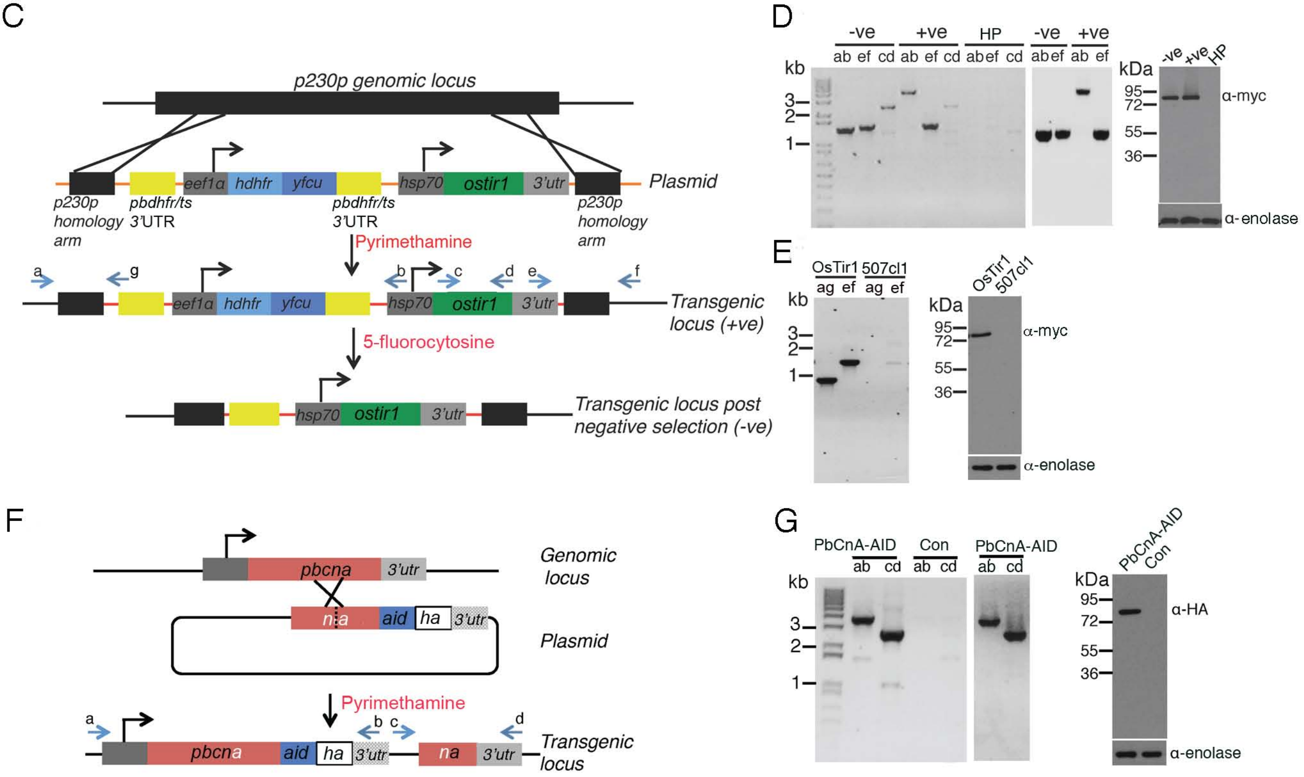

Figure S2

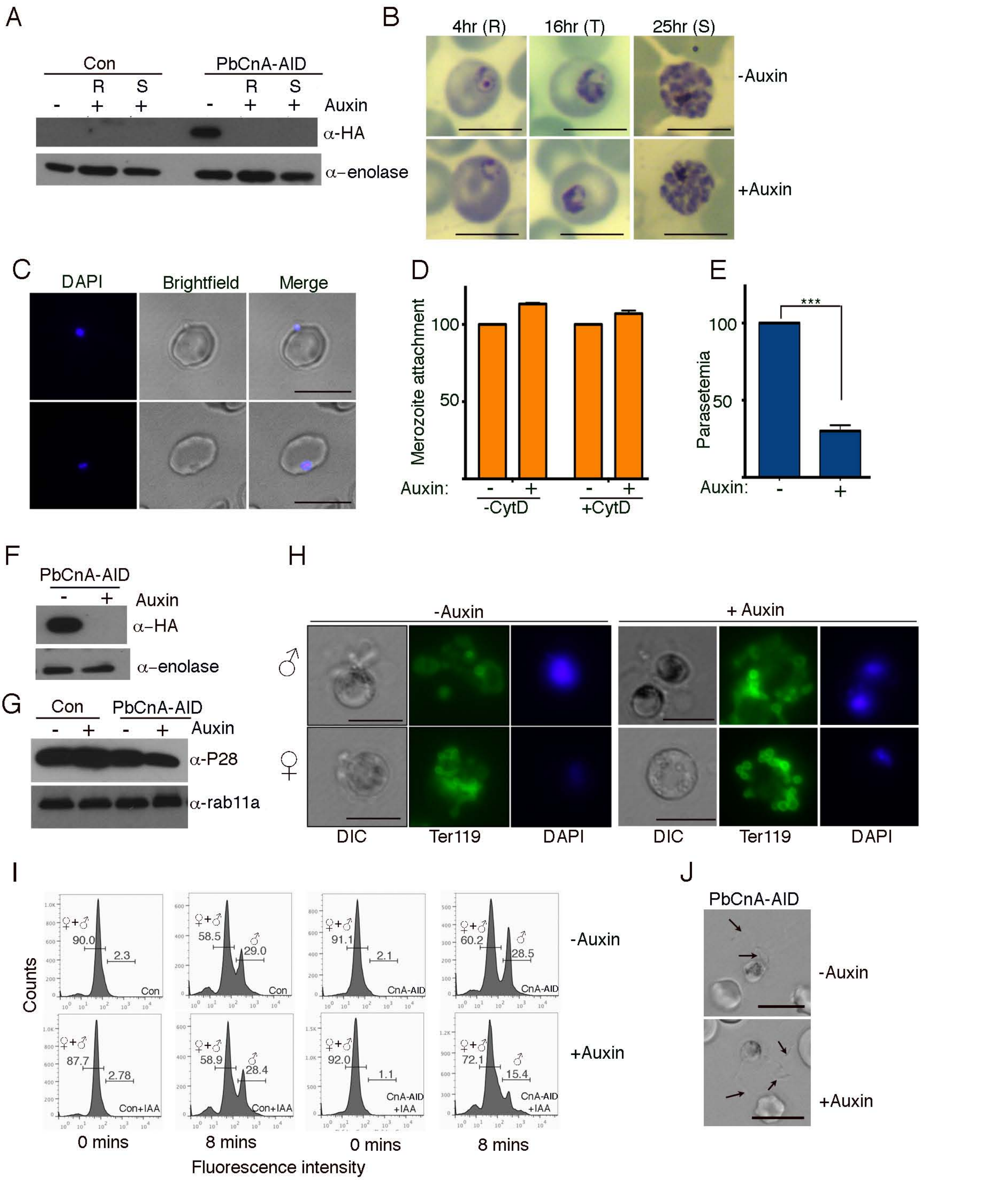
A
Con
PbCnA-AID
R S R S
Auxin
α-HA
α-enolase
B
4hr (R)
16hr (T)
25hr (S)
-Auxin
+Auxin
C
DAPI
Brightfield
Merge
D
Merozoite attachment
Auxin:
-CytD
+CytD
E
Parasetemia
***
Auxin:
F
PbCnA-AID
Auxin
α-HA
α-enolase
G
Con
PbCnA-AID
Auxin
α-P28
α-rab11a
H
-Auxin
+ Auxin
DIC
Ter119
DAPI
I
Counts
-Auxin
+Auxin
0 mins
8 mins
0 mins
8 mins
Fluorescence intensity
J
PbCnA-AID
-Auxin
+Auxin

Figure S3

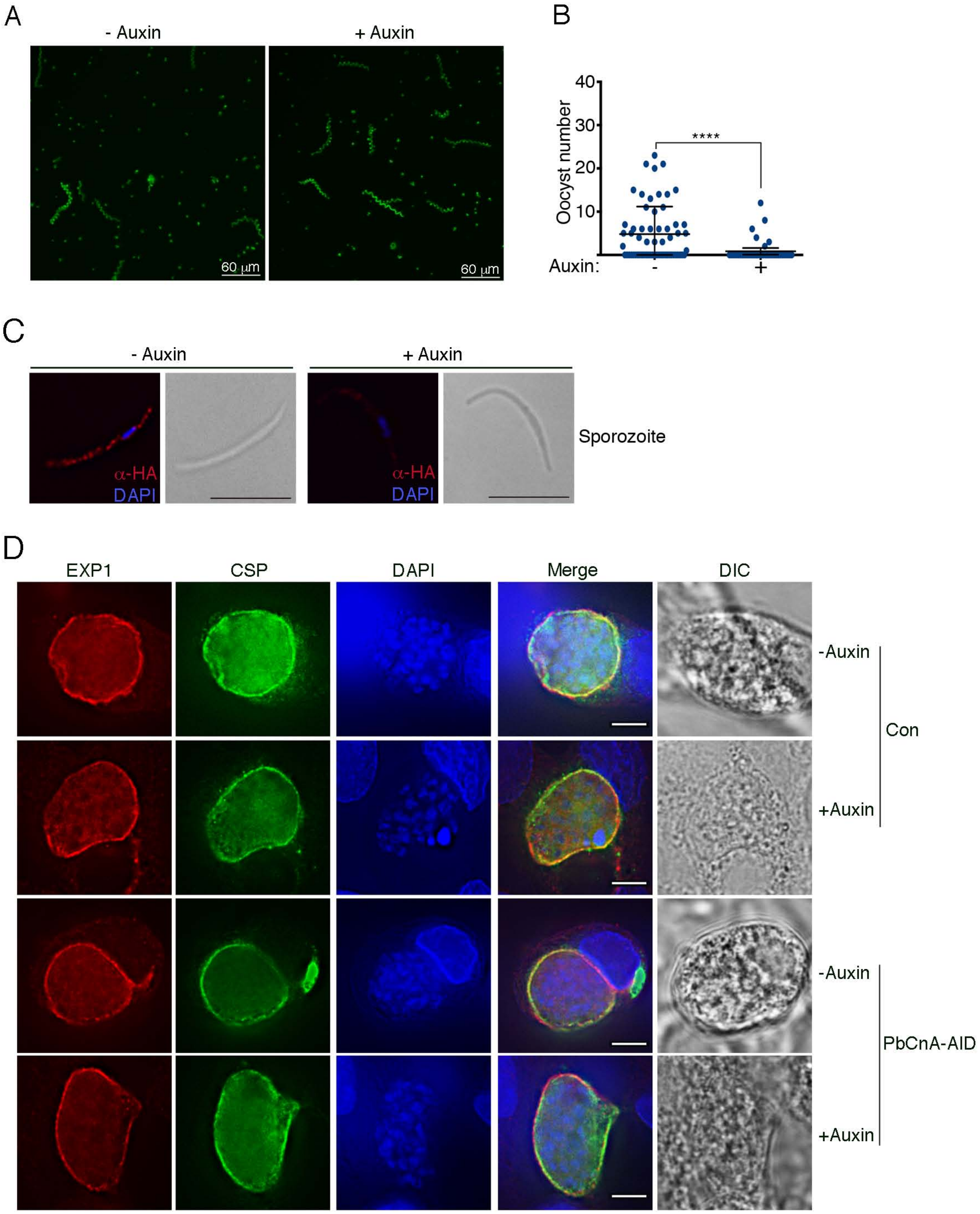
A
- Auxin
+ Auxin
60 μm
60 μm
B
Oocyst number
40
30
20
10
****
Auxin:
-
+
C
- Auxin
+ Auxin
α-HA
DAPI
α-HA
DAPI
Sporozoite
D
EXP1
CSP
DAPI
Merge
DIC
-Auxin
+Auxin
Con
-Auxin
+Auxin
PbCnA-AID

Figure S4

A

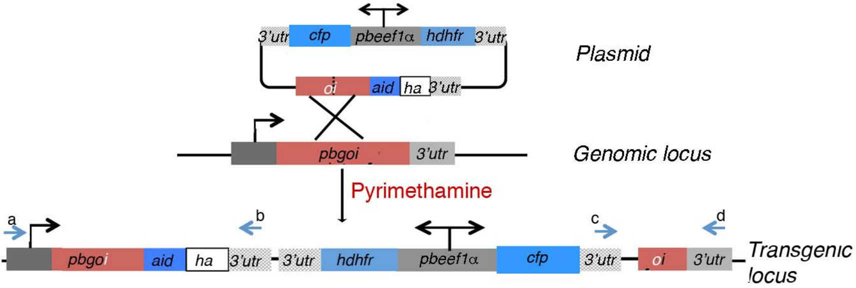

B

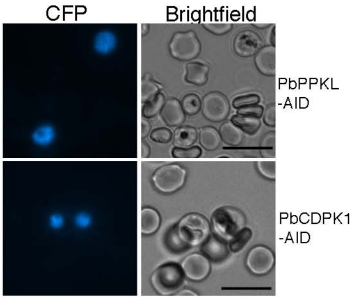

C

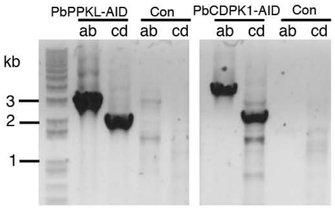

Figure S5

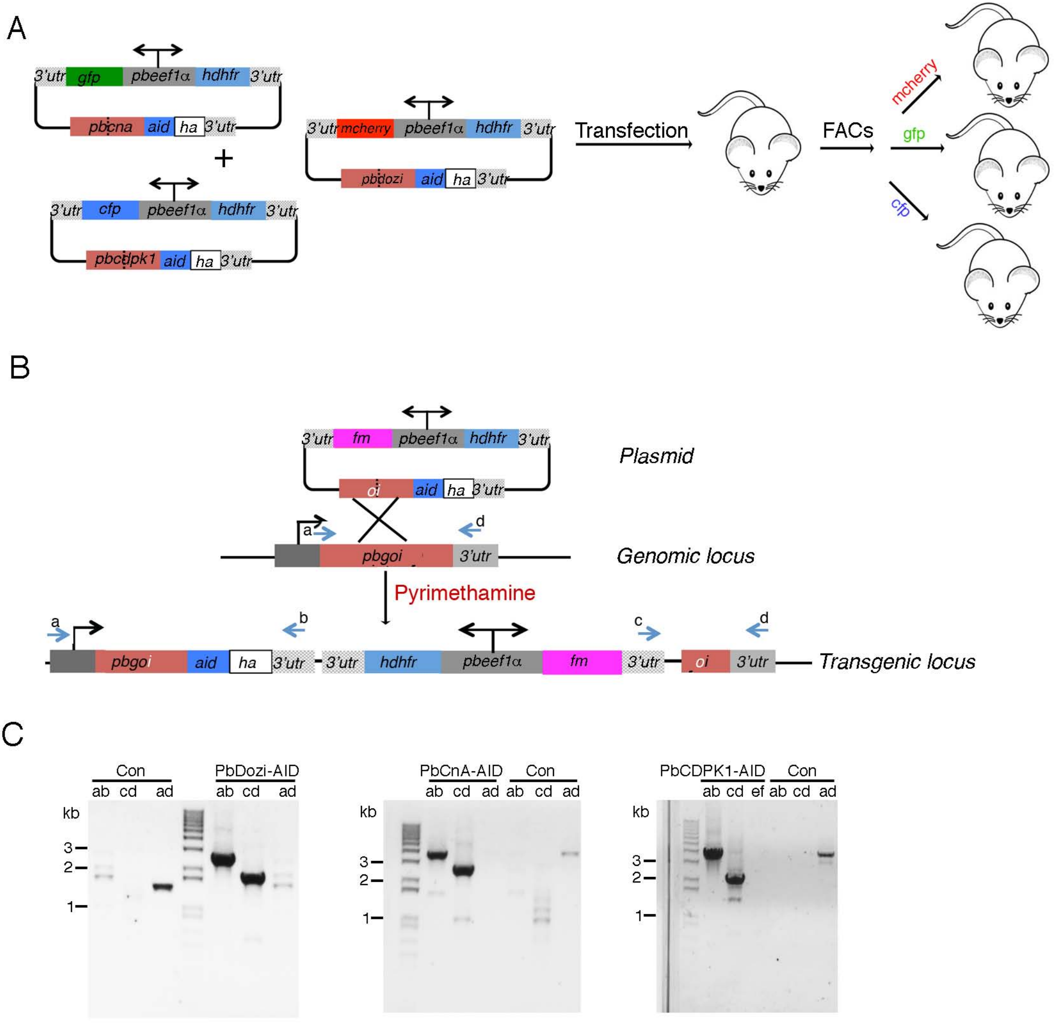

A
3'utr
gfp
pbeef1α
hdhfr
3'utr
pbcna
aid
ha
3'utr
+
3'utr
cfp
pbeef1α
hdhfr
3'utr
pbcdpk1
aid
ha
3'utr
3'utr
mcherry
pbeef1α
hdhfr
3'utr
pbdozi
aid
ha
3'utr
Transfection
FACs
mcherry
gfp
cfp
B
3'utr
fm
pbeef1α
hdhfr
3'utr
Plasmid
oi
aid
ha
3'utr
a
d
pbgoi
3'utr
Genomic locus
Pyrimethamine
a
b
c
d
pbgoi
aid
ha
3'utr
3'utr
hdhfr
pbeef1α
fm
3'utr
oi
3'utr
Transgenic locus
C
Con
PbDozi-AID
ab cd ad
ab cd ad
kb
3
2
1
PbCnA-AID
Con
ab cd ad
ab cd ad
kb
3
2
1
PbCDPK1-AID
Con
ab cd ef
ab cd ad
kb
3
2
1

Supplemental Information

SUPPLEMENTAL FIGURE LEGENDS

Figure S1. Related to Figure 1

(A) Homologs of SCF complex comprising of Skp1 (S-phase associated protein 1), Cullin 1 and Rbx1 (Ring box protein 1) are present in *Plasmodium*. Homologs were initially identified by gene text searches on PlasmoDB, followed by confirmation on NCBI blastp search (http://blast.ncbi.nlm.nih.gov) against the non-redundant protein databases (nr).

(B) Alignment of *P. berghei* and *O. sativa* SKP1 using ClustalW illustrates a 64% identity (dark blue) and 93% homology (light blue) in the F-box binding region.

(C) Strategy for generation of marker-free (-ve) parent acceptor line expressing F-box protein TIR1 under the *hsp70* promoter. The *ostir1* gene expressed under the *hsp70* promoter is targeted to the non-essential *p230p* gene locus by double crossover and transgenic parasites are positively selected by providing pyrimethamine in drinking water to infected mice. Parasites showing successful integration (+ve) were cloned by limiting dilution, followed by negative selection using 5-fluorocytosine. Successful integration of *ostir1* and removal of the drug selection cassette containing *hdhfr* and *yfcu* genes was confirmed by PCR, with indicated primers [5' integration (ab): GU868/GU924); 3' integration (ef): (GU706/GU871 for *p48/45* 3'UTR and GU2050/GU871 for *p28* 3'UTR]; drug selection cassette removal (ab): (GU868/GU924)], and the transgenic line further cloned by limiting dilution to generate the *hdhfr* marker-free parent acceptor line.

(D) Expected PCR product sizes with indicated primers, as in Figure S1C, demonstrate proper integration of *hsp70*-driven *ostir1* plasmid at the *p230p* locus and removal of *hdhfr* and *yfcu* genes (left panel: *ostir1* with *p48/45* 3'UTR (pG230); middle panel: *ostir1* with *p28* 3'UTR (pG402); HP background strain is also shown. Right panel: myc-tagged OsTIR1 protein is expressed in transgenic *P. berghei* parasites (pG230 line is shown) before and after negative

selection. pG230 transfected parent line was used as control for all blood stage phenotyping, which include intraerythrocytic development, gametocyte maturation, activation and fertilization.

(E) PCRs, as in Figure S1C, showing proper integration of *hsp70*-driven *ostir1* (pG402) plasmid at the *p230p* locus in the GFP expressing 507cl1 line [5' integration (ab): GU868/GU869; 3' integration (ef): GU2050/GU871]. This line was used as a control for phenotypic analysis following gamete fertilization (ookinete motility, microneme secretion, sporozoite and EEF assays). Western blotting shows expression of OsTIR1-9myc protein (right panel). Enolase serves as the loading control.

(F) Schematic for single crossover generation of PbCnA-AID parasite strain. The targeting plasmid containing 1 kb C-terminus of *pbcna* gene is linearized by digestion with BsaBI restriction (dotted line) enzyme prior to transfection. Transgenic parasites were selected by pyrimethamine and correct integration checked by PCR with indicated primers [5' integration (ab): GU144/GU730; 3' integration (cd): GU533/GU147].

(G) PCRs, as in Figure S1F show upstream and downstream integration of *pbcna-aid-ha* targeting constructs in both parent lines (pG230, left panel; pG402, middle panel). Western blotting, followed by probing with α-HA antibody confirms expression of ~80 kDa protein corresponding to size of AID-HA-tagged PbCnA protein (pG230 background). Enolase serves as the loading control.

Figure S2. Related to Figure 2

(A) Efficient depletion of PbCnA-AID (α-HA) protein in schizonts when IAA (auxin) was added either to ring stage (R) or mature schizont stage parasites (S). Rings and schizonts were treated with IAA for 24 hrs or 30 min, respectively. Resulting PbCnA-AID protein depletion in these samples was examined by Western blotting. Enolase serves as the loading control.

(B) IAA treatment of ring-stage parasites has no effect on intraerythrocytic development. Ring stage infected blood was collected 15 min post-intravenous administration of mature schizonts.

PbCnA-AID parasites were cultured ± auxin smears made at 4 hr rings (R), 16 hr trophozoites (T) and 25 hr schizonts (S), stained with Giemsa and examined by light microscopy. Scale bar = 10 µm

(C) Representative images of attached merozoites (top panels) or invaded rings (bottom panels) examined 10 minutes post addition of purified merozoites to erythrocytes. Merozoites derived from mature schizonts (± auxin) were added to erythrocytes, supplemented with schizont media to a final hematocrit of 10% and shaken at 700 rpm. A 100µl aliquot was fixed, probed with DAPI and examined by microscopy.

(D) Merozoite attachment and invasion is unaffected in the control line (± auxin). The assay was performed as indicated in Figure 2C where a minimum of 10 fields containing ~350 erythrocytes were counted. 100% is the proportion of attached/invaded parasites without auxin treatment.

(E) Parasitemia resulting from successful invasion of erythrocytes was assessed 4 hours post addition of merozoites to erythrocytes. Experiment was performed as in Figure S2C where after shaking for 10 minutes, cells were placed in schizont media and incubated at 37°C. Four hours later, cultures were smeared and ring stage parasitemia was measured by Giemsa staining and light microscopy. 100% is parasitemia without auxin treatment (n=3 experiments; mean ± SEM; two tailed t test for paired observations: P <0.001 [***]).

(F) Mature gametocytes employed for phenotypic analysis have undetectable PbCnA-AID protein levels upon pre-treatment with IAA. Gametocytes were treated ± auxin for 30 min (at 37°C), collected, lysed, and PbCnA-AID protein levels were determined by Western blotting. Enolase serves as the loading control.

(G) Translation of *p28* and *rab11a* transcripts is unaffected by PbCnA-AID depletion, indicating normal translational control in activated female gametes (Mair et al., 2006). Gametocytes were pre-treated ± auxin for 30 min (at 37°C), activated at 21°C by addition of 100 µM xanthurenic acid in RPMI culture media. Parasite pellets were collected 3 hrs post-activation, lysed and

examined by Western blotting. Rabbit polyclonal antibodies against P28 and Rab11a were used to examine protein levels.

(H) Male (♂) and female (♀) gametocyte emergence upon activation. Infected cells were incubated ± auxin for 30 min, followed by FITC-conjugated α-mouse TER119 ± auxin for an additional 30 min. Cells were activated at 21°C by addition of 100 μM xanthurenic acid in RPMI culture media and emergence observed by DIC and fluorescence microscopy. Nuclei are DAPI stained. Scale bar = 10μm

(I) Reduction in the number of male gametocytes replicating their DNA when PbCnA-AID is depleted. Histogram showing proportion of male gametocytes undergoing DNA replication before and after activation, as in panel F. Prior to activation (0 min), males (♂) and females (♀) show similar DNA content. With activation, only males undergo DNA replication. Here, we assayed DNA replication at 8 min post-activation.

(J) Microgamete formation (arrows) is apparent in the proportion of male gametes undergoing exflagellation when PbCnA-AID is depleted.

Control line (Con) in all experiments is the OsTIR1-expressing line (pG230).

Figure S3. Related to Figure 3

(A) Reconstructed tracks of motile PbCnA-AID ookinetes in matrigel™ ± auxin with frames collected every 10 seconds for 15 min.

(B) Oocyst numbers in mosquito midgut upon PbCnA-AID depletion in mature ookinetes. Auxin was added to parasite cultures six hours post induction of gametogenesis. Mature ookinetes were membrane fed to mosquitoes and seven days later midguts were dissected and GFP-positive oocysts were counted (n= minimum of 40 mosquitoes for each condition; mean ± SD). [****], *P* <0.0001.

(C) Immunofluorescence showing efficient depletion of PbCnA-AID-HA protein in sporozoites upon 500 µM IAA addition. Mouse α-HA antibody was used to detect PbCnA-AID-HA protein. Mouse secondary antibodies conjugated to Alexa 594-red was used. Parasites were fixed 90 min post-treatment with IAA. Brightfield images of the same section are also shown. Scale bar = 10 µm

(D) Representative images of 48 hr EEFs resulting from successful sporozoite invasion and colonization of HepG2 cells. EEFs were probed with α-CSP (circumsporozoite protein) and α-EXP1 (exported protein 1) and nuclear stain DAP1. Scale bar = 5 µm

Control line (Con) in all experiments is the OsTIR1-expressing line (pG402)

Figure S4. Related to Figure 4

(A) Schematic for single crossover strategy of generating of a transgenic parasite line with gene of interest (goi) tagged with *aid-2xha*. The targeting plasmid containing 1 kb C-terminus of goi is linearized by digestion with restriction (dotted line) enzyme prior to transfection. Transgenic parasites were selected by pyrimethamine and correct integration checked by PCR with indicated primers [5' integration (ab); 3' integration (cd)].

(B) CFP expressing parasites were recovered seven days post transfection of plasmid targeting *pbppkl* or *pbcdpk1* gene locus. Bright field images of same field are also shown. Scale bar = 10 µm

(C) PCRs, as in Figure S4A, show upstream and downstream integration of *pbppkl-aid* and *pbcdpk1-aid* targeting constructs. Integration checked by PCR with indicated primers [5' integration (ab): GU151/GU730 for *pbppkl,* GU2900/GU730 for *pbcdpk1*; 3' integration (cd): GU533/GU148 for *pbppkl,* GU533/GU2901 for *pbcdpk1*].

Figure S5. Related to Figure 5

(A) Schematic for generation of three transgenic lines in a single transfection experiment. 2.5 μg each of plasmids targeting *pbcna*, *pbdozi* and *pbcdpk1* loci were mixed with nucleofector™ and electroporated into schizonts. Schizonts were intravenously administered to naïve mice and 24 hours later the mice were given drinking water with Pyrimethamine. 7 days later fluorescent parasites were observed indicating expression of targeting plasmids. CFP, GFP and mCherry expressing Infected red blood cells were purified by fluorescence activated cell sorting and 50 parasites were intravenously administered to three naïve mice.

(B) Schematic for single crossover strategy of generating gene of interest (goi) tagged with AID transgenic parasite line. Transgenic parasites were selected by pyrimethamine and correct integration checked by PCR with indicated primers [5' integration (ab); 3' integration (cd) and non-modified locus contamination determined by (ad)]

(C) PCRs, as in Figure S5B, show upstream and downstream integration of *pbdozi-aid* (left-panel)*, pbcna-aid* (middle-panel) *and pbcdpk1-aid* (right-panel) targeting constructs in parasites lines isolated by FACS. Integration checked by PCR with indicated primers [5' integration (ab): GU730/GU1931 for *pbdozi,* GU144/GU730 for *pbcna,* GU730/GU2900 for *pbcdpk1*; 3' integration (cd): GU533/GU1371 for *pbdozi,* GU533/GU147 for *pbcna* GU533/GU2901 for *pbcdpk1* and unmodified locus contamination (ad): GU2212/GU1371 for *pbdozi,* GU144/GU147 for *pbcna,* GU2900/GU2901 for *pbcdpk1*]

Table S1. Primers used in this study. Related to Supplemental Experimental Procedures

Primer name	Sequence (5' to 3')
GU144	ATGAATTTTACATTTTTTTAAATCTTAATTATGTATTTTAGCATGC
GU147	CTATTTTACTTTACATATATACAACTCAAAATGGTATTG
GU148	TTGGAAATAATTCAAATTAGAGAAAAAAAAATTCATGAGTTT
GU151	GTATATATATTTTTTTTGATTGAATTCCACTATATATTTTTTTGTTC
GU533	GATTAAGTTGGGTAACGCCAG
GU706	ACTAGTCCCGGGCTTAACATTCACATATATTAATAATTTTAAT
GU762	ATGGTCAAATGTATCTATATTATGTTCTATGG
GU730	GTTAAATGTGGGGTAAAAAAGAGT
GU868	GCAGATTATTTTATAAATAATTCATCAACC
GU869	GCTATTTACATGCATGTGCATGC
GU871	CATAAATCGACACACACAGAAATCGCC
GU924	CAAATTCAATGAACTTCTAATATGACTC
GU1371	CAATTATATTTGTGTTCGCATGTTTAATTGGG
GU1931	GGCATAATTCTTTCATCTCAATACGTTGC
GU2050	ATTCCCGGGCAATTGTTATCGCATATTGTAGGAA
GU2212	GTGTCGCTTCAAATGCTAATACCAATG
GU2578	GGTACCTTTTATAAAATTTTTATTTATTTATAAGCAAATATATAT
GU2579	TAATAAAGGGCACTTTAATTT
GU2580	GACCATATAAGAATTAACCCTTTACTTTTTTC
GU2581	AATTATTAATATATATGAATATATATACATCGTTGTATGCC
GU2598	TTTGTATAGTTCATCCATGCC
GU2599	ATGAGTAAAGGAGAAGAACTTTTCAC
GU2603	GACTACAAGACCCGATTACAATAGTTGG
GU2604	TTCATTTGATGGTCTCTTTTCATTTATACTATC
GU2607	GTGTCGCTTCAAATGCTAATACCAATG
GU2608	TAGTATAGATGGATGGATCTATTTCGTTAGGGATC
GU2847	GGTGTAATCAAAGTAAAAGTGCAAATGATG
GU2848	AAATGTTTTATGGTCACAAATTTTGTGC
GU2849	TGGAGCCCCATAATTTAATTCTCTC
GU2900	GTCATCCCTAACTACCCAAG
GU2901	TTCTTTTTCTCTCTTATTTCTATTCCTC

SUPPLEMENTAL EXPERIMENTAL PROCEDURES

Ethics statement for animal experimentation

All infections were performed on Theiler's original (TO) mice (age 6–8 weeks; weight 25–30 g) (Philip et al., 2013) and animal procedures were carried out according to UK Home Office regulations and protocols were approved by the University of Glasgow Ethics Committee (Project licence 60/6443 to A.P.W.).

Generation of transgenic parasites

All transgenic parasite lines were generated by electroporation of gene-targeting plasmids into purified schizonts followed by intravenous administration into mice according to previously established protocols (Philip et al., 2013). *P. berghei* ANKA HP and 507cl1 (GFP expressing under *ee1f*α promoter in the HP background (Janse et al., 2006) lines were obtained from C. Janse at Leiden University Medical Centre (Leiden, Netherlands).

We constructed plasmids using standard molecular biology techniques. All primer sequences are provided in Table S1. The plasmid (pG230) used to generate the OsTIR1 expressing parent line (in *P. berghei* ANKA HP background) was derived from the pG0148 vector (Sinha et al., 2014). The *cfp* gene in pG0148 was replaced by *ostir1-9myc* [amplified from BYP6743 plasmid (Yeast Genetic Resource Center, Osaka, Japan) with primer pair GU2102/GU2063] using the XhoI and SmaI restriction sites between the *hsp70* (PBANKA_071190) promoter and *p45/48* 3′ UTR. The pG230 plasmid contains target regions for double crossover integration into the locus for the non-essential gene *p230p* and also a negative selection cassette to generate a marker-free line (see Figure S4D) (Orr et al., 2012). This line was used as control and an acceptor to generate the *pbcna-aid* line to examine intraerythrocytic development and gametocyte-ookinete transition assays. A second OsTIR1 expressing parent line was generated where the *p45/48* 3'UTR was replaced by the *p28* 3'UTR (pG402), which represses translation of protein in the gametocyte till 90 min post-activation.

Following transfection into *P. berghei*, the pG402-based parent line gave higher ookinete yields and was therefore used as an acceptor to generate *pbcna-aid* lines to examine ookinete secretion and motility assays, and sporozoite invasion assays. Integration of *hsp70-ostir1-p45/48*–at the *p230p* locus was tested at 5' (GU868/GU924) and 3' (GU871/GU706) and negative selection to remove the *hdhfr* selectable marker was confirmed (GU868/GU924). The 3' integration of the TIR1 line with *p28* 3'UTR was also confirmed (GU871/GU2050). The pG402 plasmid was also transfected into the GFP-expressing 507cl1 parasites and integration at the *p230p* locus was confirmed at 5' (GU868/GU869) and 3' (GU871/GU2050) ends. This GFP-fluorescent parasite line was used as control for ookinete motility, microneme secretion, sporozoite and EEF assays.

The *aid* degron tagging plasmid (pG390) was generated by amplification of the degron CDS from BYP6739 (Yeast Genetic Resource Center, Osaka, Japan) using primer pair BamHI-GU2065/BamHI-GU2066, followed by ligation into the PL31-HA plasmid (Philip et al., 2012). A GFP expressing version of the degron plasmid (pG362) was also generated by replacing the drug selection cassette (*tgdhfr* promoter-*tgdhfr*) with the bidirectional *pbee1f*α promoter driving both *gfp* and *hdhfr* expression. The pG362 plasmid was generated as detailed below. The PL0035 vector (Philip et al., 2012) was digested with NheI and re-ligated to remove the *yfcu pbdhfr*- 3′ UTR (pL0035a). Part of *pbee1f*α promoter was amplified by (SacII)-GU2578/(SpeI)-GU2579 from *P. berghei* genomic DNA, digested with SacII and SpeI, and ligated into pL0035a (also digested with SacII/SpeI) resulting in the reconstitution of the complete bidirectional *pbee1f*α promoter driving expression of the *hdhfr* selectable marker (pL0035b). Primer GU2578 also introduced a KpnI site upstream of the SacII site at the 5' end of *pbeef1*α promoter. The 3'UTR from the *P. berghei* gene *cam* (PBANKA_101060) was amplified with (PstI)-GU2580/(KpnI)-GU2581 from genomic DNA, digested with KpnI and PstI and ligated into pL0035b (pL0035c). The *gfp* gene was amplified from pL0031-*pbppkl* vector (Philip et al., 2012)

using (KpnI)-GU2598/(KpnI)-GU2599, digested with KpnI and ligated into pL0035c (also digested with KpnI) between the *cam*-3'UTR and *pbee1f*α promoter. Finally, the cassette was digested with PstI and NheI and ligated into the *aid* degron expressing plasmid pG390, where the drug selection cassette (*tgdhfr* promoter-*tgdhfr*) had been removed with a PstI/NheI digest. This resulted in the *aid-p48/45* 3′ UTR-*pbdhfr* 3′ UTR *-hdhfr- ee1f*α*-gfp-calmodulin* 3′ UTR plasmid (pG362). The two additional fluorescence marker expressing plasmids were generated by replacing the gfp ORF with either cfp (pG363) or mcherry (pG364).

The *pbcna*-*aid*-2x*ha* tagged construct was generated by cloning 1 kb of the *pbcna* 3' end up to, but not including the stop codon, into pG390 and pG362 plasmids, amplified by using primers (SacII)-GU2603 and (BamHI)-GU2604. Correct integration of the targeting plasmid and gene modification was verified by integration PCR (5'- GU144/GU730; 3'-GU533/GU147) and Western blotting. The pG390-*pbcna-aid* transfected into the pG230 based parent line (non-fluorescent), was used for all blood stage phenotyping, which include intraerythrocytic development, gametocyte maturation, activation and fertilization. The pG362-*pbcna-aid* transfected into the pG402-based parent line (GFP-fluorescent) was used for phenotyping post-fertilization stages, which include ookinete motility, microneme secretion, sporozoite and EEF assays.

Similarly *pbppkl*, *pbcdpk1* and *pbdozi* tagged with *aid*-2x*ha* constructs were generated by cloning 1 kb of the gene 3' end up to, but not including the stop codon into pG363 (for *pbppkl* and *pbcdpk1*) and pG364 (for *pbdozi*) amplified by using (SacII)-GU2847/(XhoI)-GU2848 for *cdpk1*; (SacII)-GU762/(XhoI)-GU2849 for *ppkl*; (SacII)-GU2607/(NotI)-GU2608 for *dozi*. All plasmids were transfected into the pG230 based parent line expressing OsTIR1 controlled by the hsp70 promoter and pb48/45 3'utr. Correct integration of the targeting plasmid was confirmed by integration PCR (5' integration: GU730/GU1931 for *pbdozi,* GU144/GU730 for *pbcna,* GU730/GU2900 for *pbcdpk1* ; 3' integration: GU533/GU1371 for *pbdozi,* GU533/GU147

for *pbcna,* GU533/GU2901 for *pbcdpk1* and unmodified locus contamination : GU2212/GU1371 for *pbdozi,* GU533/GU147 for *pbcna,* GU2900/GU2901 for *pbcdpk1*)

Protein depletion assay

To determine degradation kinetics of PbCnA-AID protein, mature schizonts (enriched on a 55 % Nycodenz gradient), gametocytes (isolated on a LD-50 MACs column, Miltenyi) or salivary gland sporozoites were incubated with 500 μM Indole 3-acetic acid (IAA) dissolved in schizont media (RPMI1640 containing 25 mM HEPES, 5 mM hypoxanthine, 20% FCS, 10 mM sodium bicarbonate, 100 U/ml penicillin and 100 μg/ml streptomycin) for indicated periods of time. The cells were either immediately snap frozen in liquid nitrogen for Western blot analysis or fixed with 4% EM grade paraformaldehyde (in PBS) and smeared onto glass slides for immunofluorescence assays. To test if protein depletion is dependent on the proteasome, parasites were pre-incubated with 1 μM MG132 (Sigma) for 1 hr prior to treatment with IAA. IAA concentration for all assays performed in this study is 500 μM.

Parasite developmental assays

To determine the effect of PbCnA protein depletion on schizont development, purified schizonts were intravenously administered to mice, followed by isolation of rings by cardiac puncture 30 min later. The parasites were cultured in schizont media for 25 hrs ± IAA and schizont development and merozoite count determined by Giemsa smears. To assess gametocytemia, purified schizonts were intravenously administered to mice, followed by isolation of rings by cardiac puncture 30 min later. Blood was passaged through an LD-50 MACs column to remove mature gametocytes. The flow-through was cultured for 36 hrs ± IAA and gametocytemia was determined by Giemsa smears.

Western blotting and immunofluorescence assays

Parasite pellets were lysed in RIPA buffer (50 mM Tris-HCl, pH 7.5, 150 mM NaCl, 2 mM EDTA, 1% NP-40, 0.1% SDS) supplemented with protease inhibitor (Roche) and clarified lysates were suspended in Laemmli sample buffer and separated on a 10% SDS-polyacrylamide gel. Samples were transferred to nitrocellulose membranes (GE Healthcare) and probed with mouse α-HA (1:1000 in 5% milk/PBS), followed by goat anti-mouse secondary antibody (1:5000 in 5% milk/PBS), and visualized with ECL kit (Pierce) or ECL Advance kit (GE healthcare). Other antibody dilutions were as follows: α-enolase (peptide: KTYDLDFKTPNNDK, rabbit polyclonal at 1:1000), α-chitinase (peptide: HTEKQYKSLSHVDALC, rabbit polyclonal at 1:4000), α-CTRP (peptide: LNGGETPHNSNMEFENVENNDGIIEEENEDFEVIDANDPMW, rabbit polyclonal at 1:4000), α-p28 (peptide: VSKPQAPGTGSETP rabbit polyclonal at 1:4000), α-rab11a (peptide: HTEKQYKSLSHVDALC, rabbit polyclonal at 1:1000), α-WARP (peptide: CNKNNPSSLTSERKTTIKN, rabbit polyclonal at 1:4000) and α-GFP (mouse monoclonal 1:4000, Roche Diagnostics). All rabbit polyclonal antibodies were generated at Proteintech group (Chicago, USA).

For IFAs, fixed parasites were smeared onto glass slides and processed as previously shown (Philip et al., 2012). Primary antibody dilutions were as follows: mouse α-HA antibody (1:400, Sigma), mouse α-tubulin (1:1000), mouse α-P25-Cy5 (1:1000). Secondary antibodies (Alexa fluor 594 or 488, Molecular Probes) were used at 1:2000.

Erythrocyte invasion assays

For erythrocyte invasion assays, nycodenz-enriched mature schizonts were incubated ± IAA for 30 min. The schizonts were ruptured by serially passing the cells through a 5 μm (Acrodisc) and a 1.6 μm (Puradisc, Whatman) to release merozoites. For *in vivo* invasion assays merozoites were subsequently intravenously injected into mice. 5-6 tail blood drops were collected 15 min

post-injection and incubated in schizont media without IAA for 16 hrs. Cells were stained with 2 μM Hoescht in rich PBS (20 mM HEPES, 20 mM glucose, 4 mM $NaHCO_3$, 0.1% BSA) for 30 min at 37°C. Cells were pelleted and resuspended in 5ml FACS buffer (2 mM HEPES, 2 mM glucose, 0.4 mM $NaHCO_3$, 0.01% BSA, 2.5 mM EDTA) and analysed on a CyAn ADP 9 colour flow cytometer (Beckman Coulter) equipped with 405-nm, 488-nm and 642-nm solid-state lasers. Data was analysed on FlowJo software to determine parasitemias. For duplexed invasion assays PbCnA-AID (expressing GFP) and PbCDPK1-AID (expressing CFP) schizonts were combined and incubated ± IAA for 30 min and subsequently intravenously injected into mice. Further analysis was performed as described above for *in vivo* invasion assay, except cells were stained with Vybrant® DyeCycle™ Ruby DNA dye (Life technologies) in rich PBS.

For *in vitro* attachment/invasion assays purified merozoites (± IAA;) were added to mouse erythrocytes supplemented with schizont media (10 % hematocrit; 500μl final volume), ± Cytochalisin D at 1μM and incubated at 37°C with vigorous shaking (700 rpm) for 10 minutes. A 100μl aliquot was fixed in 1.4 ml of 2 % PFA and 0.015 % EM grade glutaraldehyde for 30 min at room temperature. Cells were washed 3X with PBS and smeared on glass slides, mounted with Vectashield™ + DAP1 and examined by fluorescence and light microscopy. A minimum of 10 fields with ~ 350 cells were inspected to determine attached/invaded parasites. To determine parasitemia of successfully invaded parasites, 200 μl of the remaining cells post-shaking were placed in schizont media and allowed to develop for 4 hours. Ring stage parasitemia was calculated from smears by Giemsa staining and light microscopy.

To determine PbAMA1 processing, purified merozoites from schizonts (± IAA) were obtained by mechanical rupture as described for the invasion assay. Assay was performed with minor modifications of a previously described protocol (Singh et al., 2014). Merozoites from the PbCnA-AID expressing line were suspended in buffer mimicking intracellular conditions (IC: 5 mM NaCl, 140 mM KCl, 2 mM EGTA, 1 mM MgCl2, 5.6 mM glucose and 25 mM Hepes, pH

7.2). Sample was split, spun at 3300 g and resuspended in 100μl of either IC or extracellular buffer (EC: 140 mM NaCl, 5 mM KCl, 1 mM CaCl2, 1 mM MgCl2, 5.6 mM glucose and 25 mM Hepes, pH 7.2) for 10 min at 37°C. Cells were spun again at 3300 g and supernatant and pellet were separated, and heated in Laemmli sample buffer for 15 minutes at 75°C. Lysates were separated on a 4-20 % gradient gel and PbAMA1 protein levels and processing was examined by Western blot. Blots were probed with rat mAb28G2 (Narum and Thomas, 1994) at 1:4000 dilution and goat anti-rat secondary (Life technologies).

Sporozoite invasion assays

For sporozoite-HepG2 infection assays, the GFP-expressing PbCnA-AID parasite line was exploited. Sporozoites were isolated 22 days post-transmission from infected mosquito salivary glands. Mosquitoes were sterilized in 70% ethanol followed by a sterile PBS wash. Salivary glands (SGs) were dissected in RPMI supplemented with 100 U/ml penicillin and 100 μg/ml streptomycin and kept on ice. SGs were ruptured by 25 pestle strokes in at 1.5ml Eppendorf centrifuge tube, debris pelleted at 100 x g. The supernatant fraction containing the sporozoites was isolated and examined under a hemocytometer. 1×10^4 sporozoites were treated ± IAA (in schizont media) for 90 min at 21°C and then incubated with HepG2 hepatocyte cells (plated at ~ 70% and 40% confluency for sporozoite invasion and EEF development respectively) in chamber of a 4-well Nunc™Labtek™ slide (Thermoscientific™).

Sporozoite invasion was examined as previously described (Sinnis et al., 2013). 2 hours after addition of sporozoites to HepG2 cells, media was removed and cells fixed in 4% PFA in PBS for 1 hr at room temperature. Samples were blocked in 2 % BSA/PBS for 1 hr at 37°C and incubated with α-PbCSP (mAb3D11) at 1:4000 dilution (in 2 % BSA/PBS) overnight at 4°C. After 3X washes with PBS, cells were incubated with goat α-rat secondary conjugated to Alexa 488. Following 3X PBS washes, cells were permeabilized with ice cold methanol for 15 minutes,

blocked in in 2 % BSA/PBS for 1 hr at 37°C and incubated with α-PbCSP (mAb3D11) at 1:500 dilution for 1 hr at 37°C. Cells were washed 3X in PBS, incubated with goat α-rat secondary conjugated to Alexa 594. Following 3X PBS washes samples were mounted in Vectashield™ + DAPI. Sporozoites were visualized on Leica M205 FA fluorescence stereomicroscope. Extracellular sporozoites appear red while green represents both extracellular and intracellular sporozoites. Proportion of invaded sporozoites were calculated as (# Green sporozoites – # Red sporozoites)/ # Green sporozoites. A minimum of 6 fields per experimental condition was examined.

To assess EEF development, sporozoites were also handled as above. 90 min after addition of sporozoites to HepG2 hepatocytes,cells were washed 3x with 1 ml DMEM (containing 2 mM L–glutamine,10% fetal bovine serum and 2% penicillin/streptomycin) and incubated with DMEM supplemented ± IAA. Media was replenished 1 and 2 days post infection, and EEFs (GFP expressing) were counted on a DeltaVision Epifluorescence microscope (Applied Precision) under a 20x objective. Additionally 48 hr EEFs were fixed in 4% PFA in PBS for 1 hr at room temperature and permeabilized with ice cold methanol at room temperature for 15 min. Samples were blocked in 2 % BSA/PBS for 1 hr at 37°C and incubated with α-PbCSP (mAb) at 1:4000 dilution and α-PbEXP1 (at 1:500 dilution) (van de Sand et al., 2005) overnight at 4°C. Cells were washed 3X in PBS, incubated with goat α-rat secondary conjugated to Alexa 488 and goat α-chicken secondary conjugated to Alexa 594 for 1 hr at 37°C. Following 3X PBS washes samples were mounted in Vectashield™ + DAPI. EEFs were examined on a DeltaVision Epifluorescence microscope (Applied Precision) under a 100X objective. Images were acquired, recorded and deconvulated using softWoRx (Applied Precision).

Phenotypic analysis of sexual stage development

Infections were performed by intraperitoneal administration of cryopreserved, cloned parasites into phenylhydrazine (1.25 mg)-treated mice. Enriched gametocytes were obtained by sulfadiazine (35 ml/l) treating the infected mice for 48 hrs (Beetsma et al., 1998). Gametocytes were pre-incubated in schizont media (with IAA) for 30 min at 37°C prior to activation. Cultures were then incubated with ookinete media (RPMI1640 containing 25 mM HEPES, 5 mM hypoxanthine, 20% FCS, 10 mM sodium bicarbonate, 100 μM xanthurenic acid at pH 7.6; ±IAA) for further analysis. DNA replication, exflagellation assays and ookinete conversion rates were assessed as previously described (Laurentino et al., 2011; Philip et al., 2012). For DNA replication assay, gametocytes were purified on LD-50 MACs column, pre-incubated with IAA and transferred to ookinete media for activation. At 0 and 8 min following addition of ookinete media, cells were fixed in 0.25% glutaraldehyde/PBS solution and stained with 2 μM Hoechst-33258 and DNA content was analysed by FACs on CyAn ADP 9 colour flow cytometer. For gametocyte emergence assay, gametocytes were pre-incubated ± IAA (45 min) with α-mTER119-FITC (1:1000 dilution, 30 min), followed by transfer to ookinete media. Images were acquired in the GFP and brightfield channels (over 5-15 min post-activation) to determine gamete emergence. 100 gametocytes were counted per condition.

Motility and midgut invasion in ookinetes

Ookinetes embedded in Matrigel (BD Biosciences) were incubated for 1 hr at 21° C before imaging. Time-lapse movies were acquired every 10 seconds for 15 min. For the duplexed motility assays time-lapse movies were acquired every 10 seconds for 10 min employing the GFP and mCHERRY filter sets (0.5 second exposure for each). Ookinete speeds were calculated on Fiji software using the MtrackJ plugin (Meijering et al., 2012).

For ookinete feed assays in the mosquito purified ookinetes were produced and handled as described in main experimental procedures. Equal amounts of purified ookinetes (treated ± IAA

from 4-6 hrs post-activation) were mixed with 750 μl of mouse blood and 500 μl ookinete media. The ookinete and blood mixture were fed to mosquitoes via a membrane feeder (maintained at 37°C) for 20 minutes. Midguts were dissected 7 days post-feeding and oocyst numbers were counted.

Flow cytometric isolation of transgenic parasites

After successful integration of all three plasmids was confirmed by PCR, parasites individually expressing the 3 fluorescent markers (GFP, CFP and mCHERRY) were propagated in a mouse until parasitemia reached 0.2-1.0%. Infected blood was examined under the fluorescence microscope to exclude multiply infected erythrocytes. One drop of tail blood was added to 2 ml schizont media, pelleted and resuspended in 5 ml FACs buffer, filtered through a BD FACs tube with cell strainer cap to prevent cell aggregation. Cell sorting was performed on a BDFACSAria III cell sorter (4 lasers: Blue 488nm, Yellow/Green 631nm, Red 640nm and Violet 405nm). Cells were sorted through a 70 micron nozzle at 70 psi and 10,000 events/second. Forward and side scatter gating was initially used to exclude both small cells (platelets or ruptured parasites) and leukocytes. Fluorescent parasites were detected using the respective filter sets: mCherry at 610/20 BP filter and 600 LB mirror; GFP at 530/30 BP filter and 502 LP mirror; CFP at 450/40 BP filter and collected into Eppendorf tubes with RPMI media. 1000 IRBCs for each fluorescence were collected in RPMI and 50 cells were intravenously injected into a mouse. 5-7 days later parasites were recovered and used for further analysis.

SUPPLEMENTAL REFERENCES

Beetsma, A.L., van de Wiel, T.J., Sauerwein, R.W., and Eling, W.M. (1998). Plasmodium berghei ANKA: purification of large numbers of infectious gametocytes. Experimental Parasitology *88*, 69–72.

Janse, C.J., Franke-Fayard, B., Mair, G.R., Ramesar, J., Thiel, C., Engelmann, S., Matuschewski, K., van Gemert, G.-J., Sauerwein, R.W., and Waters, A.P. (2006). High efficiency transfection of Plasmodium berghei facilitates novel selection procedures. Molecular and Biochemical Parasitology *145*, 60–70.

Mair, G.R., Braks, J.A.M., Garver, L.S., Wiegant, J.C.A.G., Hall, N., Dirks, R.W., Khan, S.M., Dimopoulos, G., Janse, C.J., and Waters, A.P. (2006). Regulation of sexual development of Plasmodium by translational repression. Science *313*, 667–669.

Meijering, E., Dzyubachyk, O., and Smal, I. (2012). Methods for cell and particle tracking. Meth. Enzymol. *504*, 183–200.

Narum, D.L., and Thomas, A.W. (1994). Differential localization of full-length and processed forms of PF83/AMA-1 an apical membrane antigen of Plasmodium falciparum merozoites. Mol Biochem Parasitol *67*, 59-68.

Orr, R.Y., Philip, N., and Waters, A.P. (2012). Improved negative selection protocol for Plasmodium berghei in the rodent malarial model. Malar J *11*, 103.

Philip, N., Orr, R., and Waters, A.P. (2013). Transfection of rodent malaria parasites. Methods Mol. Biol. *923*, 99–125.

Sinha, A., Hughes, K.R., Modrzynska, K.K., Otto, T.D., Pfander, C., Dickens, N.J., Religa, A.A., Bushell, E., Graham, A.L., Cameron, R., et al. (2014). A cascade of DNA-binding proteins for sexual commitment and development in Plasmodium. Nature *507*, 253–257.

van de Sand, C., Horstmann, S., Schmidt, A., Sturm, A., Bolte, S., Krueger, A., Lutgehetmann, M., Pollok, J.M., Libert, C., and Heussler, V.T. (2005). The liver stage of Plasmodium berghei inhibits host cell apoptosis. Mol Microbiol *58*, 731-742.
