# Supplementary material for: Conditional Degradation of Plasmodium Calcineurin Reveals Functions in Parasite Colonization of both Host and Vector
Source: Cell Host Microbe. 2015 Jul 8;18(1):122–31. doi: 10.1016/j.chom.2015.05.018 (PMC4509507; doi:10.1016/j.chom.2015.05.018)
Supplement: Document S2. Article plus Supplemental Information [file mmc2.pdf]

Resource

Cell Host & Microbe

Conditional Degradation of *Plasmodium* Calcineurin Reveals Functions in Parasite Colonization of both Host and Vector

Graphical Abstract

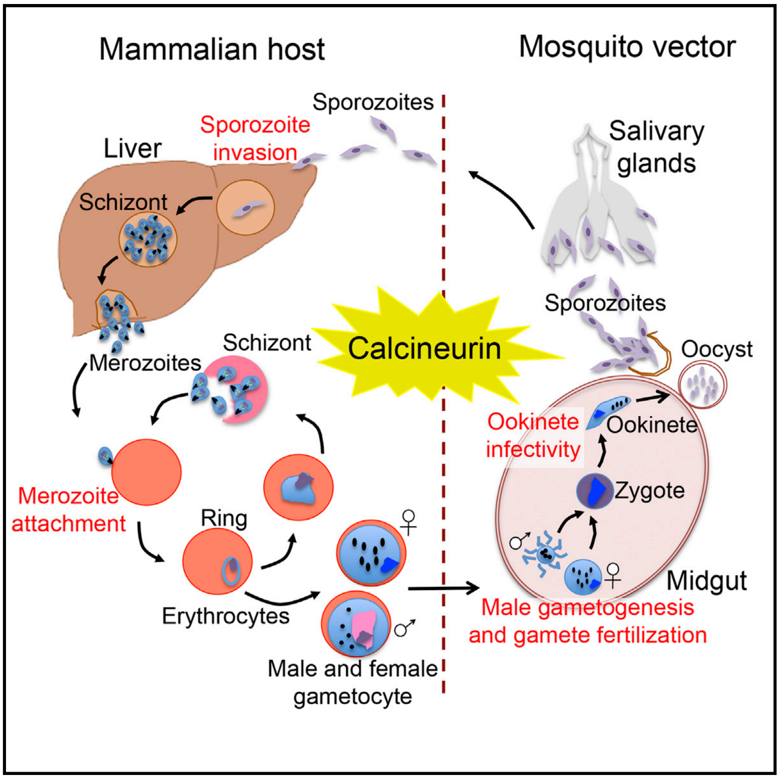

Authors

Nisha Philip, Andrew P. Waters

Correspondence

Nisha.Philip@glasgow.ac.uk (N.P.),
Andy.Waters@glasgow.ac.uk (A.P.W.)

In Brief

Limited conditional regulation technologies in the malarial parasite, Plasmodium, hinder functional examination of parasite proteins operating at multiple stages. Philip and Waters report the application of a rapid and specific conditional protein degradation tool to study parasite Calcineurin function in both host and vector stages of the parasite life cycle.

Highlights

- Calcineurin regulates colonization of host cells across the Plasmodium life cycle
- Calcineurin regulates male gametogenesis
- AID technology is broadly applicable to study protein function in Plasmodium
- Multiplexing of AID technology results in substantially reduced animal use

Philip & Waters, 2015, Cell Host & Microbe *18*, 122–131
July 8, 2015 ©2015 The Authors
http://dx.doi.org/10.1016/j.chom.2015.05.018

CrossMark
CellPress

Cell Host & Microbe
Resource

Conditional Degradation of *Plasmodium* Calcineurin Reveals Functions in Parasite Colonization of both Host and Vector

Nisha Philip[1,*] and Andrew P. Waters[1,*]
[1]Wellcome Trust Centre for Molecular Parasitology, Institute of Infection, Immunity and Inflammation, College of Medical Veterinary & Life Sciences, University of Glasgow, Glasgow, G12 8TA, UK
*Correspondence: Nisha.Philip@glasgow.ac.uk (N.P.), Andy.Waters@glasgow.ac.uk (A.P.W.)
http://dx.doi.org/10.1016/j.chom.2015.05.018
This is an open access article under the CC BY license (http://creativecommons.org/licenses/by/4.0/).

SUMMARY

Functional analysis of essential genes in the malarial parasite, *Plasmodium*, is hindered by lack of efficient strategies for conditional protein regulation. We report the development of a rapid, specific, and inducible chemical-genetic tool in the rodent malaria parasite, *P. berghei*, in which endogenous proteins engineered to contain the auxin-inducible degron (AID) are selectively degraded upon adding auxin. Application of AID to the calcium-regulated protein phosphatase, calcineurin, revealed functions in host and vector stages of parasite development. Whereas depletion of calcineurin in late-stage schizonts demonstrated its critical role in erythrocyte attachment and invasion in vivo, stage-specific depletion uncovered roles in gamete development, fertilization, and ookinete-to-oocyst and sporozoite-to-liver stage transitions. Furthermore, AID technology facilitated concurrent generation and phenotyping of transgenic lines, allowing multiple lines to be assessed simultaneously with significant reductions in animal use. This study highlights the broad applicability of AID for functional analysis of proteins across the *Plasmodium* life cycle.

INTRODUCTION

While completing its complex life cycle, *Plasmodium*, the causative agent of malaria, experiences diverse host environments and undergoes remarkable variation in shape, size, and motility. Parasite development and response to environmental cues are controlled by signaling cascades, many of which are regulated by calcium (Billker et al., 2009). Although calcium is a ubiquitous intracellular messenger in numerous organisms, the malarial parasite maintains both conserved and evolutionarily unique calcium effectors modulating protein phosphorylation. One class of unique effectors are the calcium-dependent protein kinases (CDPKs), a family of protein kinases characteristic of plants and alveolates (Harmon et al., 2000). CDPKs translate calcium signals into diverse outputs, including translational control, microneme secretion, schizont egress, ookinete motility, and liver stage invasion (Bansal et al., 2013; Dvorin et al., 2010; Ishino et al., 2006; Sebastian et al., 2012; Siden-Kiamos et al., 2006).

In addition to CDPKs, fine-tuning of these calcium-dependent processes is thought to require protein phosphatases. In contrast to six or seven CDPKs (depending on species), only two *Plasmodium* phosphatases, protein phosphatase 7 and calcineurin, have calcium-binding motifs, suggesting they modulate several calcium-dependent biological processes (Wilkes and Doerig, 2008). Calcineurin is a heterodimeric protein comprising of a catalytic (CnA) and calcium-binding regulatory (CnB) subunit (Rusnak and Mertz, 2000). Gene expression of both subunits is observed at specific life cycle points in both host and vector stages of the parasite, strongly suggesting a multistage functional profile for this phosphatase and a role in parasite transitions through the life cycle (Otto et al., 2014).

The difficulty in studying multifunctional proteins and proteins essential for intraerythrocytic development in *Plasmodium* is the lack of robust conditional knockout strategies. Although conditional gene deletion was demonstrated in both *P. falciparum* and *P. berghei*, gene removal is irreversible and examines the function of the corresponding protein only at its first point of action (Collins et al., 2013; Combe et al., 2009). Methodologies that inducibly regulate transcription have also been described, but these suffer from slow onset kinetics and have been tested only for asexual stage development (Meissner et al., 2005; Pino et al., 2012). Recently, an inducible protein-RNA-based interaction system was reported where the authors show a robust 80% reduction in protein expression (Goldfless et al., 2014). Although this system will have broad implications in examining protein function in various subcellular contexts, the technique nevertheless has to contend with stability of the protein, which was synthesized prior to imposing regulation. By contrast, direct manipulation of protein levels by chemical-genetic methods can offer significant advantages of inducibility, speed, and specificity. Two such regulatory systems have been developed in *P. falciparum* to regulate protein stability either using FK506-binding protein or a dihydrofolate reductase destabilization domain (Armstrong and Goldberg, 2007; Muralidharan et al., 2011). Unfortunately, these drug-on methods for inducible stabilization require constant application of the small molecule when generating and maintaining transgenic lines and therefore are frequently unsuitable for use in in vivo settings.

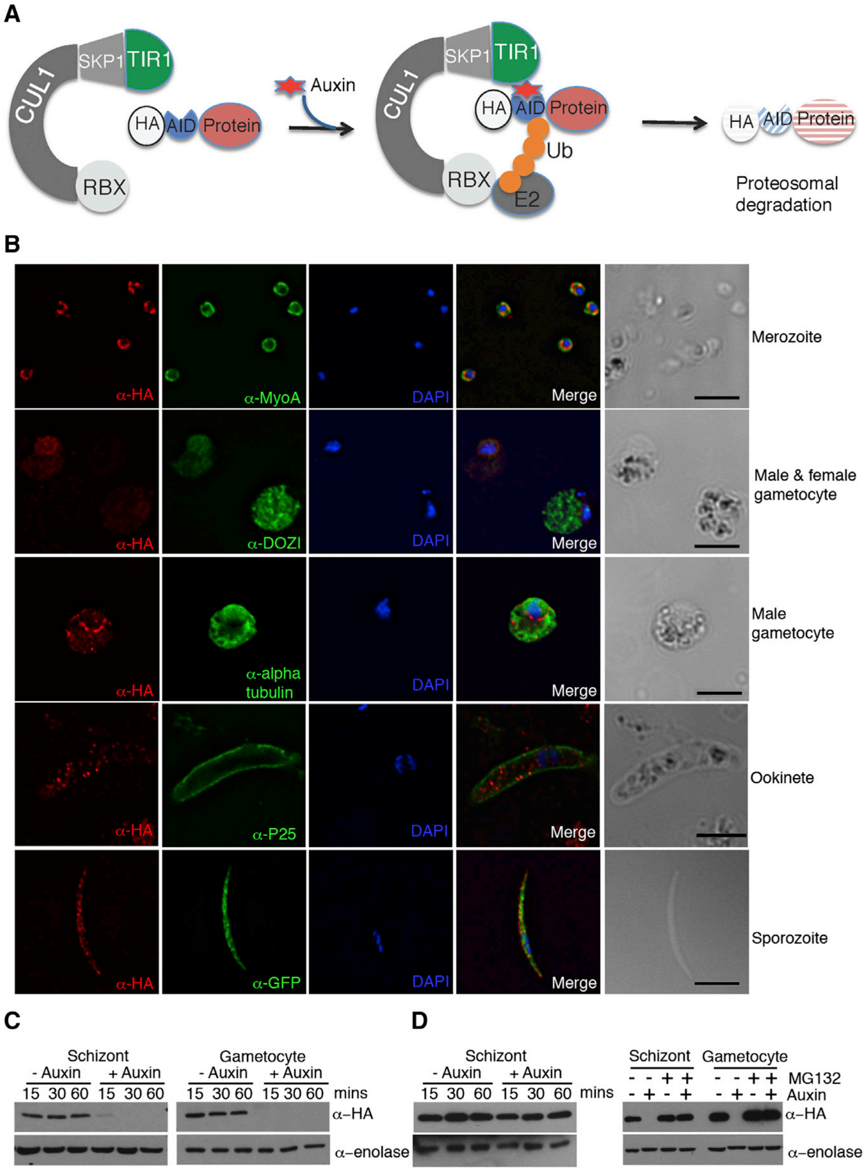

Figure 1. Generation of a Functional AID System in *Plasmodium berghei* to Examine Calcineurin Function

(A) Auxin promotes interaction of TIR1 (an F box protein, in green) with the AID degron tagged protein (blue). The AID-tagged protein (red) is recruited to the Skp, Cullin, F-box-containing complex (SCF), a multi-protein E3-ligase complex, resulting in ubiquitination and degradation of the target protein. Schematic adapted from Nishimura et al. (2009).

(B) Expression and localization of PbCnA-AID-HA at the indicated stages of *Plasmodium* life cycle. Fixed and permeablised parasites were probed with indicated primary antibodies. Scale bar, 5 μm.

(C) Robust and efficient depletion of PbCnA-AID-HA, upon addition of auxin in both schizonts and gametocytes, as measured by western blotting. Enolase serves as a loading control.

(D) Conditional depletion of PbCnA-AID-HA is reliant on auxin, TIR1, and the proteasome. PbCnA-AID protein levels in a non-TIR1 background is resistant to auxin-mediated depletion (left panel). Pre-incubation with proteasome inhibitor 1 μM MG132 for 1 hr blocks PbCnA-AID depletion by auxin (right panel), as shown by western blotting. Enolase serves as a loading control.

See also Figure S1.

Moreover, drug-on methods are difficult to maintain in the non-erythrocytic life stages.

Here we report the development and application of a rapid and specific protein degradation tool to examine protein function in *P. berghei.* We adapted an inducible protein depletion method that relies on the proteasome-mediated auxin response pathway in plants (Nishimura et al., 2009). By successful application of this chemical-genetic method, we dissect and reveal the functions of the essential gene, calcineurin, at key transition points of the *Plasmodium* life cycle. We show that calcineurin regulates parasite colonization of diverse host cell types, including erythrocytes, mosquito midgut cells, and hepatocytes, demonstrating the versatility of this technology. We further engineered the degradation system to promote multiplex transgenic parasite generation combined with phenotype analysis. Hence, this inducible, specific, and rapid protein degradation technology significantly enhances the *Plasmodium* research tool kit to study multifunctional or essential genes and provides the community with a resource to facilitate targeted genetic screens.

RESULTS

Auxin-Inducible Degron System Enables Conditional Expression of Calcineurin A in *P. berghei*

Inability to delete CnA (our efforts and Guttery et al., 2014) and the expression profile of the phosphatase at key transition stages of the parasite life cycle (Lindner et al., 2013; Otto et al., 2014; Patra et al., 2008) prompted us to develop a rapid and specific protein regulation tool. The system relies on the highly conserved Skp1, Cullin1, F box protein ubiquitin ligase (SCF) complex where the F box protein recruits specific substrates for degradation. Auxin functions as a molecular glue that promotes and stabilizes physical interaction between the auxin receptor, TIR1 (an F box protein), and proteins containing an auxin-inducible degron (AID) motif (Nishimura et al., 2009) (Figure 1A). Although the TIR1 F box protein is specific to plants, the high degree of conservation of eukaryotic Skp1 proteins is predicted to allow association with ectopically expressed TIR1 to form a functional SCF^{TIR} complex in *Plasmodium* (Figures 1A, S1A, and S1B). We generated two marker-free parent lines expressing *ostir1* controlled by the strong ubiquitously expressing *hsp70* promoter (Figures S1C–S1E) and *pb48/45* or *pb28* 3′ UTRs. The *pb48/45* 3′ UTR controlled line was well suited for phenotyping of blood stages, but it produced lower

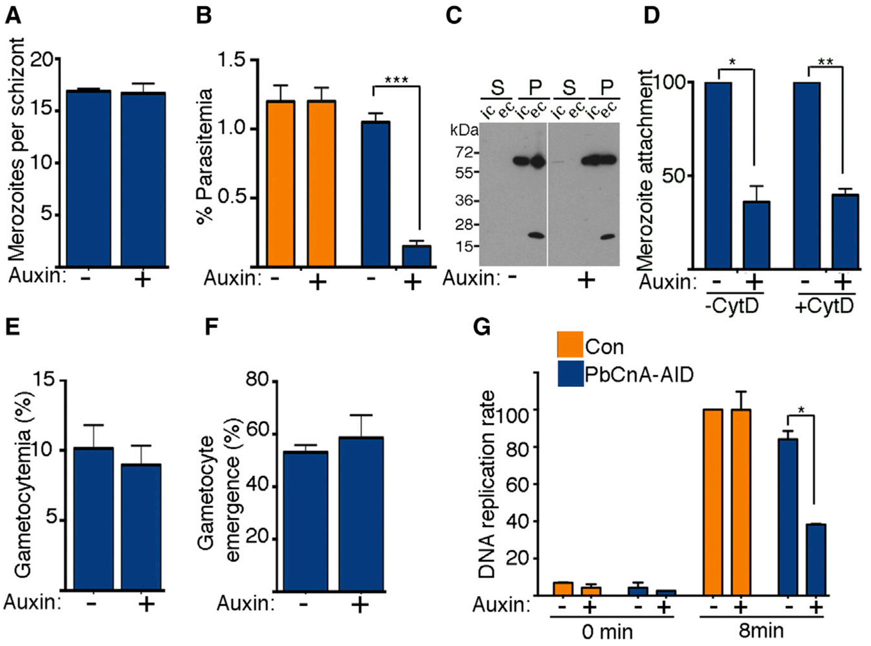

Figure 2. Calcineurin Regulates Erythrocyte Invasion by the Merozoite and Male Gametogenesis

(A) Sustained (24.5 hr) auxin treatment from the ring stage has no effect on the number of merozoites produced in PbCnA-AID parasites (25 segmented schizonts counted per condition, n = 4 experiments).

(B) Pre-incubation of mature PbCnA-AID schizonts with auxin for 30 min results in 86% reduction of in vivo erythrocytic invasion by merozoites as measured by flow cytometry (n = 3 experiments).

(C) AMA1 processing in free merozoites (± auxin) determined by exposing merozoites to extracellular conditions ([ec]; high Na^+ and low K^+) versus intracellular conditions ([ic]; low Na^+ and high K^+) and measured by western blotting. Both supernatant (S) and pellet (P) fractions were probed with mAb28G2, which recognizes the C terminus of AMA-1.

(D) Merozoite attachment to erythrocytes is regulated by PbCnA-AID. Isolated merozoites (± auxin; ± CytD) were incubated with erythrocytes under shaking conditions. After 10 min, cells were fixed and merozoite attachment/invasion was assessed by microscopy (n = 3 experiments with at least ten fields each containing ~350 erythrocytes per experimental condition). See also Figures S2C–S2E.

(E) Gametocyte production is unaffected by PbCnA-AID depletion. Synchronized ring-stage parasites were treated with auxin (15 min post-invasion) and grown ex-vivo, and gametocytemia was determined 32 hr later (n = 4 experiments).

(F) Gametocyte emergence from erythrocytes is unaffected by PbCnA-AID depletion. Gametocytes were pre-treated with auxin for 45 min and subsequently activated for gametogenesis by addition of RPMI + 100 μM xanthurenic acid and a drop in temperature from 37°C to 21°C (n = 2 experiments with 100 gametocytes counted per experimental condition).

(G) Reduction in the number of male gametocytes replicating their DNA with PbCnA-AID depletion. Proportion of male gametocytes undergoing DNA replication was determined at 0 and 8 min post-activation and is expressed as a percentage of the non-auxin-treated control line at 8 min (n = 3 experiments).

Control lines (Con) are OsTIR1 expressing with an unmodified *pbcna* locus (pG230). For all panels, auxin = 500 μM IAA, mean ± SEM; two-tailed t test for paired observations: [*], $p < 0.05$; [**], $p < 0.01$; and [***],$p < 0.001$.

See also Figure S2.

ookinete numbers. This was resolved by utilizing the *pb28* 3′ UTR controlled TIR1 for post-gamete fertilization assays, including motility, microneme secretion, and infectivity of ookinetes and liver stage development of sporozoites. In the OsTIR1-expressing parent lines, *pbcna* (PBANKA_122740) was tagged at the C terminus by *aid-2xha* using single cross-over recombination, which was confirmed by PCR and western blotting (Figures S1F and S1G). Immunofluorescence and western blotting indicated that CnA was expressed at the schizont/merozoite, gametocyte, and sporozoite stages of the parasite life cycle (Figures 1B and 1C). CnA protein was localized to the cytoplasm in all stages. However, in gametocytes it was detected only in males where, in addition to diffused cytoplasmic expression, the protein appeared to form a ring around the nucleus, suggesting CnA has different and/or additional functions in male gametocytes.

Next we tested if CnA fused to the AID-2xHA degron (CnA-AID) could be depleted at both asexual and sexual life cycle stages in an auxin-dependent manner. Schizonts, gametocytes, or sporozoites were incubated with 500 μM auxin for the indicated times. Auxin stimulated efficient degradation of CnA-AID fusions at each of these stages within only 45 min (Figures 1C and S3C) and was dependent on both TIR1 and the proteasome (Figure 1D).

Calcineurin Regulates Merozoite Invasion of Erythrocytes In Vivo

Previous work using indirect Calcineurin inhibitors such as cyclosporin A and FK506 have suggested that Calcineurin is essential during blood stage development. (Dobson et al., 1999; Singh et al., 2014). However, cyclosporin A and FK506 target Calcineurin by associating with either cyclophilins or FK506-binding protein, both of which exhibit prolyl isomerase activity. Therefore, the phenotype observed upon addition of these inhibitors could in part be a pleiotropic effect of prolyl isomerase inhibition. Thus, by specifically depleting CnA protein levels with the AID system, we expected to gain a clearer understanding of its protein function. Indeed, addition of auxin to synchronized ring stage parasites completely depleted CnA levels in schizonts but did not influence intraerythrocytic development from rings into schizonts (Figures S2A and S2B). Moreover, schizonts generated from auxin-treated rings had a normal complement of merozoites, further indicating that CnA is not required for erythrocytic schizogony (Figure 2A). However, when CnA-AID was depleted in mature schizonts and isolated merozoites were intravenously administered to naive mice, we observed a nearly 90% reduction in erythrocyte invasion (Figures 2B and S2A). Hence, AID-mediated depletion of CnA demonstrated the importance of this phosphatase for

invasion of erythrocytes by the parasite under flow conditions in vivo.

Calcineurin Specifically Controls Merozoite Attachment to the Erythrocyte

Merozoite invasion of the erythrocyte is a complex multistep process requiring several merozoite-erythrocyte attachment phases and release of secretory organelles (reviewed in Cowman et al., 2012). To dissect the point of Calcineurin action during merozoite invasion, we developed an in vitro assay to examine processing of the micronemal protein, PbAMA1 (based on Singh et al., 2014), and also to inspect merozoite attachment to the erythrocyte (based on the accompanying manuscript, Paul et al., 2015). Plasmodium AMA1, a transmembrane protein (83 kDa in Pf and 66 kDa in Pb), undergoes multiple proteolytic processing events and is exported to the merozoite surface upon egress from the host cell (Howell et al., 2001; Kocken et al., 1998; Peterson et al., 1989). During erythrocyte invasion the cleaved fragments are shed from the parasite surface while the C-terminal domain is retained in the parasite membrane (Howell et al., 2001). Due to lack of antibodies recognizing the secreted PbAMA-1 fragments, we utilized the C-terminal reactive mAB3G2 to investigate proteolytic processing of PbAMA1. Merozoites were isolated and incubated in either intracellular ([IC]: low Na^+ and high K^+) or extracellular (EC) buffer (high Na^+ and low K^+). Only under EC conditions is the ~20 kDa band corresponding to the C-terminal fragment of PbAMA1 observed (Figure 2C). While the assay could not examine secreted PbAMA1 fragments, it demonstrated that depletion of PbCnA-AID had no detectable effect on levels of the cleaved and membrane-bound PbAMA1 fragment (Figure 2C).

To determine whether calcineurin influenced merozoite attachment or subsequent entry into the host cell, we utilized an actin inhibitor, Cytochalisin D (CytD), which permits merozoite attachment to the erythrocyte but prevents entry (Miller et al., 1979). We incubated purified merozoites with erythrocytes under shaking conditions for 10 min and immediately examined merozoite attachment/invasion of the erythrocyte by microscopy (Figure S2C). Upon PbCnA depletion, both in the presence and absence of CytD, merozoite attachment/invasion to erythrocytes was significantly and equally reduced (Figures 2D and S2D). Furthermore, when ring stage parasitemia resulting from successfully invaded merozoites was assessed 4 hr post-invasion, the reduction in parasitemia was similar to the attachment defect (Figures 2D and S2E).

Taken together, these data reveal that while PbAMA1 processing is independent of calcineurin, the defect in erythrocyte invasion is largely contributed by the influence of calcineurin activity on merozoite attachment to the erythrocyte.

Calcineurin Regulates Male Gametogenesis and Gamete Fertilization

Approximately 5%–20% of blood stage parasites form differentiation-arrested male and female gametocytes, which circulate with asexual stage parasites in the infected host. In vitro conditions that mimic the mosquito vector environment, including a drop in temperature and addition of xanthurenic acid, induce calcium signals in male and female gametocytes to stimulate gametogenesis (Billker et al., 2004; Billker et al., 1998). Upon activation, both male and female gametocytes emerge from erythrocytes, followed by male gametocytes undergoing three rounds of genomic DNA replication, forming eight nuclei that are each packaged into an axoneme-containing microgamete (Janse et al., 1986). Auxin-induced depletion of CnA-AID in ex vivo ring-stage parasites did not influence gametocyte production and maturation (Figure 2E). Similarly, CnA-AID depletion in mature gametocytes prior to activation did not affect gametocyte emergence as assayed by rupture of infected erythrocytes (Figures 2F and S2H). Additionally, in the absence of CnA-AID, typical expression of the translationally repressed ookinete surface protein, P28, and Rab11a GTPase, both of which are markers of activated female gametes, indicated normal female gametogenesis (Figures S2F and S2G). However, CnA-AID depletion in mature gametocytes prior to activation acutely affected male gametogenesis. We observed a ~50% reduction in male gametocytes undergoing DNA replication (Figures 2G and S2I), while the remaining male gametocytes exhibited normal DNA replication and further develop into microgametes (Figure S2J).

In fertilization, a wild-type male microgamete fuses with a female macrogamete to form a zygote. In a population of gametocytes, auxin-induced depletion of CnA-AID reduced the ookinete forms developed from a fertilized zygote by 90% (Figure 3A). Consequently, the levels of both micronemal and glideosome proteins expressed post-fertilization were significantly reduced or abolished (Figure 3B). When compared to the 50% defect in male gametocyte genome replication (Figure 2G), the additional defect in fertility observed with CnA-AID depletion argues that CnA also directly regulates the function or fertility of the remaining apparently mature male gametes (Figure S2J) Taken together, the above data and male gametocyte restricted protein expression (Figure 1B) demonstrate a specific role for CnA in regulating male gametogenesis and subsequent fertilization.

Calcineurin Regulates Life Cycle Transition in Ookinetes and Sporozoites

The *Plasmodium* life cycle has two motile stages: an ookinete stage that is required for switching from the mammalian host to the vector and a sporozoite stage for transitioning from the mosquito vector to the host. Calcium regulates invasion and motility of both these infective stages (Coppi et al., 2007; Siden-Kiamos et al., 2006). Since CnA is expressed in both mature ookinetes and sporozoites, we investigated if it influenced specific characteristics of these motile and invasive forms. When auxin was added 4–6 hr post-fertilization, normal ookinete development was observed, and CnA-AID depletion did not affect ookinete motility or secretion of micronemal proteins such as CTRP or chitinase (Figures 3C, 3D, and S3A) (Philip et al., 2012). However, when CnA-AID-depleted ookinetes were fed to mosquitoes, we observed a significant decrease in oocyst formation in the mosquito midgut, implying a role for CnA in ookinete-to-oocyst transition (Figures 3E and S3B). In order to examine CnA function in the sporozoite, 22 day sporozoites isolated from salivary glands were incubated with auxin for 90 min and deposited on HepG2 liver cells. Utilizing a differential staining method (Sinnis et al., 2013) to distinguish intracellular and extracellular sporozoites, upon CnA-AID depletion, we observed a 29% reduction in sporozoite invasion of HepG2 hepatocytes

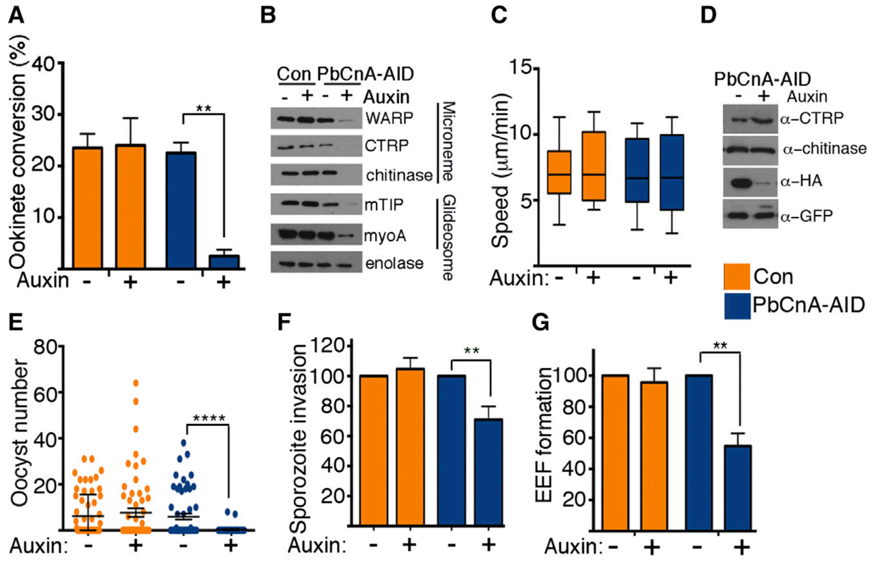

Figure 3. Calcineurin Regulates Gamete Fertilization and Host Cell Colonization of both Ookinetes and Sporozoites

(A) PbCnA-AID depletion in gametocytes resulted in 90% reduction in ookinetes formed. Conversion rate is reported as the percentage of female gametes forming ookinetes (n = 4 experiments).

(B) Following PbCnA-AID depletion prior to gametocyte activation, ookinete micronemal and motor proteins, known to be expressed post-fertilization, become nearly undetectable by western blot. Enolase serves as the loading control.

(C) Ookinete motility (measured by distance covered over time) is unaffected by PbCnA-AID depletion (n = 21 instances per condition; bottom and top of box denote first and third quartiles, respectively; whiskers denote minimum and maximum; p = 0.9 for PbCnA-AID line ± auxin, 0.6 for Con line ± auxin).

(D) Secretion of the micronemal proteins CTRP and chitinase into the supernatant is unaffected by PbCnA-AID depletion in mature ookinetes.

(E) Oocyst numbers in mosquito midgut upon PbCnA-AID depletion in mature ookinetes. Auxin was added to parasite cultures 6 hr post-induction of gametogenesis. Mature ookinetes were fed to mosquitoes, and 7 days later, midguts were dissected and GFP-positive oocysts were counted (n = minimum of 50 mosquitoes for each condition; mean ± SD). Also see independent repeat in Figure S3B.

(F) Sporozoite invasion was examined by incubating mosquito salivary gland sporozoites with auxin (90 min), followed by addition to HepG2 hepatocytes. Sporozoite invasion was assessed 2 hr later and calculated as the proportion of intracellular sporozoites to total sporozoites (intracellular + extracellular). For both control and experimental line, 100% is the proportion of intracellular sporozoites without auxin treatment (n = 2 experiments).

(G) Incubation with auxin (90 min) of mosquito salivary gland sporozoites from a GFP-expressing version of PbCnA-AID parasites reduces EEFs in HepG2 hepatocytes by 46%. For both control and experimental line, 100% is the number of GFP-positive EEFs without auxin treatment (n = 3 experiments).

For all panels, auxin denotes 500 μM IAA; control line (Con) is the TIR1-expressing parent (pG230 for [A] and [B]; pG402 for [C]–[G]); mean ± SEM; two-tailed t test for paired observations: [**], $p < 0.01$; [****], $p < 0.0001$. See also Figure S3.

(Figure 3F). However, CnA-AID depletion reduced the number of exo-erythrocytic forms (EEFs) by 46%, indicating calcineurin requirement during liver stage development (Figures 3G and S3C) and further implying that not all sporozoites that successfully invade hepatocytes develop to recognizable EEFs. Any observed EEFs exhibited normal development (Figures 3G and S3D). The decrease in oocyst and EEF formation due to reduced colonization capacity of ookinetes and sporozoites, respectively, demonstrates the requirement for CnA when the parasite undergoes key life cycle transitions between the host and vector.

Depletion of CDPK1 and PPKL Using AID Replicates Gene Deletion Studies

To demonstrate broader applicability of the AID protein depletion system, we applied the technology to two protein phosphorylation-modulating enzymes with published gene manipulation studies. The two targets were CDPK1, which is the focus of significant drug development efforts, and the protein phosphatase with kelch-like (PPKL) domains.

Employing a similar strategy used to generate PbCnA-AID tagged parasites, we C-terminally tagged endogenous *pbcdpk1* and *pbppkl* with the *aid* degron (Figures S4A–S4C). Treatment with auxin for 45 min led to strong depletion of CDPK1-AID and PPKL-AID proteins in both schizonts and gametocytes stages, thereby confirming the application potential of this technology to both proteins and stages (Figures 4A and 4D). Previous gene deletion studies indicated *P. berghei* CDPK1 and PPKL are not essential for the parasite's asexual blood-stage development (Guttery et al., 2012; Jebiwott et al., 2013; Philip et al., 2012). Similarly, in our experiments sustained application of auxin starting at ring stage parasites of both lines had no effect on schizogony (Figure 4B). Moreover, depletion of either CDPK1 or PPKL in mature schizonts, followed by intravenous administration to mice, did not influence erythrocyte invasion (Figure 4C). Gene deletion (*ppkl*) and conditional expression (*cdpk1*) in *P. berghei* established that PPKL and CDPK1 regulate ookinete morphology (Guttery et al., 2012; Philip et al., 2012; Sebastian et al., 2012). Depletion of PPKL-AID or CDPK1-AID in mature gametocytes followed by induction of gametogenesis also resulted in abnormal ookinete forms comprising primarily of spheres or retorts (Figures 4E and 4F). During zygote-to-ookinete development, CDPK1 influences translationally controlled expression of ookinete proteins. Similarly, CDPK1-AID depletion displayed significantly reduced levels of several ookinete micronemal proteins, including WARP, chitinase, and CTRP (Figure 4G). Collectively, the phenotypic analysis of asexual and sexual stage development by the AID technology largely mirrors the gene deletion and manipulation reports for both PbCDPK1 and PbPPKL, indicating its broad applicability to studies of gene function in *P. berghei*.

A Multiplexed AID System for Medium-Throughput Phenotyping

The traditional approach to generate a cloned transgenic parasite line requires individually injecting single IRBCs into ten naive

CellPress

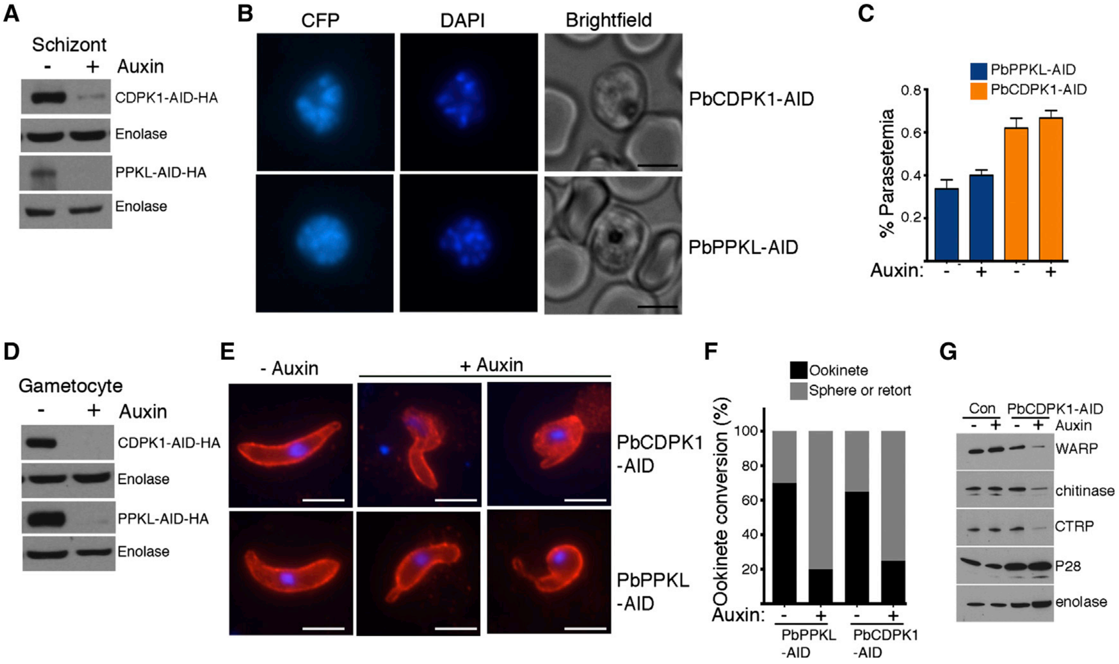

Figure 4. Conditional Protein Depletion Demonstrates CDPK1 and PPKL Are Dispensable for Asexual Growth but Crucial for Ookinete Development

(A) Robust depletion of both CDPK1 and PPKL protein with C-terminal AID-HA epitope tags, upon addition of auxin for 45 min in schizonts, as measured by western blotting. Enolase serves as the loading control.

(B) Continuous treatment with auxin (24.5 hr) from ring stage has no effect on schizogony in both PbCDPK1-AID and PbPPKL-AID parasite lines. Scale bar, 5 μm.

(C) Pre-incubation of mature PbCDPK1-AID or PbPPKL-AID schizonts with auxin for 45 min had no effect on erythrocytic invasion by merozoites as measured by flow cytometry (n = 3 experiments)

(D) Robust depletion of both CDPK1 and PPKL protein with C-terminal AID-HA epitope tags, upon addition of auxin for 45 min in gametocytes, as measured by western blotting. Enolase serves as the loading control.

(E) PbCDPK1-AID or PbPPKL-AID depletion in gametocytes resulted in abnormal ookinete formation. Zygote-to-ookinete development was visualized by probing against the surface protein marker, P25 (in red, DAPI: blue).

(F) Proportion of spherical/retort and normal ookinete forms observed upon PbPPKL or PbCDPK1 depletion. Gametocytes were pre-incubated with auxin for 45 min followed by induction of gametogenesis. Zygote-to-ookinete conversion was determined by examining and counting cells stained with α-P25 antibody. Scale bar, 5 μm.

(G) CDPK1 regulates protein levels of translationally controlled genes during zygote-to-ookinete development. Gametocytes were incubated with auxin for 45 min prior to induction of gametogenesis. 24 hr later, parasite pellets were examined by western blotting. While expression of P-28 was unaffected in PbCDPK1-AID-depleted parasites, WARP, CTRP, and Chitinase levels were significantly reduced. Enolase serves as loading control.

See also Figure S4.

mice. Recently, a flow-cytometry-assisted method was used to isolate isogenic parasite lines resulting in a reduction of animal use by 80% (Kenthirapalan et al., 2012). The AID-tagging plasmid expressing a fluorescent marker can be exploited similarly to isolate isogenic lines by FACs instead of the traditional cloning method (Figure S4A). The bidirectional eef1α promoter was used to drive both drug selection and fluorescent markers, thereby avoiding the use of two separate promoters. Two significant features of the AID-tagging plasmid, which include the fluorescence marker and generation of a regulatable target protein, lend this technology to medium-throughput generation and phenotyping of transgenic parasite lines and a further reduction in animal use.

In a demonstration of the approach, three AID-tagging plasmids were generated expressing GFP, CFP, or mCHERRY fluorescence (Figure 5A). About 1 kb of C terminus of *pbcna*, *pbcdpk1*, and *pbdozi* were cloned into the plasmids to generate AID-tagged fusions that co-expressed with GFP, CFP, and mCHERRY, respectively (Figure S5A). After linearization all three plasmids were mixed, electroporated, and administered into a single mouse. By 8 days post-transfection, parasites expressing exclusively either GFP, CFP, or mCHERRY were observed (Figure 5B). Parasites were collected, and correct plasmid integration was confirmed by PCR (data not shown). Flow cytometry performed on blood from a mixed infection illustrated the three fluorescence markers were easily distinguishable (Figure 5C). Post-sorting, 50 parasites expressing each fluorescent protein from the indicated gates in Figure 5C were intravenously administered to naive mice. Genetic analysis of the resulting parasites indicated that no wild-type or cross-contamination had occurred in the isolated lines, demonstrating the efficiency and robustness of the isolation procedure (Figures S5B and S5C). Not only do the different fluorescence markers enable generation and isolation of the transgenic lines, they facilitate phenotyping (Figures 5D–5G). We performed a duplexed erythrocyte invasion assay where PbCnA-AID (GFP-expressing) and PbCDPK1-AID

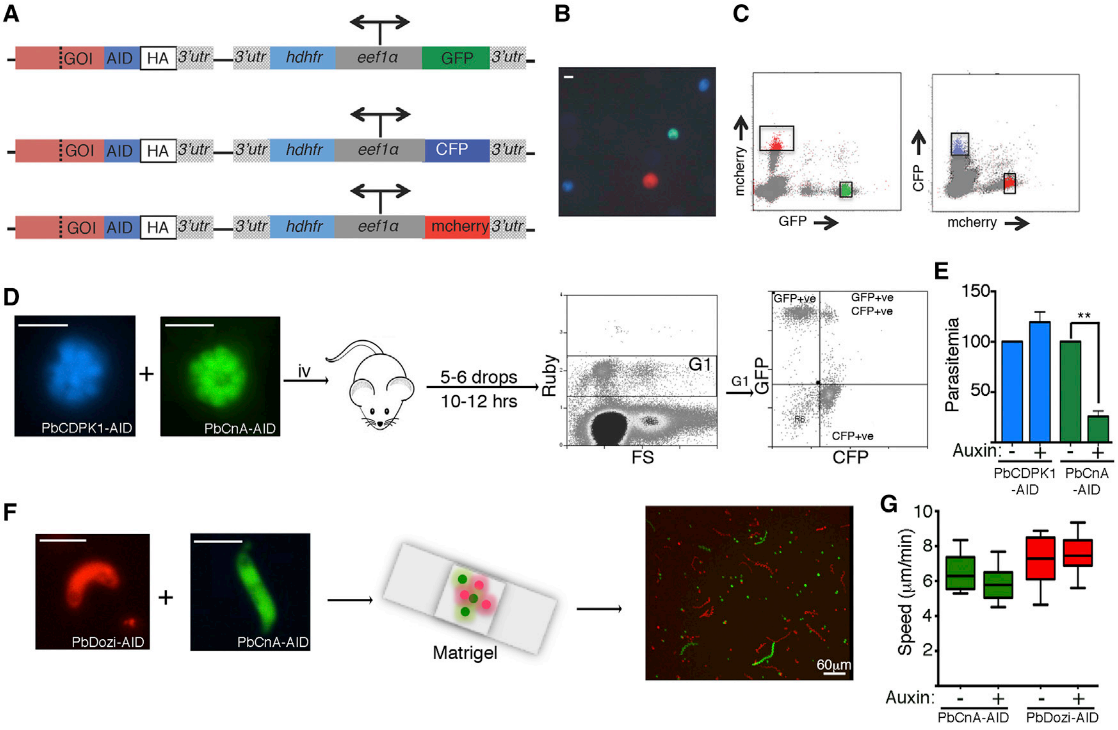

Figure 5. Multiplex Generation and Phenotyping of Transgenic Lines

(A) Schematic of vectors generated for simultaneous transfection and generation of multiple transgenic lines. In addition to C-terminally tagging the gene of interest with AID-2xHA, the plasmid expresses the drug selectable marker (*hdhfr*) and a fluorescence marker (*gfp*, *cfp*, or *mcherry*) driven by the bidirectional *pbeef1α* promoter.

(B) Fluorescent parasites indicate successful generation of three transgenic lines 8 days post-transfection.

(C) Graph of FACS where gates indicate the three types of collected fluorescence-positive cells. Fifty IRBCs of each fluorescence type were intravenously administered to three naive mice to generate GFP-, CFP-, and mCherry-expressing isogenic parasite lines, respectively.

(D) Schematic of duplexed in vivo invasion assay where CFP-expressing PbCDPK1-AID schizonts are mixed with GFP-expressing PbCnA-AID line prior to intravenous injection. 15 min later, four to five blood drops are collected by tail prick and cultured for 12 hr followed by flow cytometry analysis. The gated (G) DiCycle ruby positive (infected IRBC) can be further distinguished into either singly or dually fluorescent populations to determine parasitemia.

(E) Duplexed in vivo invasion assay where CFP-expressing PbCDPK1-AID and GFP-expressing PbCnA-AID (± auxin) are mixed and intravenously injected (mean ± SEM; two tailed t test for paired observations: $p < 0.05$; [**]).

(F) Schematic of duplexed ookinete motility assay where mCherry-expressing PbDozi-AID ookinetes are mixed with GFP expressing PbCnA-AID line in Matrigel. Reconstructed tracks of motile ookinetes with frames collected every 10 s for 15 min (right panel).

(G) Ookinete motility (measured by distance covered over time) was measured for mixed red PbDozi-AID and green PbCnA-AID ookinetes (± auxin) (n = 15 instances per condition; bottom and top of box denote first and third quartiles, respectively; whiskers denote minimum and maximum; p = 0.14 for PbCnA-AID line ± auxin and 0.35 for PbDozi-AID line ± auxin).

Scale bar denotes 5 μm unless otherwise indicated. See also Figure S5.

(CFP-expressing) schizonts were combined and intravenously injected into mice. While PbCnA-AID depletion resulted in significant reduction in erythrocyte invasion, PbCDPK1-AID demonstrated no observable defects (Figure 5E). Additionally a duplexed ookinete motility assay of PbCnA-AID (GFP expressing) and PbDozi-AID (mCHERRY expressing) indicated neither protein performed essential functions needed for ookinete motility (Figure 5G). Thus, this multiplexed AID system is a significant resource toward scaling up analysis of the parasite phenome, reducing both time for analysis and animal use.

DISCUSSION

Advances in the mechanistic understanding of biological processes are largely powered by innovations in tools to manipulate the biological system of study. Gene function can be assigned to a biological process by regulating gene expression. However, genetic manipulation in *Plasmodium* is performed in the haploid asexual blood stage, and disruption of genes essential for asexual blood stage development cannot be recovered for functional analysis. Therefore, deducing functions of essential and multifunctional genes at different stages of parasite development requires robust conditional expression technologies to propagate the parasite and scrutinize gene action. The genetically tractable rodent malaria parasite, *P. berghei*, allows both in vitro and in vivo experimental access to its complete life cycle, providing a powerful platform upon which a versatile protein regulation tool can be imposed. Here we developed a chemical-genetic tool that combines targeted gene manipulation techniques and the rapid speed of small molecule action. Advantages of AID technology include the low cost of the auxin ligand in comparison to Shield (for ddFKBP) and overcoming the

genetic requirement of human DHFR (for TMP), which is already the most widely used positive selection marker for *P. berghei* genetic modification.

To test if the AID system is indeed a powerful resource for studying multistage-specific and essential gene functions, we analyzed the role of calcineurin. While calcineurin plays pivotal roles in calcium-dependent signal transduction pathways in a variety of eukaryotes, its stage-specific roles in *P. berghei* have remained elusive. Because the AID system was active at both 37°C and 21°C, we were able to examine calcineurin function in the host and mosquito stages of the parasite, including the merozoite, gamete, ookinete, and sporozoite (Figures 1–3). Calcineurin is required for DNA replication in male gametes and is a component of the essential calcium-responsive signaling network unique to *Plasmodium* male gametogenesis (Billker et al., 2004). Furthermore, calcineurin also functions downstream in gamete fertility (Figures 2F and 2G). Gamete formation and zygote-to-ookinete development were previously reported to require calcium-coordinated signaling (Sebastian et al., 2012). The important role of calcineurin in gamete fertility uncovered here further underscores the central nature of calcium involvement in *Plasmodium* biology.

Although targeting different host cell types, the merozoite, ookinete, and sporozoite parasite forms invade and develop by employing conserved molecular mechanisms, the majority of which are coordinated by calcium (Billker et al., 2009). All three invasive forms contain an actin/myosin-driven motor complex, the glideosome, and specialized microneme organelles, which secrete adhesins required for engagement with host cell receptors during parasite invasion and motility (Baum et al., 2008). Our data demonstrating that erythrocyte invasion by the merozoite, mosquito midgut invasion by the ookinete, and hepatocyte invasion by the sporozoite are all regulated in a calcineurin-dependent manner establishes calcineurin as an additional unifying factor for different host cell transitions or colonisations. Moreover, our observations in the ookinete and merozoite as well as studies in the *P. falciparum* merozoite and a related apicomplexan parasite, *Toxoplasma gondi* tachyzoite (Paul et al., 2015, accompanying paper), further delineate the requirement of calcineurin for invasion being independent of microneme exocytosis and parasite motility. Further analysis revealed that calcineurin plays a specific and conserved role in the attachment phase of the merozoite to the target erythrocyte. Therefore, uncoupling calcineurin function from organelle secretion and motility reveals additional complexity in calcium-controlled signaling during host cell invasion.

A significant and validating feature of the AID system when applied to signaling proteins PPKL and CDPK1 was its capacity to replicate previously reported phenotypes produced by genetic manipulation efforts in *P. berghei*. Accordingly, our data showed both proteins were dispensable for intraerythrocytic development, but performed critical functions in ookinete development, thereby affecting parasite transmission. Moreover, the AID system could efficiently deplete proteins localized to different cellular compartments. Upon auxin application, cytosolic proteins were robustly degraded as exemplified by depletion of calcinuerin in all examined parasite stages and PPKL in schizonts and gametocytes (Figures 1C, 4A, and 4D). Additionally, the plasma membrane localized CDPK1 in mature schizonts and gametocytes (Green et al., 2008; Sebastian et al., 2012) was susceptible to AID-regulated degradation (Figures 4A and 4D). In mammalian cells, the AID system could efficiently remove nuclear proteins including chromatin-bound ones (Holland et al., 2012). We believe the presence of the proteasome in the parasite nucleus (Oehring et al., 2012) would also allow for AID-mediated depletion of nuclear proteins. Furthermore, detection of a functional 20S proteasome in mature erythrocytes (Neelam et al., 2011) raises the enticing prospect of adapting this technology for parasite proteins exported to the erythrocyte. Recent advancements in gene editing using the CRISPR-Cas9 system and a report implying activity of the AID system in *P. falciparum* (Ghorbal et al., 2014; Kreidenweiss et al., 2013; Wagner et al., 2014) should permit application of the degradation technology to effectively examine endogenous protein function in the human malaria parasite.

We also engineered the degron-tagging plasmid for simultaneous generation of multiple transgenic lines. By using distinct fluorescence markers, we were able to target three different genes in a single transfection experiment and rapidly generate isogenic lines by flow cytometry (Figure 5). The distinct fluorescence markers also improved phenotyping because multiple transgenic or control lines can be used for examining processes that are inherently prone to experimental variation. Improved experimental design by employing multiple fluorescent lines ensures reproducibility that could result in significant reduction in sample size while providing robust statistics. Overall, a remarkable reduction in animal use can be achieved including 85% cut for parasite line generation and at least a further 50% for phenotypic analysis.

By applying and optimizing the AID technology to *P. berghei*, we now have a powerful and specific tool to examine endogenous protein function during multiple stages of parasite development. We showed that calcineurin controls critical parasite developmental switches in both the host and vector, implicating this phosphatase as a promising candidate for therapeutic interventions targeting both parasite development and transmission.

EXPERIMENTAL PROCEDURES

The Supplemental Experimental Procedures provides a list of all reagents, generation of transgenic lines, and detailed description of all techniques used in this study. Two-tailed t test for paired observations was used for all statistical analyses.

Ethics Statement

All animal procedures were carried out according to UK Home Office regulations and protocols approved by the University of Glasgow Ethics Committee.

Invasion Assays

For erythrocyte invasion, purified schizonts were incubated ± 500 μM indole 3-acetic acid (IAA) for 30 min. Merozoites were isolated by filtration and intravenously administered to mice. 15 min post-injection, blood was collected from mice, confirmed ring-stage parasites by microscopy, cultured infected erythrocytes ex vivo for 10 to 15 hr, and measured parasitemia by flow-cytometry. For in vitro attachment/invasion assays, merozoites were incubated with erythrocytes under shaking conditions for 10 min, fixed in 4% PFA, and examined by microscopy. For PbAMA-1 processing assays, purified merozoites were either incubated in buffers mimicking extracellular (high Na^+, low K^+) or intracellular (low Na^+, high K^+) conditions. Pellet lysates were examined by western blotting to detect the processed ~20 kDa AMA-1 protein.

For mosquito midgut infection, ookinetes grown (±IAA) were membrane fed to mosquitoes, and midguts were dissected 7 days later for oocyst counts. For HepG2 infection assays 22 day post-transmission, sporozoites were isolated, pre-treated (±IAA), and incubated with HepG2 cells. Intracellular sporozoites and subsequent EEFs were examined 2 and 48 hr post-invasion, respectively.

Phenotypic Analysis of Sexual-Stage Development

Gametocytes were pre-incubated in schizont media (±IAA) for 30 min at 37°C prior to activation. Cultures were spun down and incubated with ookinete media (±IAA) for further post-activation analysis. Exflagellation assays and ookinete conversion rates were assessed as described (Philip et al., 2012). DNA replication assay and analysis by FACs was performed as described (Laurentino et al., 2011). We assayed gametocyte emergence by measuring rupture of erythrocytes pre-labeled with a fluorescent α-mTER119 antibody by fluorescence microscopy.

Ookinete Microneme Secretion and Motility Assays

For the microneme secretion assay, fertilized zygotes 4 hr post-gametocyte activation were incubated ± IAA. Ookinetes were purified on a LD50 magnetic column 15 hr later and incubated in PBS ± IAA for an additional 4 hr. The supernatants were examined as previously reported (Philip et al., 2012). Ookinetes for motility assays were handled similarly to the microneme secretion assay. The purified ookinetes (±IAA from 4 to 6 hr post-activation) were embedded in Matrigel. Samples were incubated for 1 hr at 21°C before imaging. Time-lapse movies were acquired every 10 s for 15 min on a Leica M205 FA fluorescence stereomicroscope.

Flow Cytometric Isolation of Transgenic Parasites

Parasites individually expressing the three fluorescence markers (GFP, CFP, and mCHERRY) were propagated in mice until the parasitemia reached 0.2%–1.0%. IRBCs were suspended in schizont media, and sorting was performed on a BD-FACSAria III cell sorter. 1,000 IRBCs for each fluorescence were collected, and 50 cells were intravenously administered into mice. Successful isolation and purification of parasites were confirmed by microscopy and integration PCRs.

For additional details for all assays, see Supplemental Experimental Procedures.

SUPPLEMENTAL INFORMATION

Supplemental Information includes five figures, one table, and Supplemental Experimental Procedures and can be found with this article online at http://dx.doi.org/10.1016/j.chom.2015.05.018.

AUTHOR CONTRIBUTIONS

N.P. designed and performed experiments, analyzed the data, and wrote the manuscript A.P.W. supervised the study and wrote the manuscript.

ACKNOWLEDGMENTS

We thank Drs Aditya Paul, Manoj Duraisingh, and Steven Spoel for valuable inputs to the study and critical reading of the manuscript. We also thank Diane Vaughn and the 3Is flow cytometry facility for assistance with cell sorting. We thank NBRP, Japan for the TIR1 and AID plasmids. We are appreciative of the following researchers for providing antibodies: Prof. Clemens Kocken and Annemarie van der Wel (α-AMA1), Prof. Friedrich Frischknecht and Drs. Ann-Kristin Müller and Kirsten Heiß (α-PbCSP), and Prof. Volker Huessler (α-PbEXP1). This study was supported by an EMBO LTF (N.P.), the Wellcome Trust 083811/Z/07/Z (A.P.W.), and the European Commission "Evimalar" ref 242095 (A.P.W.).

Received: December 16, 2014
Revised: April 29, 2015
Accepted: May 27, 2015
Published: June 25, 2015

REFERENCES

Armstrong, C.M., and Goldberg, D.E. (2007). An FKBP destabilization domain modulates protein levels in Plasmodium falciparum. Nat. Methods *4*, 1007–1009.

Bansal, A., Singh, S., More, K.R., Hans, D., Nangalia, K., Yogavel, M., Sharma, A., and Chitnis, C.E. (2013). Characterization of Plasmodium falciparum calcium-dependent protein kinase 1 (PfCDPK1) and its role in microneme secretion during erythrocyte invasion. J. Biol. Chem. *288*, 1590–1602.

Baum, J., Gilberger, T.W., Frischknecht, F., and Meissner, M. (2008). Host-cell invasion by malaria parasites: insights from Plasmodium and Toxoplasma. Trends Parasitol. *24*, 557–563.

Billker, O., Lindo, V., Panico, M., Etienne, A.E., Paxton, T., Dell, A., Rogers, M., Sinden, R.E., and Morris, H.R. (1998). Identification of xanthurenic acid as the putative inducer of malaria development in the mosquito. Nature *392*, 289–292.

Billker, O., Dechamps, S., Tewari, R., Wenig, G., Franke-Fayard, B., and Brinkmann, V. (2004). Calcium and a calcium-dependent protein kinase regulate gamete formation and mosquito transmission in a malaria parasite. Cell *117*, 503–514.

Billker, O., Lourido, S., and Sibley, L.D. (2009). Calcium-dependent signaling and kinases in apicomplexan parasites. Cell Host Microbe *5*, 612–622.

Collins, C.R., Das, S., Wong, E.H., Andenmatten, N., Stallmach, R., Hackett, F., Herman, J.P., Müller, S., Meissner, M., and Blackman, M.J. (2013). Robust inducible Cre recombinase activity in the human malaria parasite Plasmodium falciparum enables efficient gene deletion within a single asexual erythrocytic growth cycle. Mol. Microbiol. *88*, 687–701.

Combe, A., Giovannini, D., Carvalho, T.G., Spath, S., Boisson, B., Loussert, C., Thiberge, S., Lacroix, C., Gueirard, P., and Ménard, R. (2009). Clonal conditional mutagenesis in malaria parasites. Cell Host Microbe *5*, 386–396.

Coppi, A., Tewari, R., Bishop, J.R., Bennett, B.L., Lawrence, R., Esko, J.D., Billker, O., and Sinnis, P. (2007). Heparan sulfate proteoglycans provide a signal to Plasmodium sporozoites to stop migrating and productively invade host cells. Cell Host Microbe *2*, 316–327.

Cowman, A.F., Berry, D., and Baum, J. (2012). The cellular and molecular basis for malaria parasite invasion of the human red blood cell. J. Cell Biol. *198*, 961–971.

Dobson, S., May, T., Berriman, M., Del Vecchio, C., Fairlamb, A.H., Chakrabarti, D., and Barik, S. (1999). Characterization of protein Ser/Thr phosphatases of the malaria parasite, Plasmodium falciparum: inhibition of the parasitic calcineurin by cyclophilin-cyclosporin complex. Mol. Biochem. Parasitol. *99*, 167–181.

Dvorin, J.D., Martyn, D.C., Patel, S.D., Grimley, J.S., Collins, C.R., Hopp, C.S., Bright, A.T., Westenberger, S., Winzeler, E., Blackman, M.J., et al. (2010). A plant-like kinase in Plasmodium falciparum regulates parasite egress from erythrocytes. Science *328*, 910–912.

Ghorbal, M., Gorman, M., Macpherson, C.R., Martins, R.M., Scherf, A., and Lopez-Rubio, J.J. (2014). Genome editing in the human malaria parasite Plasmodium falciparum using the CRISPR-Cas9 system. Nat. Biotechnol. *32*, 819–821.

Goldfless, S.J., Wagner, J.C., and Niles, J.C. (2014). Versatile control of Plasmodium falciparum gene expression with an inducible protein-RNA interaction. Nat. Commun. *5*, 5329.

Green, J.L., Rees-Channer, R.R., Howell, S.A., Martin, S.R., Knuepfer, E., Taylor, H.M., Grainger, M., and Holder, A.A. (2008). The motor complex of Plasmodium falciparum: phosphorylation by a calcium-dependent protein kinase. J. Biol. Chem. *283*, 30980–30989.

Guttery, D.S., Poulin, B., Ferguson, D.J., Szöőr, B., Wickstead, B., Carroll, P.L., Ramakrishnan, C., Brady, D., Patzewitz, E.M., Straschil, U., et al. (2012). A unique protein phosphatase with kelch-like domains (PPKL) in Plasmodium modulates ookinete differentiation, motility and invasion. PLoS Pathog. *8*, e1002948.

Guttery, D.S., Poulin, B., Ramaprasad, A., Wall, R.J., Ferguson, D.J., Brady, D., Patzewitz, E.M., Whipple, S., Straschil, U., Wright, M.H., et al. (2014). Genome-wide functional analysis of Plasmodium protein phosphatases reveals key regulators of parasite development and differentiation. Cell Host Microbe *16*, 128–140.

Harmon, A.C., Gribskov, M., and Harper, J.F. (2000). CDPKs - a kinase for every Ca2+ signal? Trends Plant Sci. *5*, 154–159.

Holland, A.J., Fachinetti, D., Han, J.S., and Cleveland, D.W. (2012). Inducible, reversible system for the rapid and complete degradation of proteins in mammalian cells. Proc. Natl. Acad. Sci. USA *109*, E3350–E3357.

Howell, S.A., Withers-Martinez, C., Kocken, C.H., Thomas, A.W., and Blackman, M.J. (2001). Proteolytic processing and primary structure of Plasmodium falciparum apical membrane antigen-1. J. Biol. Chem. *276*, 31311–31320.

Ishino, T., Orito, Y., Chinzei, Y., and Yuda, M. (2006). A calcium-dependent protein kinase regulates Plasmodium ookinete access to the midgut epithelial cell. Mol. Microbiol. *59*, 1175–1184.

Janse, C.J., van der Klooster, P.F., van der Kaay, H.J., van der Ploeg, M., and Overdulve, J.P. (1986). DNA synthesis in Plasmodium berghei during asexual and sexual development. Mol. Biochem. Parasitol. *20*, 173–182.

Jebiwott, S., Govindaswamy, K., Mbugua, A., and Bhanot, P. (2013). Plasmodium berghei calcium dependent protein kinase 1 is not required for host cell invasion. PLoS ONE *8*, e79171.

Kenthirapalan, S., Waters, A.P., Matuschewski, K., and Kooij, T.W. (2012). Flow cytometry-assisted rapid isolation of recombinant Plasmodium berghei parasites exemplified by functional analysis of aquaglyceroporin. Int. J. Parasitol. *42*, 1185–1192.

Kocken, C.H., van der Wel, A.M., Dubbeld, M.A., Narum, D.L., van de Rijke, F.M., van Gemert, G.J., van der Linde, X., Bannister, L.H., Janse, C., Waters, A.P., and Thomas, A.W. (1998). Precise timing of expression of a Plasmodium falciparum-derived transgene in Plasmodium berghei is a critical determinant of subsequent subcellular localization. J. Biol. Chem. *273*, 15119–15124.

Kreidenweiss, A., Hopkins, A.V., and Mordmüller, B. (2013). 2A and the auxin-based degron system facilitate control of protein levels in Plasmodium falciparum. PLoS ONE *8*, e78661.

Laurentino, E.C., Taylor, S., Mair, G.R., Lasonder, E., Bartfai, R., Stunnenberg, H.G., Kroeze, H., Ramesar, J., Franke-Fayard, B., Khan, S.M., et al. (2011). Experimentally controlled downregulation of the histone chaperone FACT in Plasmodium berghei reveals that it is critical to male gamete fertility. Cell. Microbiol. *13*, 1956–1974.

Lindner, S.E., Swearingen, K.E., Harupa, A., Vaughan, A.M., Sinnis, P., Moritz, R.L., and Kappe, S.H. (2013). Total and putative surface proteomics of malaria parasite salivary gland sporozoites. Mol. Cell. Proteomics *12*, 1127–1143.

Meissner, M., Krejany, E., Gilson, P.R., de Koning-Ward, T.F., Soldati, D., and Crabb, B.S. (2005). Tetracycline analogue-regulated transgene expression in Plasmodium falciparum blood stages using Toxoplasma gondii transactivators. Proc. Natl. Acad. Sci. USA *102*, 2980–2985.

Miller, L.H., Aikawa, M., Johnson, J.G., and Shiroishi, T. (1979). Interaction between cytochalasin B-treated malarial parasites and erythrocytes. Attachment and junction formation. J. Exp. Med. *149*, 172–184.

Muralidharan, V., Oksman, A., Iwamoto, M., Wandless, T.J., and Goldberg, D.E. (2011). Asparagine repeat function in a Plasmodium falciparum protein assessed via a regulatable fluorescent affinity tag. Proc. Natl. Acad. Sci. USA *108*, 4411–4416.

Neelam, S., Kakhniashvili, D.G., Wilkens, S., Levene, S.D., and Goodman, S.R. (2011). Functional 20S proteasomes in mature human red blood cells. Exp. Biol. Med. (Maywood) *236*, 580–591.

Nishimura, K., Fukagawa, T., Takisawa, H., Kakimoto, T., and Kanemaki, M. (2009). An auxin-based degron system for the rapid depletion of proteins in nonplant cells. Nat. Methods *6*, 917–922.

Oehring, S.C., Woodcroft, B.J., Moes, S., Wetzel, J., Dietz, O., Pulfer, A., Dekiwadia, C., Maeser, P., Flueck, C., Witmer, K., et al. (2012). Organellar proteomics reveals hundreds of novel nuclear proteins in the malaria parasite Plasmodium falciparum. Genome Biol. *13*, R108.

Otto, T.D., Böhme, U., Jackson, A.P., Hunt, M., Franke-Fayard, B., Hoeijmakers, W.A., Religa, A.A., Robertson, L., Sanders, M., Ogun, S.A., et al. (2014). A comprehensive evaluation of rodent malaria parasite genomes and gene expression. BMC Biol. *12*, 86.

Patra, K.P., Johnson, J.R., Cantin, G.T., Yates, J.R., 3rd, and Vinetz, J.M. (2008). Proteomic analysis of zygote and ookinete stages of the avian malaria parasite Plasmodium gallinaceum delineates the homologous proteomes of the lethal human malaria parasite Plasmodium falciparum. Proteomics *8*, 2492–2499.

Paul, A.S., Saha, S., Engelberg, K., Jiang, R.H.Y., Coleman, B.I., Kosber, A.L., Chen, C.-T., Ganter, M., Espy, N., Gilberger, T.W., Gubbels, M.-J., and Duraisingh, M.T. (2015). Parasite calcineurin regulates host cell recognition and attachment by apicomplexans. Cell Host Microbe *18*, this issue, 49–60.

Peterson, M.G., Marshall, V.M., Smythe, J.A., Crewther, P.E., Lew, A., Silva, A., Anders, R.F., and Kemp, D.J. (1989). Integral membrane protein located in the apical complex of Plasmodium falciparum. Mol. Cell. Biol. *9*, 3151–3154.

Philip, N., Vaikkinen, H.J., Tetley, L., and Waters, A.P. (2012). A unique Kelch domain phosphatase in Plasmodium regulates ookinete morphology, motility and invasion. PLoS ONE *7*, e44617.

Pino, P., Sebastian, S., Kim, E.A., Bush, E., Brochet, M., Volkmann, K., Kozlowski, E., Llinás, M., Billker, O., and Soldati-Favre, D. (2012). A tetracycline-repressible transactivator system to study essential genes in malaria parasites. Cell Host Microbe *12*, 824–834.

Rusnak, F., and Mertz, P. (2000). Calcineurin: form and function. Physiol. Rev. *80*, 1483–1521.

Sebastian, S., Brochet, M., Collins, M.O., Schwach, F., Jones, M.L., Goulding, D., Rayner, J.C., Choudhary, J.S., and Billker, O. (2012). A Plasmodium calcium-dependent protein kinase controls zygote development and transmission by translationally activating repressed mRNAs. Cell Host Microbe *12*, 9–19.

Siden-Kiamos, I., Ecker, A., Nybäck, S., Louis, C., Sinden, R.E., and Billker, O. (2006). Plasmodium berghei calcium-dependent protein kinase 3 is required for ookinete gliding motility and mosquito midgut invasion. Mol. Microbiol. *60*, 1355–1363.

Singh, S., More, K.R., and Chitnis, C.E. (2014). Role of calcineurin and actin dynamics in regulated secretion of microneme proteins in Plasmodium falciparum merozoites during erythrocyte invasion. Cell. Microbiol. *16*, 50–63.

Sinnis, P., De La Vega, P., Coppi, A., Krzych, U., and Mota, M.M. (2013). Quantification of sporozoite invasion, migration, and development by microscopy and flow cytometry. Methods Mol. Biol. *923*, 385–400.

Wagner, J.C., Platt, R.J., Goldfless, S.J., Zhang, F., and Niles, J.C. (2014). Efficient CRISPR-Cas9-mediated genome editing in Plasmodium falciparum. Nat. Methods *11*, 915–918.

Wilkes, J.M., and Doerig, C. (2008). The protein-phosphatome of the human malaria parasite Plasmodium falciparum. BMC Genomics *9*, 412.

Cell Host & Microbe, Volume *18*

Supplemental Information

Conditional Degradation of *Plasmodium* Calcineurin Reveals Functions in Parasite Colonization of both Host and Vector

Nisha Philip and Andrew P. Waters

Figure S1

A

Gene	P.berghei	P.falciparum
Skp1	PBANKA_114290	3D7_1367000
Cul1	PBANKA_142650	3D7_0811000
Rbx1	PBANKA_080620	3D7_0319100

B

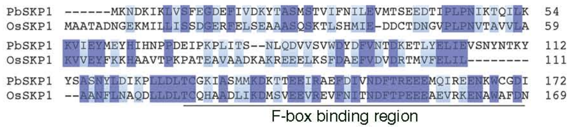

C

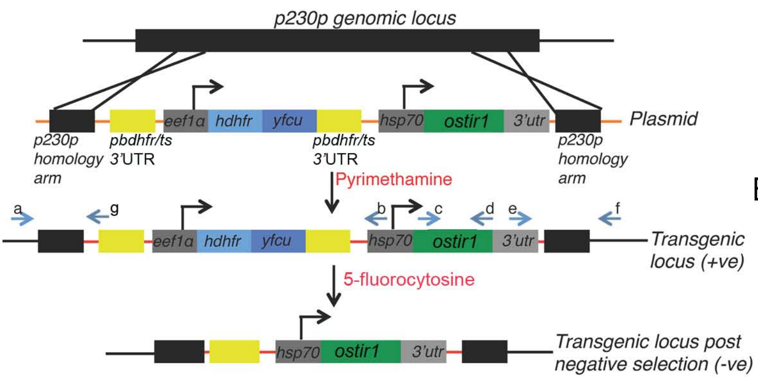

D

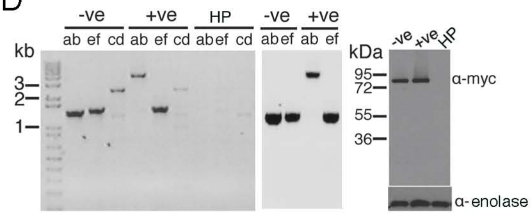

E

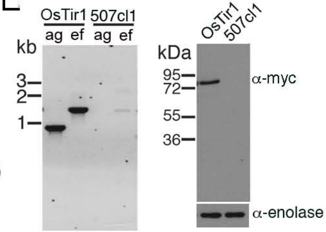

F

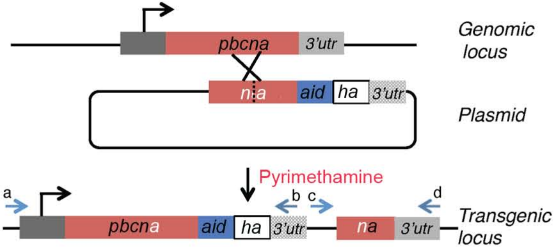

G

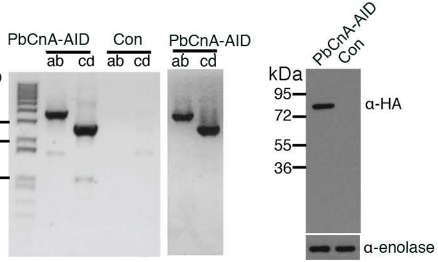

Figure S2

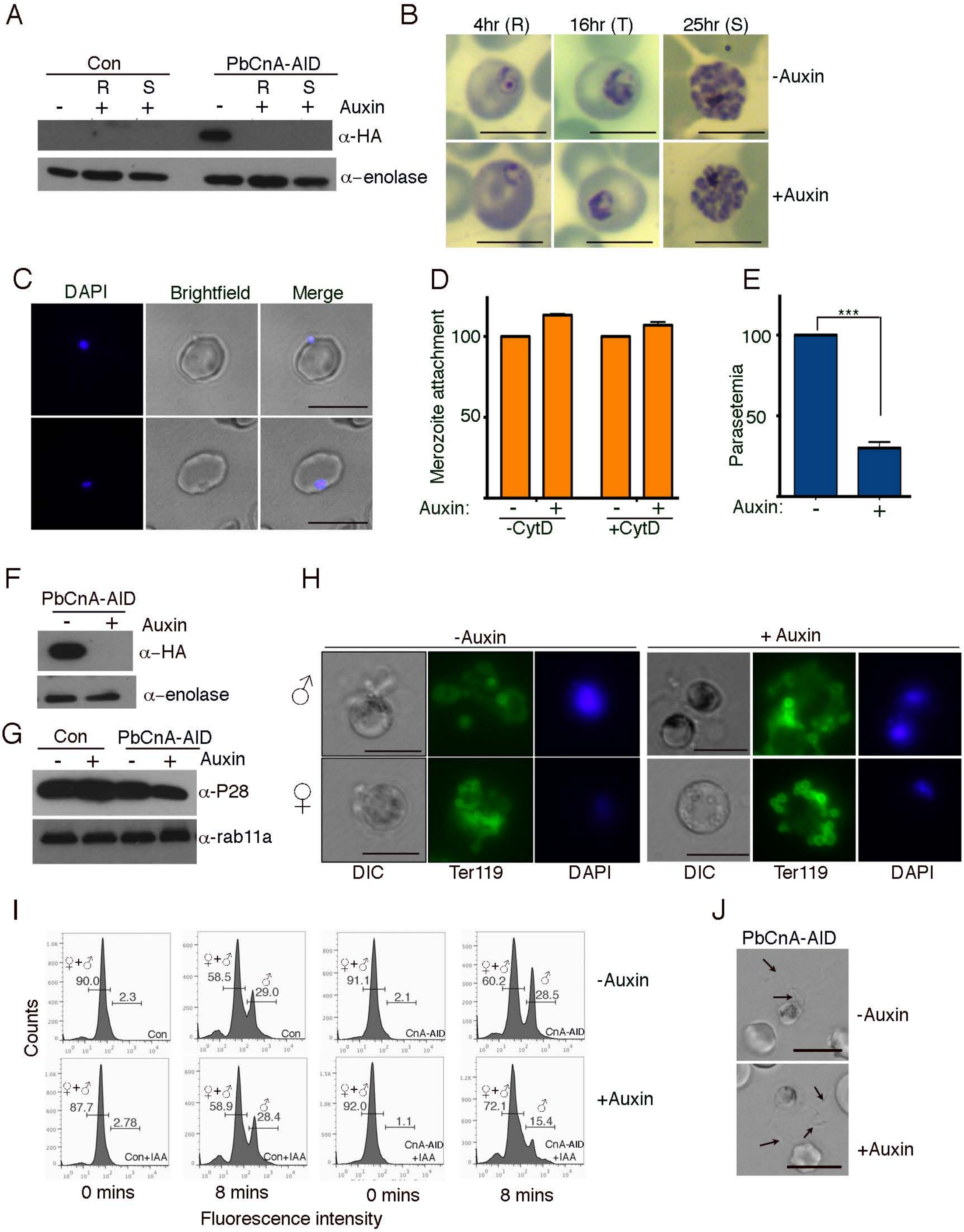

A
Con
PbCnA-AID
R S R S
- + + - + + Auxin
α-HA
α-enolase
B
4hr (R) 16hr (T) 25hr (S)
-Auxin
+Auxin
C
DAPI Brightfield Merge
D
Merozoite attachment
100
50
Auxin: - + - +
-CytD +CytD
E
***
Parasetemia
100
50
Auxin: - +
F
PbCnA-AID
- + Auxin
α-HA
α-enolase
G
Con PbCnA-AID
- + - + Auxin
α-P28
α-rab11a
H
-Auxin
+ Auxin
♂
♀
DIC Ter119 DAPI DIC Ter119 DAPI
I
Counts
♀+♂ 90.0 2.3 Con
♀+♂ 58.5 ♂ 29.0 Con
♀+♂ 91.1 2.1 CnA-AID
♀+♂ 60.2 ♂ 28.5 CnA-AID
-Auxin
♀+♂ 87.7 2.78 Con+IAA
♀+♂ 58.9 ♂ 28.4 Con+IAA
♀+♂ 92.0 1.1 CnA-AID +IAA
♀+♂ 72.1 ♂ 15.4 CnA-AID +IAA
+Auxin
0 mins 8 mins 0 mins 8 mins
Fluorescence intensity
J
PbCnA-AID
-Auxin
+Auxin

Figure S3

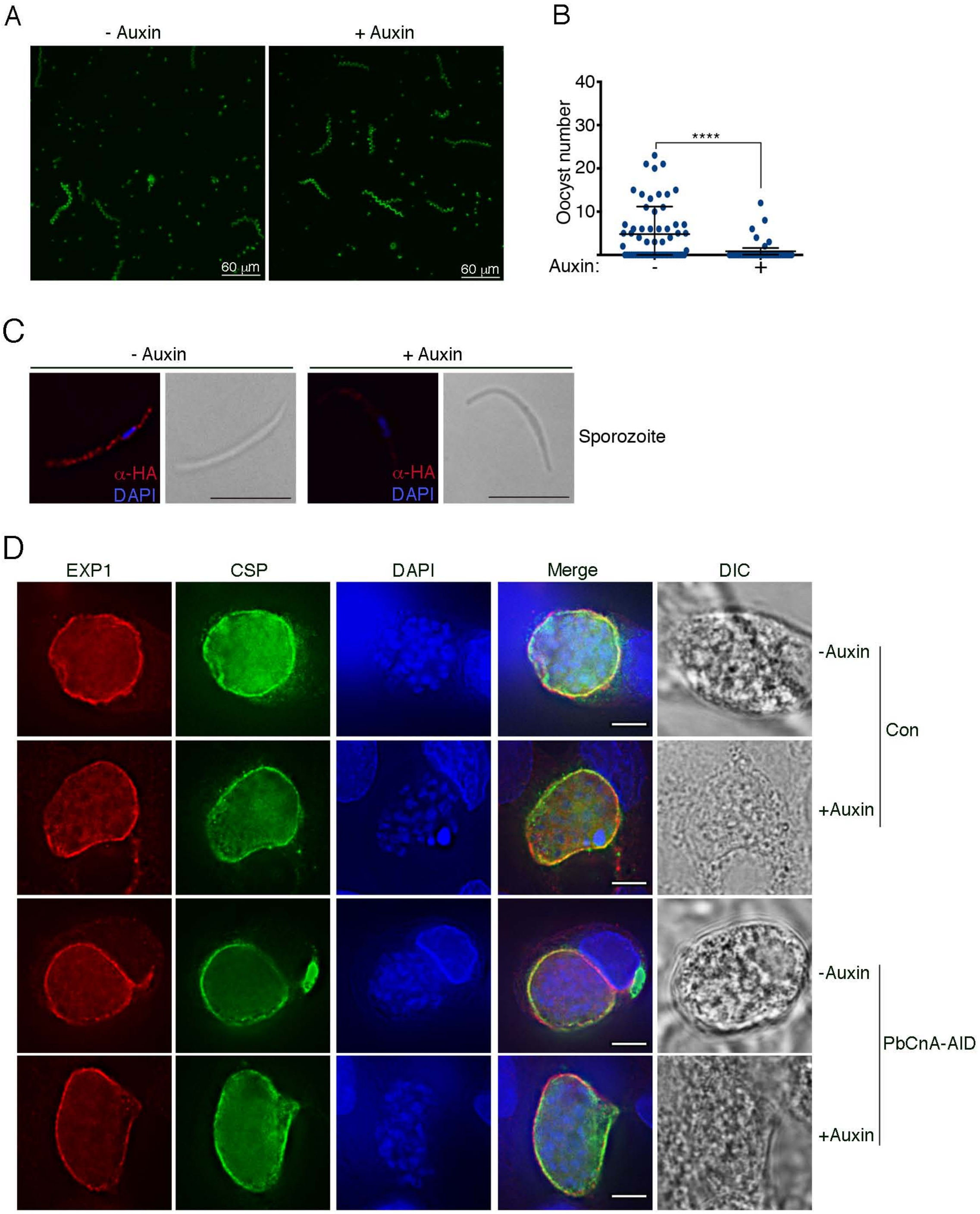
A
- Auxin
+ Auxin
60 μm
60 μm
B
Oocyst number
40
30
20
10
****
Auxin:
-
+
C
- Auxin
+ Auxin
α-HA
DAPI
α-HA
DAPI
Sporozoite
D
EXP1
CSP
DAPI
Merge
DIC
-Auxin
+Auxin
Con
-Auxin
+Auxin
PbCnA-AID

Figure S4

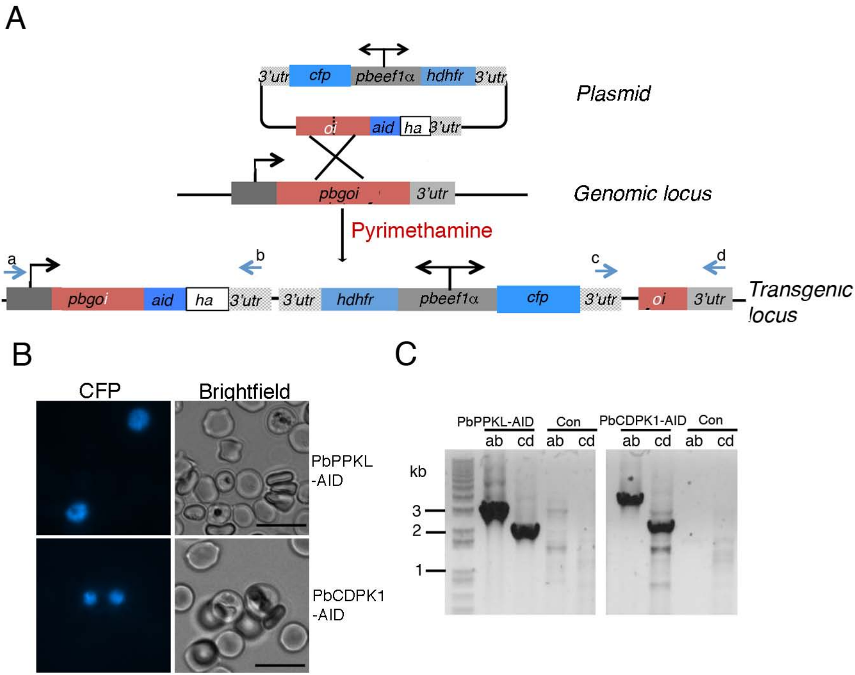

A
3'utr
cfp
pbeef1α
hdhfr
3'utr
Plasmid
oi
aid
ha
3'utr
pbgoi
3'utr
Genomic locus
Pyrimethamine
a
b
c
d
pbgoi
aid
ha
3'utr
3'utr
hdhfr
pbeef1α
cfp
3'utr
oi
3'utr
Transgenic locus
B
CFP
Brightfield
PbPPKL -AID
PbCDPK1 -AID
C
PbPPKL-AID
Con
PbCDPK1-AID
Con
ab
cd
ab
cd
ab
cd
ab
cd
kb
3
2
1

Figure S5

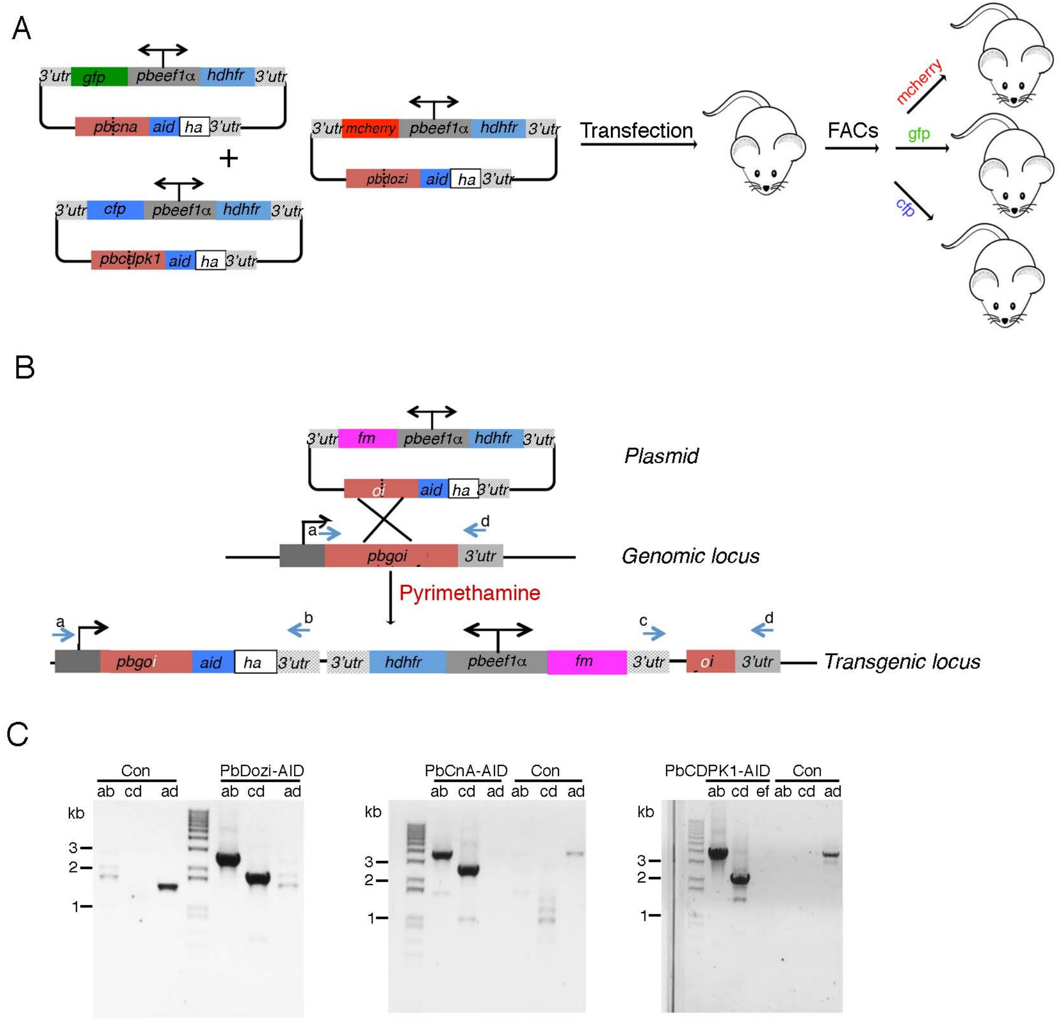
A
3'utr gfp pbeef1α hdhfr 3'utr
pbcna aid ha 3'utr
+
3'utr cfp pbeef1α hdhfr 3'utr
pbcdpk1 aid ha 3'utr
3'utr mcherry pbeef1α hdhfr 3'utr
pbdozi aid ha 3'utr
Transfection
FACs
mcherry
gfp
cfp
B
3'utr fm pbeef1α hdhfr 3'utr
oi aid ha 3'utr
Plasmid
a
d
pbgoi 3'utr
Genomic locus
Pyrimethamine
a
b
c
d
pbgoi aid ha 3'utr 3'utr hdhfr pbeef1α fm 3'utr oi 3'utr
Transgenic locus
C
Con
ab cd ad
PbDozi-AID
ab cd ad
kb
3
2
1
PbCnA-AID
ab cd ad
Con
ab cd ad
kb
3
2
1
PbCDPK1-AID
ab cd ef
Con
ab cd ad
kb
3
2
1

Supplemental Information

SUPPLEMENTAL FIGURE LEGENDS

Figure S1. Related to Figure 1

(A) Homologs of SCF complex comprising of Skp1 (S-phase associated protein 1), Cullin 1 and Rbx1 (Ring box protein 1) are present in *Plasmodium*. Homologs were initially identified by gene text searches on PlasmoDB, followed by confirmation on NCBI blastp search (http://blast.ncbi.nlm.nih.gov) against the non-redundant protein databases (nr).

(B) Alignment of *P. berghei* and *O. sativa* SKP1 using ClustalW illustrates a 64% identity (dark blue) and 93% homology (light blue) in the F-box binding region.

(C) Strategy for generation of marker-free (-ve) parent acceptor line expressing F-box protein TIR1 under the *hsp70* promoter. The *ostir1* gene expressed under the *hsp70* promoter is targeted to the non-essential *p230p* gene locus by double crossover and transgenic parasites are positively selected by providing pyrimethamine in drinking water to infected mice. Parasites showing successful integration (+ve) were cloned by limiting dilution, followed by negative selection using 5-fluorocytosine. Successful integration of *ostir1* and removal of the drug selection cassette containing *hdhfr* and *yfcu* genes was confirmed by PCR, with indicated primers [5' integration (ab): GU868/GU924); 3' integration (ef): (GU706/GU871 for *p48/45* 3'UTR and GU2050/GU871 for *p28* 3'UTR]; drug selection cassette removal (ab): (GU868/GU924)], and the transgenic line further cloned by limiting dilution to generate the *hdhfr* marker-free parent acceptor line.

(D) Expected PCR product sizes with indicated primers, as in Figure S1C, demonstrate proper integration of *hsp70*-driven *ostir1* plasmid at the *p230p* locus and removal of *hdhfr* and *yfcu* genes (left panel: *ostir1* with *p48/45* 3'UTR (pG230); middle panel: *ostir1* with *p28* 3'UTR (pG402); HP background strain is also shown. Right panel: myc-tagged OsTIR1 protein is expressed in transgenic *P. berghei* parasites (pG230 line is shown) before and after negative

selection. pG230 transfected parent line was used as control for all blood stage phenotyping, which include intraerythrocytic development, gametocyte maturation, activation and fertilization.

(E) PCRs, as in Figure S1C, showing proper integration of *hsp70*-driven *ostir1* (pG402) plasmid at the *p230p* locus in the GFP expressing 507cl1 line [5' integration (ab): GU868/GU869; 3' integration (ef): GU2050/GU871]. This line was used as a control for phenotypic analysis following gamete fertilization (ookinete motility, microneme secretion, sporozoite and EEF assays). Western blotting shows expression of OsTIR1-9myc protein (right panel). Enolase serves as the loading control.

(F) Schematic for single crossover generation of PbCnA-AID parasite strain. The targeting plasmid containing 1 kb C-terminus of *pbcna* gene is linearized by digestion with BsaBI restriction (dotted line) enzyme prior to transfection. Transgenic parasites were selected by pyrimethamine and correct integration checked by PCR with indicated primers [5' integration (ab): GU144/GU730; 3' integration (cd): GU533/GU147].

(G) PCRs, as in Figure S1F show upstream and downstream integration of *pbcna-aid-ha* targeting constructs in both parent lines (pG230, left panel; pG402, middle panel). Western blotting, followed by probing with α-HA antibody confirms expression of ~80 kDa protein corresponding to size of AID-HA-tagged PbCnA protein (pG230 background). Enolase serves as the loading control.

Figure S2. Related to Figure 2

(A) Efficient depletion of PbCnA-AID (α-HA) protein in schizonts when IAA (auxin) was added either to ring stage (R) or mature schizont stage parasites (S). Rings and schizonts were treated with IAA for 24 hrs or 30 min, respectively. Resulting PbCnA-AID protein depletion in these samples was examined by Western blotting. Enolase serves as the loading control.

(B) IAA treatment of ring-stage parasites has no effect on intraerythrocytic development. Ring stage infected blood was collected 15 min post-intravenous administration of mature schizonts.

PbCnA-AID parasites were cultured ± auxin smears made at 4 hr rings (R), 16 hr trophozoites (T) and 25 hr schizonts (S), stained with Giemsa and examined by light microscopy. Scale bar = 10 μm

(C) Representative images of attached merozoites (top panels) or invaded rings (bottom panels) examined 10 minutes post addition of purified merozoites to erythrocytes. Merozoites derived from mature schizonts (± auxin) were added to erythrocytes, supplemented with schizont media to a final hematocrit of 10% and shaken at 700 rpm. A 100μl aliquot was fixed, probed with DAPI and examined by microscopy.

(D) Merozoite attachment and invasion is unaffected in the control line (± auxin). The assay was performed as indicated in Figure 2C where a minimum of 10 fields containing ~350 erythrocytes were counted. 100% is the proportion of attached/invaded parasites without auxin treatment.

(E) Parasitemia resulting from successful invasion of erythrocytes was assessed 4 hours post addition of merozoites to erythrocytes. Experiment was performed as in Figure S2C where after shaking for 10 minutes, cells were placed in schizont media and incubated at 37°C. Four hours later, cultures were smeared and ring stage parasitemia was measured by Giemsa staining and light microscopy. 100% is parasitemia without auxin treatment (n=3 experiments; mean ± SEM; two tailed t test for paired observations: P <0.001 [***]).

(F) Mature gametocytes employed for phenotypic analysis have undetectable PbCnA-AID protein levels upon pre-treatment with IAA. Gametocytes were treated ± auxin for 30 min (at 37°C), collected, lysed, and PbCnA-AID protein levels were determined by Western blotting. Enolase serves as the loading control.

(G) Translation of *p28* and *rab11a* transcripts is unaffected by PbCnA-AID depletion, indicating normal translational control in activated female gametes (Mair et al., 2006). Gametocytes were pre-treated ± auxin for 30 min (at 37°C), activated at 21°C by addition of 100 μM xanthurenic acid in RPMI culture media. Parasite pellets were collected 3 hrs post-activation, lysed and

examined by Western blotting. Rabbit polyclonal antibodies against P28 and Rab11a were used to examine protein levels.

(H) Male (♂) and female (♀) gametocyte emergence upon activation. Infected cells were incubated ± auxin for 30 min, followed by FITC-conjugated α-mouse TER119 ± auxin for an additional 30 min. Cells were activated at 21°C by addition of 100 μM xanthurenic acid in RPMI culture media and emergence observed by DIC and fluorescence microscopy. Nuclei are DAPI stained. Scale bar = 10μm

(I) Reduction in the number of male gametocytes replicating their DNA when PbCnA-AID is depleted. Histogram showing proportion of male gametocytes undergoing DNA replication before and after activation, as in panel F. Prior to activation (0 min), males (♂) and females (♀) show similar DNA content. With activation, only males undergo DNA replication. Here, we assayed DNA replication at 8 min post-activation.

(J) Microgamete formation (arrows) is apparent in the proportion of male gametes undergoing exflagellation when PbCnA-AID is depleted.

Control line (Con) in all experiments is the OsTIR1-expressing line (pG230).

Figure S3. Related to Figure 3

(A) Reconstructed tracks of motile PbCnA-AID ookinetes in matrigel^{TM} ± auxin with frames collected every 10 seconds for 15 min.

(B) Oocyst numbers in mosquito midgut upon PbCnA-AID depletion in mature ookinetes. Auxin was added to parasite cultures six hours post induction of gametogenesis. Mature ookinetes were membrane fed to mosquitoes and seven days later midguts were dissected and GFP-positive oocysts were counted (n= minimum of 40 mosquitoes for each condition; mean ± SD). [****], *P* <0.0001.

(C) Immunofluorescence showing efficient depletion of PbCnA-AID-HA protein in sporozoites upon 500 μM IAA addition. Mouse α-HA antibody was used to detect PbCnA-AID-HA protein. Mouse secondary antibodies conjugated to Alexa 594-red was used. Parasites were fixed 90 min post-treatment with IAA. Brightfield images of the same section are also shown. Scale bar = 10 μm

(D) Representative images of 48 hr EEFs resulting from successful sporozoite invasion and colonization of HepG2 cells. EEFs were probed with α-CSP (circumsporozoite protein) and α-EXP1 (exported protein 1) and nuclear stain DAP1. Scale bar = 5 μm

Control line (Con) in all experiments is the OsTIR1-expressing line (pG402)

Figure S4. Related to Figure 4

(A) Schematic for single crossover strategy of generating of a transgenic parasite line with gene of interest (goi) tagged with *aid-2xha*. The targeting plasmid containing 1 kb C-terminus of goi is linearized by digestion with restriction (dotted line) enzyme prior to transfection. Transgenic parasites were selected by pyrimethamine and correct integration checked by PCR with indicated primers [5' integration (ab); 3' integration (cd)].

(B) CFP expressing parasites were recovered seven days post transfection of plasmid targeting *pbppkl* or *pbcdpk1* gene locus. Bright field images of same field are also shown. Scale bar = 10 μm

(C) PCRs, as in Figure S4A, show upstream and downstream integration of *pbppkl-aid* and *pbcdpk1-aid* targeting constructs. Integration checked by PCR with indicated primers [5' integration (ab): GU151/GU730 for *pbppkl,* GU2900/GU730 for *pbcdpk1*; 3' integration (cd): GU533/GU148 for *pbppkl,* GU533/GU2901 for *pbcdpk1*].

Figure S5. Related to Figure 5

(A) Schematic for generation of three transgenic lines in a single transfection experiment. 2.5 μg each of plasmids targeting *pbcna*, *pbdozi* and *pbcdpk1* loci were mixed with nucleofector™ and electroporated into schizonts. Schizonts were intravenously administered to naïve mice and 24 hours later the mice were given drinking water with Pyrimethamine. 7 days later fluorescent parasites were observed indicating expression of targeting plasmids. CFP, GFP and mCherry expressing Infected red blood cells were purified by fluorescence activated cell sorting and 50 parasites were intravenously administered to three naïve mice.

(B) Schematic for single crossover strategy of generating gene of interest (goi) tagged with AID transgenic parasite line. Transgenic parasites were selected by pyrimethamine and correct integration checked by PCR with indicated primers [5' integration (ab); 3' integration (cd) and non-modified locus contamination determined by (ad)]

(C) PCRs, as in Figure S5B, show upstream and downstream integration of *pbdozi-aid* (left-panel)*, pbcna-aid* (middle-panel) *and pbcdpk1-aid* (right-panel) targeting constructs in parasites lines isolated by FACS. Integration checked by PCR with indicated primers [5' integration (ab): GU730/GU1931 for *pbdozi,* GU144/GU730 for *pbcna,* GU730/GU2900 for *pbcdpk1*; 3' integration (cd): GU533/GU1371 for *pbdozi,* GU533/GU147 for *pbcna* GU533/GU2901 for *pbcdpk1* and unmodified locus contamination (ad): GU2212/GU1371 for *pbdozi,* GU144/GU147 for *pbcna,* GU2900/GU2901 for *pbcdpk1*]

Table S1. Primers used in this study. Related to Supplemental Experimental Procedures

Primer name	Sequence (5' to 3')
GU144	ATGAATTTTACATTTTTTTAAATCTTAATTATGTATTTTAGCATGC
GU147	CTATTTTACTTTACATATATACAACTCAAAATGGTATTG
GU148	TTGGAAATAATTCAAATTAGAGAAAAAAAATTCATGAGTTT
GU151	GTATATATATTTTTTTTGATTGAATTCCACTATATATTTTTTTGTTC
GU533	GATTAAGTTGGGTAACGCCAG
GU706	ACTAGTCCCGGGCTTAACATTCACATATATTAATAATTTTAAT
GU762	ATGGTCAAATGTATCTATATTATGTTCTATGG
GU730	GTTAAATGTGGGGTAAAAAAGAGT
GU868	GCAGATTATTTTATAAATAATTCATCAACC
GU869	GCTATTTACATGCATGTGCATGC
GU871	CATAAATCGACACACACAGAAATCGCC
GU924	CAAATTCAATGAACTTCTAATATGACTC
GU1371	CAATTATATTTGTGTTCGCATGTTTAATTGGG
GU1931	GGCATAATTCTTTCATCTCAATACGTTGC
GU2050	ATTCCCGGGCAATTGTTATCGCATATTGTAGGAA
GU2212	GTGTCGCTTCAAATGCTAATACCAATG
GU2578	GGTACCTTTTATAAAATTTTTATTTATTTATAAGCAAATATATAT
GU2579	TAATAAAGGGCACTTTAATTT
GU2580	GACCATATAAGAATTAACCCTTTACTTTTTTC
GU2581	AATTATTAATATATATGAATATATATACATCGTTGTATGCC
GU2598	TTTGTATAGTTCATCCATGCC
GU2599	ATGAGTAAAGGAGAAGAACTTTTCAC
GU2603	GACTACAAGACCCGATTACAATAGTTGG
GU2604	TTCATTTGATGGTCTCTTTTCATTTATACTATC
GU2607	GTGTCGCTTCAAATGCTAATACCAATG
GU2608	TAGTATAGATGGATGGATCTATTTCGTTAGGGATC
GU2847	GGTGTAATCAAAGTAAAAGTGCAAATGATG
GU2848	AAATGTTTTATGGTCACAAATTTTGTGC
GU2849	TGGAGCCCCATAATTTAATTCTCTC
GU2900	GTCATCCCTAACTACCCAAG
GU2901	TTCTTTTTCTCTCTTATTTCTATTCCTC

SUPPLEMENTAL EXPERIMENTAL PROCEDURES

Ethics statement for animal experimentation

All infections were performed on Theiler's original (TO) mice (age 6–8 weeks; weight 25–30 g) (Philip et al., 2013) and animal procedures were carried out according to UK Home Office regulations and protocols were approved by the University of Glasgow Ethics Committee (Project licence 60/6443 to A.P.W.).

Generation of transgenic parasites

All transgenic parasite lines were generated by electroporation of gene-targeting plasmids into purified schizonts followed by intravenous administration into mice according to previously established protocols (Philip et al., 2013). *P. berghei* ANKA HP and 507cl1 (GFP expressing under *ee1f*α promoter in the HP background (Janse et al., 2006) lines were obtained from C. Janse at Leiden University Medical Centre (Leiden, Netherlands).

We constructed plasmids using standard molecular biology techniques. All primer sequences are provided in Table S1. The plasmid (pG230) used to generate the OsTIR1 expressing parent line (in *P. berghei* ANKA HP background) was derived from the pG0148 vector (Sinha et al., 2014). The *cfp* gene in pG0148 was replaced by *ostir1-9myc* [amplified from BYP6743 plasmid (Yeast Genetic Resource Center, Osaka, Japan) with primer pair GU2102/GU2063] using the XhoI and SmaI restriction sites between the *hsp70* (PBANKA_071190) promoter and *p45/48* 3′ UTR. The pG230 plasmid contains target regions for double crossover integration into the locus for the non-essential gene *p230p* and also a negative selection cassette to generate a marker-free line (see Figure S4D) (Orr et al., 2012). This line was used as control and an acceptor to generate the *pbcna-aid* line to examine intraerythrocytic development and gametocyte-ookinete transition assays. A second OsTIR1 expressing parent line was generated where the *p45/48* 3'UTR was replaced by the *p28* 3'UTR (pG402), which represses translation of protein in the gametocyte till 90 min post-activation.

Following transfection into *P. berghei*, the pG402-based parent line gave higher ookinete yields and was therefore used as an acceptor to generate *pbcna-aid* lines to examine ookinete secretion and motility assays, and sporozoite invasion assays. Integration of *hsp70-ostir1-p45/48*–at the *p230p* locus was tested at 5' (GU868/GU924) and 3' (GU871/GU706) and negative selection to remove the *hdhfr* selectable marker was confirmed (GU868/GU924). The 3' integration of the TIR1 line with *p28* 3'UTR was also confirmed (GU871/GU2050). The pG402 plasmid was also transfected into the GFP-expressing 507cl1 parasites and integration at the *p230p* locus was confirmed at 5' (GU868/GU869) and 3' (GU871/GU2050) ends. This GFP-fluorescent parasite line was used as control for ookinete motility, microneme secretion, sporozoite and EEF assays.

The *aid* degron tagging plasmid (pG390) was generated by amplification of the degron CDS from BYP6739 (Yeast Genetic Resource Center, Osaka, Japan) using primer pair BamHI-GU2065/BamHI-GU2066, followed by ligation into the PL31-HA plasmid (Philip et al., 2012). A GFP expressing version of the degron plasmid (pG362) was also generated by replacing the drug selection cassette (*tgdhfr* promoter-*tgdhfr*) with the bidirectional *pbee1f*α promoter driving both *gfp* and *hdhfr* expression. The pG362 plasmid was generated as detailed below. The PL0035 vector (Philip et al., 2012) was digested with NheI and re-ligated to remove the *yfcu pbdhfr*- 3′ UTR (pL0035a). Part of *pbee1f*α promoter was amplified by (SacII)-GU2578/(SpeI)-GU2579 from *P. berghei* genomic DNA, digested with SacII and SpeI, and ligated into pL0035a (also digested with SacII/SpeI) resulting in the reconstitution of the complete bidirectional *pbee1f*α promoter driving expression of the *hdhfr* selectable marker (pL0035b). Primer GU2578 also introduced a KpnI site upstream of the SacII site at the 5' end of *pbeef1*α promoter. The 3'UTR from the *P. berghei* gene *cam* (PBANKA_101060) was amplified with (PstI)-GU2580/(KpnI)-GU2581 from genomic DNA, digested with KpnI and PstI and ligated into pL0035b (pL0035c). The *gfp* gene was amplified from pL0031-*pbppkl* vector (Philip et al., 2012)

using (KpnI)-GU2598/(KpnI)-GU2599, digested with KpnI and ligated into pL0035c (also digested with KpnI) between the *cam*-3'UTR and *pbee1f*α promoter. Finally, the cassette was digested with PstI and NheI and ligated into the *aid* degron expressing plasmid pG390, where the drug selection cassette (*tgdhfr* promoter-*tgdhfr*) had been removed with a PstI/NheI digest. This resulted in the *aid-p48/45* 3′ UTR-*pbdhfr* 3′ UTR *-hdhfr- ee1f*α*-gfp-calmodulin* 3′ UTR plasmid (pG362). The two additional fluorescence marker expressing plasmids were generated by replacing the gfp ORF with either cfp (pG363) or mcherry (pG364).

The *pbcna*-*aid*-2x*ha* tagged construct was generated by cloning 1 kb of the *pbcna* 3' end up to, but not including the stop codon, into pG390 and pG362 plasmids, amplified by using primers (SacII)-GU2603 and (BamHI)-GU2604. Correct integration of the targeting plasmid and gene modification was verified by integration PCR (5'- GU144/GU730; 3'-GU533/GU147) and Western blotting. The pG390-*pbcna-aid* transfected into the pG230 based parent line (non-fluorescent), was used for all blood stage phenotyping, which include intraerythrocytic development, gametocyte maturation, activation and fertilization. The pG362-*pbcna-aid* transfected into the pG402-based parent line (GFP-fluorescent) was used for phenotyping post-fertilization stages, which include ookinete motility, microneme secretion, sporozoite and EEF assays.

Similarly *pbppkl*, *pbcdpk1* and *pbdozi* tagged with *aid*-2x*ha* constructs were generated by cloning 1 kb of the gene 3' end up to, but not including the stop codon into pG363 (for *pbppkl* and *pbcdpk1*) and pG364 (for *pbdozi*) amplified by using (SacII)-GU2847/(XhoI)-GU2848 for *cdpk1*; (SacII)-GU762/(XhoI)-GU2849 for *ppkl*; (SacII)-GU2607/(NotI)-GU2608 for *dozi*. All plasmids were transfected into the pG230 based parent line expressing OsTIR1 controlled by the hsp70 promoter and pb48/45 3'utr. Correct integration of the targeting plasmid was confirmed by integration PCR (5' integration: GU730/GU1931 for *pbdozi,* GU144/GU730 for *pbcna,* GU730/GU2900 for *pbcdpk1* ; 3' integration: GU533/GU1371 for *pbdozi,* GU533/GU147

for *pbcna,* GU533/GU2901 for *pbcdpk1* and unmodified locus contamination : GU2212/GU1371 for *pbdozi,* GU533/GU147 for *pbcna,* GU2900/GU2901 for *pbcdpk1*)

Protein depletion assay

To determine degradation kinetics of PbCnA-AID protein, mature schizonts (enriched on a 55 % Nycodenz gradient), gametocytes (isolated on a LD-50 MACs column, Miltenyi) or salivary gland sporozoites were incubated with 500 μM Indole 3-acetic acid (IAA) dissolved in schizont media (RPMI1640 containing 25 mM HEPES, 5 mM hypoxanthine, 20% FCS, 10 mM sodium bicarbonate, 100 U/ml penicillin and 100 μg/ml streptomycin) for indicated periods of time. The cells were either immediately snap frozen in liquid nitrogen for Western blot analysis or fixed with 4% EM grade paraformaldehyde (in PBS) and smeared onto glass slides for immunofluorescence assays. To test if protein depletion is dependent on the proteasome, parasites were pre-incubated with 1 μM MG132 (Sigma) for 1 hr prior to treatment with IAA. IAA concentration for all assays performed in this study is 500 μM.

Parasite developmental assays

To determine the effect of PbCnA protein depletion on schizont development, purified schizonts were intravenously administered to mice, followed by isolation of rings by cardiac puncture 30 min later. The parasites were cultured in schizont media for 25 hrs ± IAA and schizont development and merozoite count determined by Giemsa smears. To assess gametocytemia, purified schizonts were intravenously administered to mice, followed by isolation of rings by cardiac puncture 30 min later. Blood was passaged through an LD-50 MACs column to remove mature gametocytes. The flow-through was cultured for 36 hrs ± IAA and gametocytemia was determined by Giemsa smears.

Western blotting and immunofluorescence assays

Parasite pellets were lysed in RIPA buffer (50 mM Tris-HCl, pH 7.5, 150 mM NaCl, 2 mM EDTA, 1% NP-40, 0.1% SDS) supplemented with protease inhibitor (Roche) and clarified lysates were suspended in Laemmli sample buffer and separated on a 10% SDS-polyacrylamide gel. Samples were transferred to nitrocellulose membranes (GE Healthcare) and probed with mouse α-HA (1:1000 in 5% milk/PBS), followed by goat anti-mouse secondary antibody (1:5000 in 5% milk/PBS), and visualized with ECL kit (Pierce) or ECL Advance kit (GE healthcare). Other antibody dilutions were as follows: α-enolase (peptide: KTYDLDFKTPNNDK, rabbit polyclonal at 1:1000), α-chitinase (peptide: HTEKQYKSLSHVDALC, rabbit polyclonal at 1:4000), α-CTRP (peptide: LNGGETPHNSNMEFENVENNDGIIEEENEDFEVIDANDPMW, rabbit polyclonal at 1:4000), α-p28 (peptide: VSKPQAPGTGSETP rabbit polyclonal at 1:4000), α-rab11a (peptide: HTEKQYKSLSHVDALC, rabbit polyclonal at 1:1000), α-WARP (peptide: CNKNNPSSLTSERKTTIKN, rabbit polyclonal at 1:4000) and α-GFP (mouse monoclonal 1:4000, Roche Diagnostics). All rabbit polyclonal antibodies were generated at Proteintech group (Chicago, USA).

For IFAs, fixed parasites were smeared onto glass slides and processed as previously shown (Philip et al., 2012). Primary antibody dilutions were as follows: mouse α-HA antibody (1:400, Sigma), mouse α-tubulin (1:1000), mouse α-P25-Cy5 (1:1000). Secondary antibodies (Alexa fluor 594 or 488, Molecular Probes) were used at 1:2000.

Erythrocyte invasion assays

For erythrocyte invasion assays, nycodenz-enriched mature schizonts were incubated ± IAA for 30 min. The schizonts were ruptured by serially passing the cells through a 5 μm (Acrodisc) and a 1.6 μm (Puradisc, Whatman) to release merozoites. For *in vivo* invasion assays merozoites were subsequently intravenously injected into mice. 5-6 tail blood drops were collected 15 min

post-injection and incubated in schizont media without IAA for 16 hrs. Cells were stained with 2 μM Hoescht in rich PBS (20 mM HEPES, 20 mM glucose, 4 mM $NaHCO_3$, 0.1% BSA) for 30 min at 37°C. Cells were pelleted and resuspended in 5ml FACS buffer (2 mM HEPES, 2 mM glucose, 0.4 mM $NaHCO_3$, 0.01% BSA, 2.5 mM EDTA) and analysed on a CyAn ADP 9 colour flow cytometer (Beckman Coulter) equipped with 405-nm, 488-nm and 642-nm solid-state lasers. Data was analysed on FlowJo software to determine parasitemias. For duplexed invasion assays PbCnA-AID (expressing GFP) and PbCDPK1-AID (expressing CFP) schizonts were combined and incubated ± IAA for 30 min and subsequently intravenously injected into mice. Further analysis was performed as described above for *in vivo* invasion assay, except cells were stained with Vybrant® DyeCycle™ Ruby DNA dye (Life technologies) in rich PBS.

For *in vitro* attachment/invasion assays purified merozoites (± IAA;) were added to mouse erythrocytes supplemented with schizont media (10 % hematocrit; 500μl final volume), ± Cytochalisin D at 1μM and incubated at 37°C with vigorous shaking (700 rpm) for 10 minutes. A 100μl aliquot was fixed in 1.4 ml of 2 % PFA and 0.015 % EM grade glutaraldehyde for 30 min at room temperature. Cells were washed 3X with PBS and smeared on glass slides, mounted with Vectashield™ + DAP1 and examined by fluorescence and light microscopy. A minimum of 10 fields with ~ 350 cells were inspected to determine attached/invaded parasites. To determine parasitemia of successfully invaded parasites, 200 μl of the remaining cells post-shaking were placed in schizont media and allowed to develop for 4 hours. Ring stage parasitemia was calculated from smears by Giemsa staining and light microscopy.

To determine PbAMA1 processing, purified merozoites from schizonts (± IAA) were obtained by mechanical rupture as described for the invasion assay. Assay was performed with minor modifications of a previously described protocol (Singh et al., 2014). Merozoites from the PbCnA-AID expressing line were suspended in buffer mimicking intracellular conditions (IC: 5 mM NaCl, 140 mM KCl, 2 mM EGTA, 1 mM MgCl2, 5.6 mM glucose and 25 mM Hepes, pH

7.2). Sample was split, spun at 3300 g and resuspended in 100μl of either IC or extracellular buffer (EC: 140 mM NaCl, 5 mM KCl, 1 mM CaCl2, 1 mM MgCl2, 5.6 mM glucose and 25 mM Hepes, pH 7.2) for 10 min at 37°C. Cells were spun again at 3300 g and supernatant and pellet were separated, and heated in Laemmli sample buffer for 15 minutes at 75°C. Lysates were separated on a 4-20 % gradient gel and PbAMA1 protein levels and processing was examined by Western blot. Blots were probed with rat mAb28G2 (Narum and Thomas, 1994) at 1:4000 dilution and goat anti-rat secondary (Life technologies).

Sporozoite invasion assays

For sporozoite-HepG2 infection assays, the GFP-expressing PbCnA-AID parasite line was exploited. Sporozoites were isolated 22 days post-transmission from infected mosquito salivary glands. Mosquitoes were sterilized in 70% ethanol followed by a sterile PBS wash. Salivary glands (SGs) were dissected in RPMI supplemented with 100 U/ml penicillin and 100 μg/ml streptomycin and kept on ice. SGs were ruptured by 25 pestle strokes in at 1.5ml Eppendorf centrifuge tube, debris pelleted at 100 x g. The supernatant fraction containing the sporozoites was isolated and examined under a hemocytometer. $1x10^4$ sporozoites were treated ± IAA (in schizont media) for 90 min at 21°C and then incubated with HepG2 hepatocyte cells (plated at ~ 70% and 40% confluency for sporozoite invasion and EEF development respectively) in chamber of a 4-well Nunc™Labtek™ slide (Thermoscientific™).

Sporozoite invasion was examined as previously described (Sinnis et al., 2013). 2 hours after addition of sporozoites to HepG2 cells, media was removed and cells fixed in 4% PFA in PBS for 1 hr at room temperature. Samples were blocked in 2 % BSA/PBS for 1 hr at 37°C and incubated with α-PbCSP (mAb3D11) at 1:4000 dilution (in 2 % BSA/PBS) overnight at 4°C. After 3X washes with PBS, cells were incubated with goat α-rat secondary conjugated to Alexa 488. Following 3X PBS washes, cells were permeabilized with ice cold methanol for 15 minutes,

blocked in in 2 % BSA/PBS for 1 hr at 37°C and incubated with α-PbCSP (mAb3D11) at 1:500 dilution for 1 hr at 37°C. Cells were washed 3X in PBS, incubated with goat α-rat secondary conjugated to Alexa 594. Following 3X PBS washes samples were mounted in Vectashield™ + DAPI. Sporozoites were visualized on Leica M205 FA fluorescence stereomicroscope. Extracellular sporozoites appear red while green represents both extracellular and intracellular sporozoites. Proportion of invaded sporozoites were calculated as (# Green sporozoites – # Red sporozoites)/ # Green sporozoites. A minimum of 6 fields per experimental condition was examined.

To assess EEF development, sporozoites were also handled as above. 90 min after addition of sporozoites to HepG2 hepatocytes,cells were washed 3x with 1 ml DMEM (containing 2 mM L–glutamine,10% fetal bovine serum and 2% penicillin/streptomycin) and incubated with DMEM supplemented ± IAA. Media was replenished 1 and 2 days post infection, and EEFs (GFP expressing) were counted on a DeltaVision Epifluorescence microscope (Applied Precision) under a 20x objective. Additionally 48 hr EEFs were fixed in 4% PFA in PBS for 1 hr at room temperature and permeabilized with ice cold methanol at room temperature for 15 min. Samples were blocked in 2 % BSA/PBS for 1 hr at 37°C and incubated with α-PbCSP (mAb) at 1:4000 dilution and α-PbEXP1 (at 1:500 dilution) (van de Sand et al., 2005) overnight at 4°C. Cells were washed 3X in PBS, incubated with goat α-rat secondary conjugated to Alexa 488 and goat α-chicken secondary conjugated to Alexa 594 for 1 hr at 37°C. Following 3X PBS washes samples were mounted in Vectashield™ + DAPI. EEFs were examined on a DeltaVision Epifluorescence microscope (Applied Precision) under a 100X objective. Images were acquired, recorded and deconvulated using softWoRx (Applied Precision).

Phenotypic analysis of sexual stage development

Infections were performed by intraperitoneal administration of cryopreserved, cloned parasites into phenylhydrazine (1.25 mg)-treated mice. Enriched gametocytes were obtained by sulfadiazine (35 ml/l) treating the infected mice for 48 hrs (Beetsma et al., 1998). Gametocytes were pre-incubated in schizont media (with IAA) for 30 min at 37°C prior to activation. Cultures were then incubated with ookinete media (RPMI1640 containing 25 mM HEPES, 5 mM hypoxanthine, 20% FCS, 10 mM sodium bicarbonate, 100 μM xanthurenic acid at pH 7.6; ±IAA) for further analysis. DNA replication, exflagellation assays and ookinete conversion rates were assessed as previously described (Laurentino et al., 2011; Philip et al., 2012). For DNA replication assay, gametocytes were purified on LD-50 MACs column, pre-incubated with IAA and transferred to ookinete media for activation. At 0 and 8 min following addition of ookinete media, cells were fixed in 0.25% glutaraldehyde/PBS solution and stained with 2 μM Hoechst-33258 and DNA content was analysed by FACs on CyAn ADP 9 colour flow cytometer. For gametocyte emergence assay, gametocytes were pre-incubated ± IAA (45 min) with α-mTER119-FITC (1:1000 dilution, 30 min), followed by transfer to ookinete media. Images were acquired in the GFP and brightfield channels (over 5-15 min post-activation) to determine gamete emergence. 100 gametocytes were counted per condition.

Motility and midgut invasion in ookinetes

Ookinetes embedded in Matrigel (BD Biosciences) were incubated for 1 hr at 21°C before imaging. Time-lapse movies were acquired every 10 seconds for 15 min. For the duplexed motility assays time-lapse movies were acquired every 10 seconds for 10 min employing the GFP and mCHERRY filter sets (0.5 second exposure for each). Ookinete speeds were calculated on Fiji software using the MtrackJ plugin (Meijering et al., 2012).

For ookinete feed assays in the mosquito purified ookinetes were produced and handled as described in main experimental procedures. Equal amounts of purified ookinetes (treated ± IAA

from 4-6 hrs post-activation) were mixed with 750 μl of mouse blood and 500 μl ookinete media. The ookinete and blood mixture were fed to mosquitoes via a membrane feeder (maintained at 37°C) for 20 minutes. Midguts were dissected 7 days post-feeding and oocyst numbers were counted.

Flow cytometric isolation of transgenic parasites

After successful integration of all three plasmids was confirmed by PCR, parasites individually expressing the 3 fluorescent markers (GFP, CFP and mCHERRY) were propagated in a mouse until parasitemia reached 0.2-1.0%. Infected blood was examined under the fluorescence microscope to exclude multiply infected erythrocytes. One drop of tail blood was added to 2 ml schizont media, pelleted and resuspended in 5 ml FACs buffer, filtered through a BD FACs tube with cell strainer cap to prevent cell aggregation. Cell sorting was performed on a BDFACSAria III cell sorter (4 lasers: Blue 488nm, Yellow/Green 631nm, Red 640nm and Violet 405nm). Cells were sorted through a 70 micron nozzle at 70 psi and 10,000 events/second. Forward and side scatter gating was initially used to exclude both small cells (platelets or ruptured parasites) and leukocytes. Fluorescent parasites were detected using the respective filter sets: mCherry at 610/20 BP filter and 600 LB mirror; GFP at 530/30 BP filter and 502 LP mirror; CFP at 450/40 BP filter and collected into Eppendorf tubes with RPMI media. 1000 IRBCs for each fluorescence were collected in RPMI and 50 cells were intravenously injected into a mouse. 5-7 days later parasites were recovered and used for further analysis.

SUPPLEMENTAL REFERENCES

Beetsma, A.L., van de Wiel, T.J., Sauerwein, R.W., and Eling, W.M. (1998). Plasmodium berghei ANKA: purification of large numbers of infectious gametocytes. Experimental Parasitology *88*, 69–72.

Janse, C.J., Franke-Fayard, B., Mair, G.R., Ramesar, J., Thiel, C., Engelmann, S., Matuschewski, K., van Gemert, G.-J., Sauerwein, R.W., and Waters, A.P. (2006). High efficiency transfection of Plasmodium berghei facilitates novel selection procedures. Molecular and Biochemical Parasitology *145*, 60–70.

Mair, G.R., Braks, J.A.M., Garver, L.S., Wiegant, J.C.A.G., Hall, N., Dirks, R.W., Khan, S.M., Dimopoulos, G., Janse, C.J., and Waters, A.P. (2006). Regulation of sexual development of Plasmodium by translational repression. Science *313*, 667–669.

Meijering, E., Dzyubachyk, O., and Smal, I. (2012). Methods for cell and particle tracking. Meth. Enzymol. *504*, 183–200.

Narum, D.L., and Thomas, A.W. (1994). Differential localization of full-length and processed forms of PF83/AMA-1 an apical membrane antigen of Plasmodium falciparum merozoites. Mol Biochem Parasitol *67*, 59-68.

Orr, R.Y., Philip, N., and Waters, A.P. (2012). Improved negative selection protocol for Plasmodium berghei in the rodent malarial model. Malar J *11*, 103.

Philip, N., Orr, R., and Waters, A.P. (2013). Transfection of rodent malaria parasites. Methods Mol. Biol. *923*, 99–125.

Sinha, A., Hughes, K.R., Modrzynska, K.K., Otto, T.D., Pfander, C., Dickens, N.J., Religa, A.A., Bushell, E., Graham, A.L., Cameron, R., et al. (2014). A cascade of DNA-binding proteins for sexual commitment and development in Plasmodium. Nature *507*, 253–257.

van de Sand, C., Horstmann, S., Schmidt, A., Sturm, A., Bolte, S., Krueger, A., Lutgehetmann, M., Pollok, J.M., Libert, C., and Heussler, V.T. (2005). The liver stage of Plasmodium berghei inhibits host cell apoptosis. Mol Microbiol *58*, 731-742.
